# TRIM21 facilitates inflammasome assembly and contributes to autoinflammatory disease

**DOI:** 10.1038/s41467-026-73350-3

**Published:** 2026-05-22

**Authors:** Jessica Carriere, Chawon Yun, Sonal Khare, Sana Ismaeel, Elisabeth Jäger, Vinicius Dantas Martins, Kaiden A. Sims, Lan H. Chu, Huyen Nguyen, Lucia de Almeida, Janset Onyuru, Justin Ruiz, Hemisha Khatri, Savita Devi, Jae Jin Chae, Daniel L. Kastner, Lori Broderick, Hal M. Hoffman, Andrea Dorfleutner, Christian Stehlik

**Affiliations:** 1 Department of Pathology and Laboratory Medicine, Cedars Sinai Medical Center, Los Angeles, CA USA; 2 Division of Rheumatology, Department of Medicine, Feinberg School of Medicine, Northwestern University, Chicago, IL USA; 3 Department of Pediatrics, University of California San Diego and Rady Children’s Hospital, La Jolla, CA USA; 4 National Human Genome Research Institute, National Institutes of Health, Bethesda, MD USA; 5 Department of Biomedical Sciences, Cedars Sinai Medical Center, Los Angeles, CA USA; 6 Samuel Oschin Comprehensive Cancer Institute, Cedars Sinai Medical Center, Los Angeles, CA USA; 7 Present Address: Department of Orthopedic Surgery, and Nano-based Disease Control Institute, Korea University College of Medicine, Seoul, Republic of Korea; 8 Present Address: Tempus AI, Chicago, IL USA; 9 Present Address: Department of Immunology, University of Washington, Seattle, WA USA; 10 Present Address: Graduate Program in Biomedical Science and Translational Medicine, Cedars Sinai Medical Center, Los Angeles, CA USA; 11 Present Address: Stanley Manne Children’s Research Institute, Ann & Robert H. Lurie Children’s Hospital of Chicago, Chicago, IL USA; 12 Present Address: Department of Microbiology and Immunology, University of Miami Miller School of Medicine, Miami, FL USA; 13 Present Address: Arrowhead Pharmaceuticals, San Diego, CA USA; 14 Present Address: Department of Biotechnology, Indian Institute of Technology Hyderabad, Sangareddy, Telangana India

**Keywords:** Inflammasome, Chronic inflammation, Autoimmunity

## Abstract

Inflammasomes are cytosolic multiprotein complexes facilitating the maturation and release of the inflammatory cytokines interleukin (IL)−1β and IL-18 and pyroptosis. ASC (apoptosis-associated-speck-like protein containing a CARD) is the central inflammasome adaptor. ASC polymerization is crucial for inflammasome assembly, and ASC particle release propagates inflammasome responses to bystander cells. However, control of inflammasome and ASC particle assembly to limit chronic inflammation and the emergence of autoinflammatory diseases is still incompletely understood. Here, we show that the E3 ubiquitin ligase TRIM (tripartite-motif-containing protein) 21, a common autoantigen in autoimmune diseases, is involved in inflammasome assembly. Specifically, TRIM21 binds to and ubiquitinates ASC to facilitate ASC/NLRP3 interactions, ASC polymerization and the release of ASC/TRIM21-containing particles during pyroptosis in human and mouse macrophages. Furthermore, we detect systemic ASC/TRIM21 particles and autoantibodies in human and mouse autoinflammatory disease. Thus, our findings highlight a previously unrecognized role of TRIM21 as an inflammasome component and driver of autoinflammation.

## Abstract (web summary)

Tight regulation of inflammasome assembly and activation is essential for controlling chronic inflammation and preventing the emergence of inflammatory pathologies. Here, the authors identify the E3 ubiquitin ligase TRIM21 as a regulator of ASC polymerization and ASC particle release during pyroptosis. Importantly, they identify the presence of TRIM21+/ASC+ particles and anti-TRIM21 autoantibodies in mouse models and human patients, linking inflammasome-driven inflammation to systemic autoimmunity.

## Introduction

Inflammation is a fundamental immune defense mechanism in response to infectious and sterile stress or danger signals called pattern-associated molecular patterns (PAMP) and danger-associated molecular patterns (DAMP), respectively. To facilitate the eradication of pathogens, initiation of wound healing, and re-establishment of homeostasis, pattern recognition receptors (PRR) of tissue-resident macrophages sense PAMPs and DAMPs and trigger pro-inflammatory cytokine and chemokine expression and release, to activate local immune cells and recruit neutrophils and monocytes. Activation of several intracellular PRRs belonging to the (NOD)- and leucine-rich repeat (LRR)-containing receptor (NLR) protein family results in the initiation of inflammasome assembly and activation. NLRs share a tripartite domain organization with an N-terminal pyrin domain (PYD) or caspase recruitment domain (CARD), a central nucleotide-binding NACHT domain (NBD), and a C-terminal regulatory LRR. In addition to several NLRs, other PYD- or CARD-containing proteins, including AIM2, Pyrin, and CARD8, can also initiate inflammasome assembly. Activation of inflammasomes requires two steps^1^. First, priming triggers a transcriptional response and post-translational modifications of inflammasome components and substrates. Second, PRR activation and clustering, ASC recruitment as well as nucleation of ASC polymerization initiate inflammasome activation. The self-perpetuating ASC polymerization proceeds by a non-reversible prion-like mechanism^2,3^. Polymerized ASC subsequently recruits pro-caspase-1, thereby triggering its autoactivation through an induced-proximity mechanism. Then active caspase-1 proteolytically matures pro-IL-1β and pro-IL-18, which is a prerequisite for their release. Additionally, it cleaves gasdermin D (GSDMD), resulting in the generation of an N-terminal GSDMD fragment (GSDMD-N) that inserts into the cell membrane. GSDMD-N polymerization generates pores that facilitate IL-1β, IL-18, and polymerized ASC particle egress as well as pyroptotic cell death^4^. Polymerized ASC particles are stable, and released ASC particles promote caspase-1 activation in the extracellular space, and are also phagocytized by bystander macrophages, where they trigger inflammasome responses independently of PAMP/DAMP sensing to propagate and perpetuate inflammation. Polymerized ASC particles are present in the circulation of patients with autoinflammatory diseases, including cryopyrin-associated autoinflammatory periodic syndrome (CAPS), an NLRP3 inflammasome-driven autoinflammatory disease^5,6^.

TRIM proteins are the largest family of RING-containing E3 ubiquitin ligases. They regulate cellular processes by attaching ubiquitin primarily to lysine residues of target proteins, thereby determining their fate^7^. TRIM proteins share a common tripartite domain organization with (i) a RING domain harboring the E3 ubiquitin ligase activity; (ii) one or two regulatory b-BOX domains; and (iii) a coiled-coil domain involved in oligomerization. Members of the TRIM family are distinguished by their C-terminal protein recognition and effector domains like the PRY/SPRY for TRIM21, which is also known as Ro52. It was originally identified as a common autoantigen in Sjögren’s Syndrome and Systemic Lupus Erythematosus^8^, and detection of anti-TRIM21 autoantibodies is widely used as diagnostic marker for autoimmune diseases^9^. Ubiquitination of TRIM21 target proteins contributes to the regulation of diverse cellular processes including innate immunity by NF-κB and type I interferon regulation as well as by functioning as an IgG Fc-binding protein^10–16^. Recently, TRIM21 has been shown to promote GSDMD expression and oligomerization to regulate pyroptosis^17^. However, any role of TRIM21 in inflammasome assembly in macrophages and autoinflammatory disease remains elusive.

Here, we identify TRIM21 as a component of the inflammasome scaffold, which regulates inflammasome assembly through interaction with and ubiquitination of ASC, and regulation of ASC recruitment to NLRP3 and ASC polymerization. Consequently, TRIM21 facilitates the activation of inflammasomes, IL-1β and IL-18 release as well as pyroptosis. Subsequently, TRIM21 is released from macrophages as part of polymerized ASC particles during inflammasome activation and pyroptosis. Importantly, we identify the presence of TRIM21 and anti-TRIM21 autoantibodies in the serum of CAPS and familial Mediterranean fever (FMF) mice and in the plasma of human CAPS patients, linking TRIM21 and anti-TRIM21 autoantibodies, which have a diagnostic role for the diagnosis of major autoimmune diseases, also to autoinflammatory disease.

## Results

### TRIM21 binds and ubiquitinates the inflammasome adaptor ASC

**Fig. 1:**
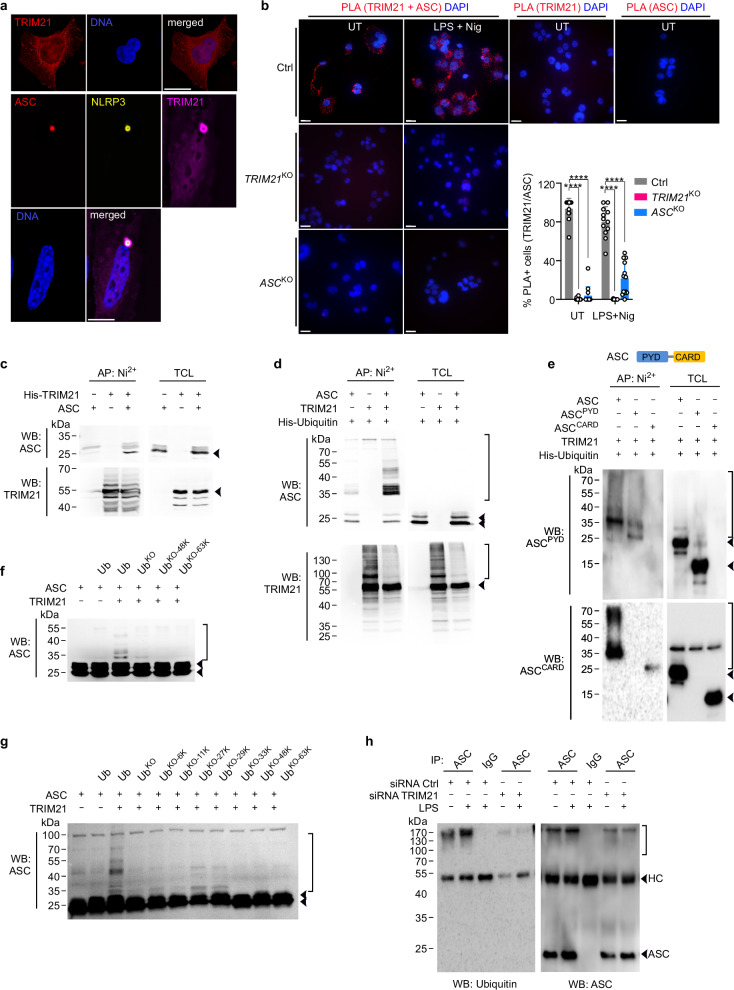
TRIM21 binds and ubiquitinates the inflammasome adaptor ASC. **a** Representative confocal microscopy images of immunostained HEK293 cells that were transfected with epitope-tagged TRIM21 (top panel), or TRIM21, ASC and NLRP3 (middle and bottom panel). Scale bar: 10 μm. **b** Microscopy of PLA (red) between TRIM21 and ASC, and DAPI using PMA-differentiated Cas9-expressing Ctrl, *TRIM21*^KO^, and *ASC*^KO^ THP-1 cells left untreated (UT) or primed with LPS (0.5 μg mL^−1^, 2 h) and activated with nigericin (Nig, 10 μM, 20 min) and quantification of PLA^+^ cells per frame (*n* = 12 frames, mean ± s.d., one-way ANOVA with Dunnett’s post-test). Scale bar: 20 μm. **c** HEK293 cells were transfected with His-TRIM21 and ASC for affinity purification (AP) of His-TRIM21 with immobilized Ni^2+^ beads before SDS-PAGE and Western blot (WB) for ASC and TRIM21 of affinity-purified and total cell lysate (TCL) samples. **d**, **e** WB of ubiquitinated proteins after transient transfection of HEK293 cells with TRIM21, His-Ubiquitin, and ASC (**d**) or ASC^PYD^ and ASC^CARD^ (**e**). His-tagged ubiquitinated proteins were affinity-purified (AP) with immobilized Ni^2+^ beads and analyzed alongside TCL for ASC and TRIM21 as indicated. **f**, **g** WB of ASC after transient transfection of HEK293 cells with TRIM21, ASC, and Ubiquitin, Ubiquitin Lys KO (Ub^KO^), or Ub^KO^ with a single Lys intact (Ub^KO−6K,−11K,−27K,−29K,−33K,−48K,−63K^). **c**–**g** Brackets indicate ubiquitinated proteins. Arrowheads indicate non-modified proteins. **h** Human primary macrophages were transfected with Ctrl siRNA or *TRIM21* siRNA and left untreated or primed with LPS (100 ng mL^−1^, 90 min), and TCL were immunoprecipitated (IP) with anti-ASC antibodies or IgG control and immunoblotted for ASC and ubiquitin. Brackets indicate ubiquitinated ASC; HC heavy-chain. Results representative of *n* = 3 experiments.

ASC polymerization is a key event in the inflammasome response^2,3^. Upon investigation of the possible molecular mechanisms involved in this process, we identified TRIM21 as a binding partner of the inflammasome adaptor ASC and potentially an inflammasome component. To determine whether TRIM21 could be regulating the NLRP3 inflammasome, we first analyzed its subcellular localization by immunofluorescence after overexpression along with NLRP3 and ASC in HEK293 cells, which lack endogenous inflammasome components. TRIM21 expression on its own was dispersed in the cytoplasm, but upon TRIM21, ASC, and NLRP3 co-expression, all three proteins co-localized in an aggregated perinuclear structure (Fig. 1a), suggesting that TRIM21 may be part of the inflammasome scaffold. To ascertain that TRIM21 is a bona fide inflammasome component and interacts with ASC in intact macrophages, we generated *TRIM21* knock-out (*TRIM21*^KO^) THP-1 cells by targeting the exon 2 by CRISPR/Cas9 genomic editing and performed the sensitive proximity ligation assay (PLA). Specific PLA signals for TRIM21 and ASC were detected in untreated as well as primed and nigericin-activated Cas9-expressing control (Ctrl) THP-1 cells, but all PLA^+^ signals were completely lost in *TRIM21*^KO^ and *ASC*^KO^ THP-1 cells. These signals were highly specific, as omitting either the anti-TRIM21 or anti-ASC antibody prevented any PLA^+^ signal (Fig. 1b). Although, TRIM21 can function as an Fc receptor^11^, the interaction between ASC and TRIM21 did not involve Fc binding, as we also co-purified ASC by Ni^2+^ affinity purification with His-tagged TRIM21 after expression in HEK293 cells (Fig. 1c). Based on the interaction of TRIM21 and ASC and the known function of TRIM21 as an E3 ubiquitin ligase, we hypothesized that TRIM21 may ubiquitinate ASC. While not readily noticeable in total cell lysates, co-expression and affinity purification of His-tagged ubiquitin revealed the typical laddering and molecular weight shift of poly-ubiquitinated ASC only in the presence of TRIM21 (Fig. 1d). TRIM21 auto-ubiquitination is already visible in total cell lysates and the very weak ASC poly-ubiquitination observed in cells not transfected with TRIM21 is likely the result of endogenous TRIM21 present in HEK293 cells. Moreover, TRIM21 itself shows reduced poly-ubiquitination in the presence of ASC, implying that the ubiquitin chains are transferred onto ASC. We next applied this strategy to determine if the PYD or CARD are ubiquitinated by TRIM21 employing ASC^PYD^ and ASC^CARD^ expression and ASC^PYD^- and ASC^CARD^-specific antibodies. While the signal for ubiquitinated ASC^PYD^ was almost as intense as full-length ASC, the ubiquitinated ASC^CARD^ signal was significantly weaker than the full-length control, but there was still a size shift of ASC^CARD^ indicating ubiquitination (Fig. 1e). Ubiquitin contains 7 internal lysine residues, which are used for conjugation to target proteins, resulting in distinct functional consequences^18,19^. K48- and K63-mediated ubiquitination are common and have also been described for several inflammasome components^20,21^. We therefore first determined whether K48- or K63-linked ubiquitination is conjugated by TRIM21 onto ASC. We co-expressed ASC, TRIM21, and either ubiquitin, a ubiquitin mutant with all 7 lysine residues mutated to arginine (Ub^KO^), or a mutant with only K48 (Ub^KO−48K^) or K63 (Ub^KO−63K^) available for conjugation. Only co-expression of ASC with TRIM21 and ubiquitin resulted in ubiquitinated ASC, suggesting that TRIM21 did not transfer K48- or K63-linked ubiquitin chains to ASC (Fig. 1f). We therefore expanded this analysis to all 7 lysine residues. Only ubiquitin with intact K27 and K29 resulted in partial ASC ubiquitination (Fig. 1g), indicating that TRIM21-mediated K27 and K29 ubiquitination of ASC occur, but that TRIM21 may either cause more than one type of ubiquitination or even heterotypic, branched, or mixed ubiquitin chains. To confirm TRIM21-mediated ubiquitination of endogenous ASC, we transfected human primary macrophages with a non-targeting control or TRIM21 siRNA. ASC ubiquitination was strongly reduced upon TRIM21 siRNA transfection, and we also observed an LPS priming-dependent increase in ASC poly-ubiquitination (Fig. 1h). These results demonstrate that TRIM21 interacts with and ubiquitinates the inflammasome adaptor ASC.

### TRIM21 is necessary for inflammasome activation

**Fig. 2:**
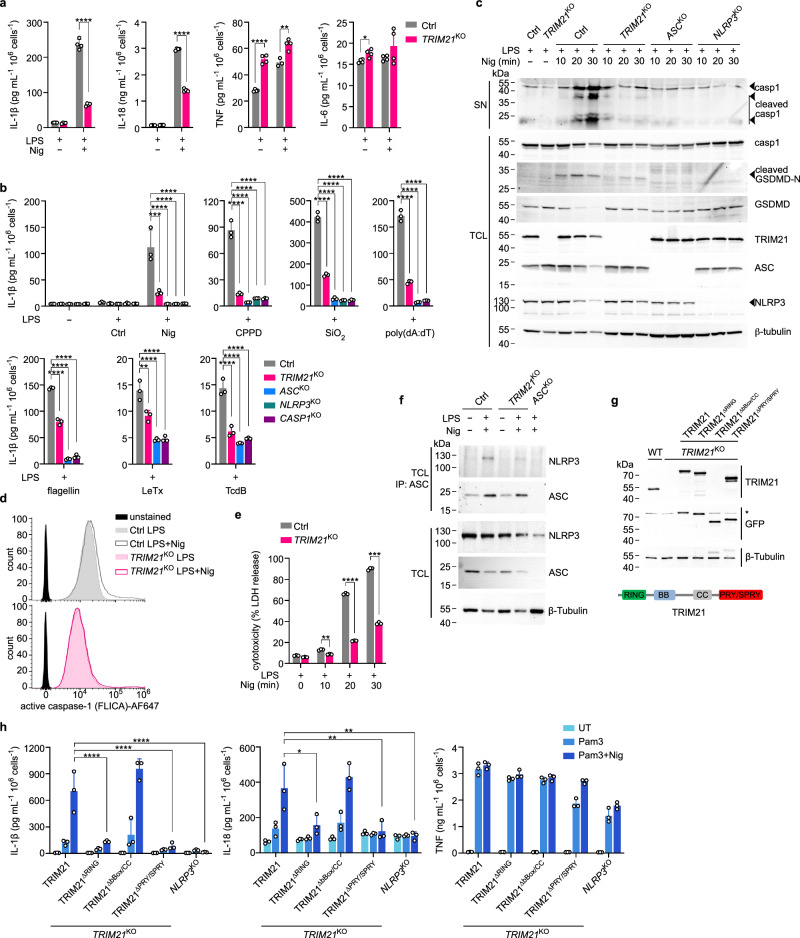
TRIM21 is required for inflammasome activation. **a** IL-1β, IL-18, TNF, and IL-6 ELISA of cleared culture supernatant (SN) from control (Ctrl) and *TRIM21*^KO^ THP-1 cells primed (LPS, 1 μg mL^−1^, 4 h) or primed and activated with nigericin (Nig, 10 μM, 30 min). (*n* = 3 biological replicates, mean ± s.d., parametric two-tailed unpaired *t*-test). **b** IL-1β ELISA of cleared SN from indicated THP-1 cells primed (LPS, 1 μg mL^−1^, 4 h) or primed and activated with Nig (10 μM, 30 min), Calcium Pyrophosphate Dihydrate (CPPD) crystals (125 μg mL^−1^, 6 h), Silica (400 μg mL^−1^, 6 h), TcdB (2 μg mL^−1^, 6 h), or primed and transfected with poly(dA:dT) (2 μg mL^−1^, 16 h), flagellin (100 ng mL^−1^, 6 h) or lethal toxin (LeTx) (1 μg mL^−1^, 16 h). (*n* = 3 biological replicates, mean ± s.d., one-way ANOVA with Dunnett’s post-test). **c** Immunoblot of cleaved/total caspase-1 (casp1), cleaved/total gasdermin D (GSDMD), TRIM21, ASC, NLRP3, and β-tubulin from SN or total cell lysates (TCL) from indicated THP-1 cells primed (LPS, 1 μg mL^−1^, 4 h) or primed and activated (Nig, 10 μM, 10, 20, 30 min). **d** FLICA assay of active caspase-1 (AlexaFluor647) of Ctrl and *TRIM21*^KO^ THP-1 cells primed (LPS, 1 μg mL^−1^, 4 h) or primed and activated (Nig, 10 μM, 20 min). **e** LDH release from Ctrl and *TRIM21*^KO^ THP-1 cells primed (LPS, 1 μg mL^−1^, 4 h) or primed and activated (Nig, 10 μM, 10, 20, 30 min), presented as percent cytotoxicity vs maximum LDH release (*n* = 3 biological replicates, mean ± s.d., parametric two-tailed unpaired *t*-test). **f** Immunoprecipitation (IP) of ASC from Ctrl, *TRIM21*^KO^, and *ASC*^KO^ THP-1 cells untreated or primed (LPS, 1 μg mL^−1^, 4 h) and activated (Nig, 10 μM, 30 min) and analyzed by immunoblot alongside TCL for ASC, NLRP3, and β-tubulin. **g** Immunoblot for TRIM21, GFP and β-tubulin of Ctrl, *TRIM21*^KO^, and *TRIM21*^KO^ THP-1 cells restored with GFP-TRIM21, GFP-TRIM21^ΔRING^, GFP-TRIM21^ΔbBox/CC^ or GFP-TRIM21^ΔPRY/SPRY^. TRIM21^ΔbBox/CC^ is detected by anti-GFP and not by anti-TRIM21 antibody (*cross-reactive protein). **h** IL-1β, IL-18 and TNF ELISA of cleared culture SN from restored *TRIM21*^KO^ as in (**g**) and NLRP3^KO^ THP-1 cells that were untreated (UT), primed with Pam3CSK4 (Pam3, 1 μg mL^−1^, 4 h) or primed and activated (Nig, 10 μM, 30 min) (*n* = 3 biological replicates, mean ± s.d., one-way ANOVA with Dunnett’s post-test). Results representative of *n* = 3 experiments.

To further interrogate the role of TRIM21 in NLRP3 inflammasome activation, we utilized control and *TRIM21*^KO^ THP-1 cells and found that LPS-primed and nigericin-activated *TRIM21*^KO^ THP-1 cells released significantly less IL-1β and IL-18 compared to control cells. However, TNF and IL-6 release was slightly elevated in response to LPS priming (Fig. 2a), in agreement with TRIM21 negatively regulating the NF-κB pathway^22^. Quantitative RT-PCR of LPS-primed control and *TRIM21*^KO^ THP-1 cells also revealed enhanced expression of *TNF* and *IL1B* in the absence of TRIM21, which are known to be regulated by NF-κB. However, the expression of the inflammasome components *ASC*, *NLRP3* and *CASP1* or *IL18* was not impacted and confirmed that the loss of IL-1β and IL-18 release observed in *TRIM21*^KO^ cells was not the result of impaired cytokine transcription (Supplementary Fig. 1a). We further confirmed that the deletion of *TRIM21* did not impact protein expression of other essential inflammasome components and CRISPR/Cas9 genomic deletion of *ASC*, *NLRP3* or *CASP1* did not impact TRIM21 expression (Supplementary Fig. 1b). We independently validated these results by silencing *TRIM21* expression in THP-1 and primary human macrophages and induced NLRP3 inflammasome activation with ATP in LPS-primed cells. Reminiscent of *TRIM21*^KO^ cells, the release of IL-1β into the culture supernatant was also significantly reduced in cells with silenced *TRIM21* expression compared to control siRNA-transfected cells (Supplementary Fig. 1c). Since TRIM21 interacted with the central inflammasome adaptor ASC, NLRP3 inflammasome activation with soluble or particulate activators as well as other ASC-containing inflammasomes, should be impacted by the loss of *TRIM21*. Indeed, LPS-primed *TRIM21*^KO^ THP-1 cells failed to release IL-1β in the supernatant not only in response to nigericin, but also in response to the particulate NLRP3 activators calcium pyrophosphate dihydrate (CPPD) and silica (SiO_2_) crystals, in response to NLRC4 inflammasome activation with intracellular flagellin, NLRP1 inflammasome activation with intracellular lethal toxin (LeTx) delivered by the protective antigen, pyrin inflammasome activation upon RhoA inhibition with TcdB and AIM2 inflammasome activation with dsDNA (poly(dA:dT)), considering that dsDNA-mediated inflammasome activation in THP-1 cells, just like in bone-marrow derived macrophages (BMDM), is AIM2-dependent, while it is NLRP3-dependent in some human monocytes^23^ (Fig. 2b). We ensured the specificity of the inflammasome response using *ASC*^KO^, *NLRP3*^KO^ and *CASP1*^KO^ THP-1 cells. The inflammasome-mediated release of IL-1β and IL-18 requires the activation of caspase-1, which is released by pyroptosis from macrophages, and we therefore directly determined active caspase-1 by immunoblot of culture supernatants. Cleaved caspase-1 p20 and an intermediate processing fragment appear in a time-dependent manner in the culture supernatant of LPS-primed and nigericin-treated control THP-1 cells but are almost completely absent in *TRIM21*^KO^ THP-1 cells, which also phenocopied *ASC*^KO^ and *NLRP3*^KO^ THP-1 cells. Since GSDMD is cleaved by caspase-1, we also observed that the time-dependent cleavage into GSDMD-N was significantly diminished in *TRIM21*^KO^ compared to control THP-1 cells (Fig. 2c). Defective maturation of caspase-1 in *TRIM21*^KO^ THP-1 cells was also observed in intact cells by flow cytometry, upon staining for activated caspase-1 with fluorochrome-labeled inhibitor of caspases (FLICA) (Fig. 2d and Supplementary Fig. 1d). Consequently, *TRIM21*^KO^ THP-1 cells showed significantly reduced cell death, as measured by LDH release after nigericin treatment of LPS-primed cells (Fig. 2e). All these responses are downstream of NLRP3 inflammasome assembly and we therefore investigated whether ASC is correctly recruited to NLRP3 by immunoprecipitating ASC and immunoblotting for NLRP3. While ASC was efficiently recruited and in a complex with NLRP3 upon nigericin treatment of primed control cells, it was not efficiently recruited in *TRIM21*^KO^ THP-1 cells (Fig. 2f), indicating that inflammasome assembly was defective in the absence of TRIM21. These results revealed that TRIM21 was necessary for inflammasome assembly and consequently for caspase-1 activation, GSDMD cleavage, release of IL-1β as well as pyroptotic cell death. To determine whether TRIM21 can also promote inflammasome responses, we utilized HEK293N cells, which lack the core inflammasome components, and reconstituted them with pro-IL-1β, pro-caspase-1, ASC, and NLRP3, which resulted in IL-1β release. However, co-transfection of TRIM21 significantly enhanced IL-1β release (Supplementary Fig. 1e). To obtain insights into the TRIM21 domain(s) responsible for inflammasome activation, we restored *TRIM21*^KO^ THP-1 cells with EGFP-TRIM21, -TRIM21^ΔRING^, -TRIM21^ΔbBox/CC^, and -TRIM21^ΔPRY/SPRY^, matching expression levels of endogenous TRIM21. Since the TRIM21 antibody recognizes an epitope within the b-Box, it cannot detect TRIM21^ΔbBox/CC^ by Western blot, but its expression was verified with a GFP antibody (Fig. 2g). Nigericin treatment of Pam3CSK4-primed cells revealed that *TRIM21*^KO^ THP-1 cells restored with TRIM21^ΔRING^ or TRIM21^ΔPRY/SPRY^ were completely defective in IL-1β and IL-18 but not TNF release, phenocopying *NLRP3*^KO^ THP-1 cells. However, TRIM21^ΔbBox/CC^-expressing cells were indistinguishable from TRIM21-expressing cells (Fig. 2h), thereby demonstrating that the RING and PRY/SPRY domains are crucial for TRIM21 to promote inflammasome responses.

### TRIM21 is required for inflammasome activation in vivo

**Fig. 3:**
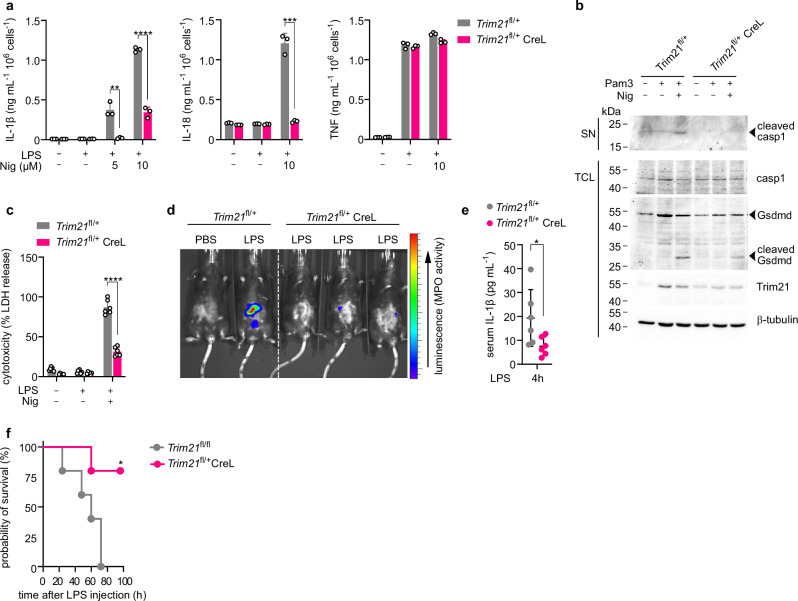
TRIM21 is required for inflammasome activation in vivo. **a** IL-1β, IL-18, and TNF ELISA of cleared culture supernatant (SN) from *Trim21*^fl/+^ and *Trim21*^fl/+^ CreL BMDM left untreated, primed with LPS (1 μg mL^−1^, 4 h), or primed and activated with nigericin (Nig, 5 or 10 μM as indicated, 30 min). (*n* = 3 biological replicates, mean ± s.d., parametric two-tailed unpaired *t*-test). **b** Immunoblot of cleaved and total caspase-1 (casp1), cleaved and total gasdermin D (GSDMD), TRIM21, and β-tubulin loading control from SN or total cell lysate (TCL) from *Trim21*^fl/+^ and *Trim21*^fl/+^ CreL BMDM left untreated, primed with Pam3CSK4 (Pam3, 1 μg mL^−1^, 4 h) or primed and activated with Nig (15 μM, 20 min). **c** LDH release from *Trim21*^fl/+^ and *Trim21*^fl/+^ CreL BMDM left untreated, primed with LPS (1 μg mL^−1^, 4 h) or primed and activated with Nig (15 μM, 20 min) is presented as percent cytotoxicity compared to maximum LDH release (*n* = 6 biological replicates, mean ± s.d., parametric two-tailed unpaired *t*-test). **d**, **e** In vivo imaging of myeloperoxidase (MPO) (**d**) and serum IL-1β ELISA (**e**) in *Trim21*^fl/+^ and *Trim21*^fl/+^ CreL mice i.p.-injected with LPS (2.5 mg kg^−1^, 4 h) (*n* = 4–6 mice, mean ± s.d., parametric two-tailed unpaired *t*-test). **f** Survival of male *Trim21*^fl/+^ and *Trim21*^fl/+^ CreL mice following i.p. injection of LPS (20 mg kg^−1^) presented as Kaplan–Meier estimate and Log-rank (Mantel–Cox) test (*n* = 5 mice). Results representative of *n* = 2 (**d–f**) or *n* = 3 (**a–c**) experiments.

To confirm that TRIM21-dependent inflammasome activation in macrophages has physiological relevance in vivo, we generated conditional *Trim21*^fl/fl^ mice, inserting loxP sites flanking exons 3–6 (Supplementary Fig. 2a). To delete *Trim21* specifically in myeloid cells, we crossed *Trim21*^fl/fl^ mice with lysozyme 2 (LysM)-Cre recombinase knock-in (CreL) mice. *Trim21*^fl/fl^CreL mice were not born, suggesting embryonic lethality, but *Trim21*^fl/+^CreL BMDM showed about 50% decreased *Trim21* expression by quantitative RT-PCR analysis. Similarly to THP-1 cells, reduced *Trim21* expression did not affect the expression of inflammasome components or their increased expression in response to LPS (Supplementary Fig. 2b). As we observed for human macrophages, *Trim21*^fl/+^CreL BMDM also released significantly less IL-1β and IL-18 compared to *Trim21*^fl/+^ BMDM, but TNF release was not affected (Fig. 3a). We also observed a comparable phenotype in BMDM isolated from conventional *Trim21*^–/–^ mice (Supplementary Fig. 2c) and in immortalized Cas9-expressing iBMDM upon knock-out of *Trim21* with *Trim21*-specific guide RNAs (gRNA) (Supplementary Fig. 2d, e). We confirmed reduced TRIM21 expression in total cell lysates of *Trim21*^fl/+^CreL BMDM, which coincided with reduced cleaved, active caspase-1 p20 in culture supernatants after Pam3CSK4 priming and nigericin treatment. In addition, cleavage of the caspase-1 substrate GSDMD was also reduced (Fig. 3b and Supplementary Fig. 2f). Accordingly, nigericin treatment of primed *Trim21*^fl/+^CreL BMDM resulted in significantly reduced pyroptosis compared to *Trim21*^fl/+^ BMDM, as measured by LDH release (Fig. 3c). Hence, TRIM21 has a conserved function in inflammasome activation in humans and mice. Defective NLRP3 inflammasome activation ameliorates LPS-induced release of IL-1β and lethality in mice^24^. Furthermore, macrophage-specific inflammasome inhibition also phenocopies global *Nlrp3*^–/–^^25,26^. Therefore, we intra-peritoneally injected a low dose of LPS into *Trim21*^fl/+^ and *Trim21*^fl/+^CreL mice and assessed neutrophil infiltration by in vivo imaging of myeloperoxidase^27^, which depends on NLRP3 inflammasome activation^28^. LPS-induced the influx of neutrophils into the peritoneal cavity in *Trim21*^fl/+^ mice but not in *Trim21*^fl/+^CreL mice (Fig. 3d). Concomitantly, IL-1β serum levels were significantly reduced in *Trim21*^fl/+^CreL mice (Fig. 3e). Significantly, intra-peritoneal injection of a lethal LPS dose resulted in lethality of *Trim21*^fl/+^ mice, but male (Fig. 3f) and female (Supplementary Fig. 2g, h) *Trim21*^fl/+^CreL mice were significantly protected from LPS-induced lethality, demonstrating that TRIM21 is essential for NLRP3 inflammasome responses in vivo.

### TRIM21 promotes ASC polymerization and release

**Fig. 4:**
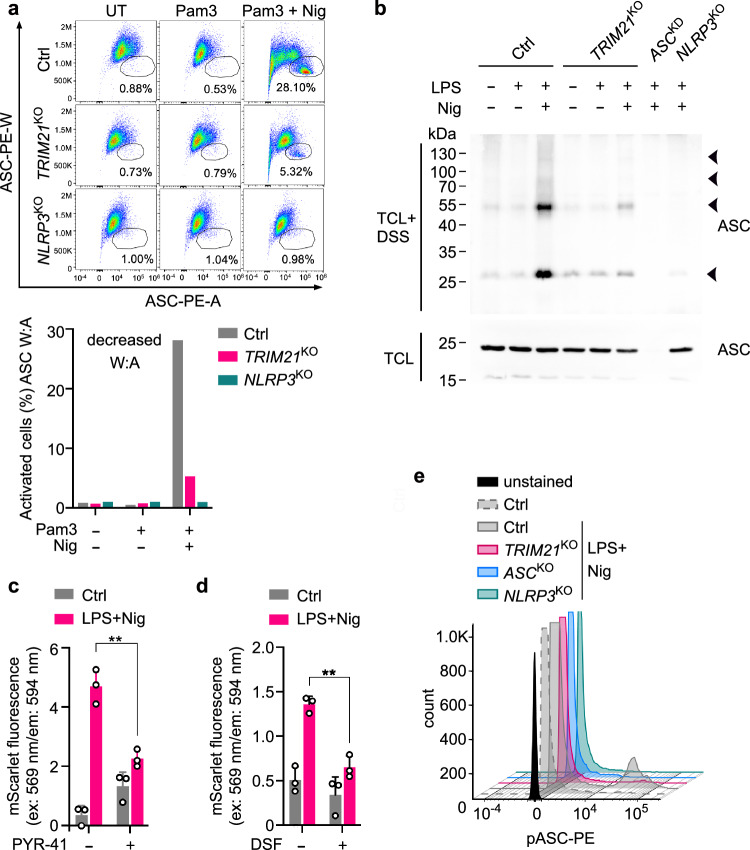
TRIM21 promotes ASC polymerization and release. **a** Flow cytometric detection of polymerized ASC in indicated THP-1 cells left untreated, primed with Pam3CSK4 (Pam3, 1 μg mL^−1^, 4 h) or primed and activated with nigericin (Nig, 10 μM, 30 min). Histograms represent ASC width(W):area(A) ratio. Percent of decreased W:A ASC signal represents activated cells, which is also presented as bar graph (bottom). **b** Immunoblot of ASC from total cell lysates (TCL) and DSS-crosslinked TCL from indicated THP-1 cells left untreated, primed with LPS (1 μg mL^−1^, 2.5 h) or primed and activated with Nig (10 μM, 15 min). **c**, **d** Fluorescence intensity of mScarlet-ASC in the cleared culture supernatant (SN) from mScarlet-ASC expressing THP-1 cells pre-treated with vehicle or with PYR-41 (25 μM, 4 h) (**c**) or DSF (40 μM, 1 h) (**d**), followed or not by LPS (0.5 μg mL^−1^, 2.5 h) priming and Nig (10 μM, 30 min) activation. (*n* = 3 biological replicates, mean ± s.d., parametric two-tailed unpaired *t*-test). **e** Flow cytometric detection of polymerized ASC particles released from untreated Ctrl THP-1 cells and Ctrl, *TRIM21*^KO^, *ASC*^KO^, and *NLRP3*^KO^ THP-1 cells primed with LPS (0.5 μg mL^−1^, 2 h) and activated with Nig (10 μM, 30 min) and presented as histograms. Unstained baseline is presented in black. Results representative of *n* = 3 experiments.

ASC polymerization into macromolecular fibrils or specks is a hallmark of inflammasome activation. ASC polymerization can be detected in intact cells by flow cytometry by the increased height:area and decreased width:area ratios of the ASC fluorescent signal^29^. Since TRIM21 ubiquitinates ASC, we investigated whether TRIM21 can impact this crucial step. Pam3CSK4-primed and nigericin-treated control displayed increased ASC polymerization into specks compared to untreated cells, based on the larger frequency of cells with decreased width:area ratio (Fig. 4a and Supplementary Fig. 3a) and an increased height:area ratio (Supplementary Fig. 3a, b) of ASC, while *NLRP3*^KO^ THP-1 cells largely lacked polymerized ASC specks and *TRIM21*^KO^ had reduced ASC specks compared to control cells by both analyses. We also observed impaired ASC polymerization by immunoblot of irreversibly crosslinked total cell lysates. Only LPS-primed and nigericin-treated control THP-1, but not *TRIM21*^KO^, *ASC*^KD^, and *NLRP3*^KO^ cells, displayed high molecular weight bands representing polymerized ASC (Fig. 4b) and hence, TRIM21 had an important role in ASC polymerization. Polymerized ASC particles are released into the extracellular space during pyroptosis, acting as a danger signal that propagates and perpetuates inflammasome responses^5,6^. We generated mScarlet-ASC-expressing THP-1 cells, allowing us to determine mScarlet-ASC release into supernatants by directly measuring fluorescence in cell-free culture supernatants. Accordingly, we detected mScarlet-ASC release after nigericin treatment of LPS-primed THP-1 cells. Moreover, pre-treatment with PYR-41, a specific inhibitor of ubiquitin-activating (E1) enzymes, which catalyze the first ubiquitination step, inhibited ASC release after LPS/nigericin-mediated inflammasome activation (Fig. 4c), confirming that ubiquitination is needed for ASC release. Treatment of cells with the GSDMD pore formation inhibitor disulfiram (DSF)^30^ also reduced the fluorescence signal in cell-free culture supernatants (Fig. 4d), indicating that it was mediated by pyroptosis. We also analyzed the release of endogenous polymerized ASC particles by flow cytometry by gating on small particles in culture supernatants of THP-1 cells. LPS-primed and nigericin-activated control THP-1 cells exhibited extracellular, polymerized ASC particles not present in untreated cells and also completely absent in *TRIM21*^KO^ THP-1 cells, similarly to *ASC*^KO^ and *NLRP3*^KO^ specificity controls (Fig. 4e and Supplementary Fig. 3c, d). Therefore, TRIM21 was required for ASC polymerization and the release of polymerized ASC particles.

### TRIM21 is co-released with polymerized ASC during inflammasome activation

**Fig. 5:**
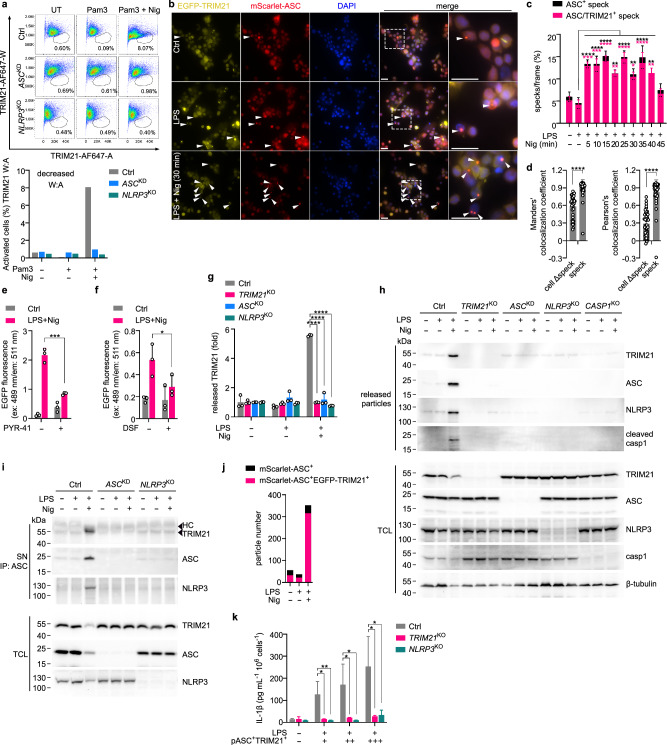
TRIM21 is co-released with polymerized ASC during inflammasome activation. **a** Flow cytometric detection of polymerized TRIM21 in indicated THP-1 cells untreated (UT), primed (Pam3CSK4, Pam3, 1 μg mL^−1^, 4 h), primed and activated (nigericin, Nig, 10 μM, 30 min). Percent of decreased width(W):area(A) TRIM21 signal ratio represents activated cells, also presented as histogram. **b**, **c** PMA-differentiated EGFP-TRIM21/mScarlet-ASC-expressing THP-1 cells untreated, primed (LPS, 0.5 μg mL^−1^, 2 h), primed and activated (Nig, 5 μM, 5–45 min). Representative microscopy images (30 min) (**b**) and quantification of ASC^+^ and ASC^+^TRIM21^+^ aggregates at all timepoints shown in Supplementary Fig. 5 (**c**) (*n* = 10 frames/sample, mean ± s.d., one-way ANOVA with Dunnett’s post-test). Nuclei/DNA were stained with DAPI. Scale bar: 10 μm. **d** Manders’ (**left**) and Pearson’s (**right**) co-localization coefficients calculated within the speck (speck) and cell area outside the speck (cellΔspeck) (*n* = 10 frames, mean ± s.d., parametric two-tailed unpaired *t*-test). **e**, **f** EGFP-TRIM21 fluorescence intensity in cleared supernatants (SN) from EGFP-TRIM21-expressing THP-1 cells pre-treated with vehicle, PYR-41 (25 μM, 4 h) (**e**) or DSF (40 μM, 1 h) (**f**) followed or not by LPS (0.5 μg mL^−1^, 2.5 h) priming and Nig (10 μM, 30 min) activation. (*n* = 3 biological replicates, mean ± s.d., parametric two-tailed unpaired *t*-test). **g** TRIM21 ELISA of cleared SN from indicated THP-1 cells untreated, primed (LPS, 1 μg mL^−1^, 2 h) or primed and activated (Nig, 10 μM, 45 min). (*n* = 3 biological replicates, mean ± s.d., one-way ANOVA with Dunnett’s post-test). **h** Immunoblot of TRIM21, ASC, NLRP3, total/cleaved caspase-1 (casp1), β-tubulin using ASC particles purified from SN alongside TCL from indicated THP-1 cells untreated, primed (LPS, 0.5 μg mL^−1^, 2 h), primed and activated (Nig, 10 μM, 30 min). **i** ASC immunoprecipitation (IP) from SN and immunoblot of TRIM21, ASC, NLRP3 alongside TCL from indicated THP-1 cells untreated, primed (LPS, 1 μg mL^−1^, 2 h), primed and activated (Nig, 10 μM, 30 min). HC heavy-chain. **j** Flow cytometric detection of polymerized mScarlet-ASC^+^ and mScarlet-ASC^+^EGFP-TRIM21^+^ particles released from mScarlet-ASC/EGFP-TRIM21-expressing THP-1 cells untreated, primed (LPS, 0.5 μg mL^−1^, 2 h), primed and activated (Nig, 10 μM, 30 min), presented as particle number. **k** IL-1β ELISA of cleared SN from Ctrl, *TRIM21*^KO^, *NLRP3*^KO^ THP-1 cells untreated, primed (LPS, 0.5 μg mL^−1^, 3 h) and activated with 2000(+)/5,000(++)/10,000(+++) sorted mScarlet-ASC^+^EGFP-TRIM21^+^ particles for 12 h (*n* = 3 biological replicates, mean ± s.d., one-way ANOVA with Dunnett’s post-test). Results representative of *n* = 3 experiments.

Since TRIM21 was interacting with ASC, we used the flow cytometric approach described above for ASC polymerization to analyze potential TRIM21 oligomerization in THP-1 cells. Pam3CSK4-primed and nigericin-treated control THP-1 cells, but not *ASC*^KD^ or *NLRP3*^KO^ THP-1 cells, displayed the decreased width:area ratio (Fig. 5a and Supplementary Fig. 4a) and the increased height:area ratio (Supplementary Fig. 4a, b) of TRIM21 signal compared to untreated or Pam3CSK4-primed cells, as observed for ASC. This indicated that TRIM21 polymerized into ASC-like aggregates in response to inflammasome activation. We therefore analyzed whether polymerized TRIM21 and ASC colocalized in THP-1 cells before being released, as we initially observed in HEK293 cells upon transient expression. To monitor ASC and TRIM21 subcellular localization during inflammasome activation, we utilized THP-1 monocytes stably expressing EGFP-TRIM21 and mScarlet-ASC. LPS-primed THP-1 cells were treated with nigericin in 5 min increments from 5 to 45 min and ASC and ASC/TRIM21 specks were quantified after fluorescence microscopy and image deconvolution (Fig. 5b, c and Supplementary Fig. 5). As expected, NLRP3 activation resulted in a time-dependent increase of intracellular ASC polymerization, until all ASC was eventually released from cells. TRIM21 also aggregated in a time-dependent manner after nigericin treatment and colocalized with polymerized ASC. Further, a magnified image of ASC^+^ and TRIM21^+^ aggregates, a tile view of all captured focus planes and the assembled 3D image (Supplementary Fig. 6a–c) show co-localization of ASC and TRIM21 in elongated polymers^2,3^. We further confirmed co-localization of ASC and TRIM21 by calculating Manders’ as well as Pearson’s co-localization coefficients, which demonstrated significantly increased co-localization in the speck compared to the cell area outside of the speck (cell Δspeck) (Fig. 5d). Because of this and the reduced TRIM21^+^ aggregates detected at later time points, we expected that, similarly to ASC, TRIM21 may also be released from inflammasome-activated cells. Using EGFP-TRIM21-expressing THP-1 cells, we confirmed the presence of EGFP-TRIM21 in cell-free culture supernatants of LPS-primed and nigericin-treated THP-1 cells, which was also prevented by pre-treatment with PYR-41 (Fig. 5e) or DSF (Fig. 5f), similarly to mScarlet-ASC. Hence, ASC and TRIM21 are both released in response to NLRP3 inflammasome activation, which requires ubiquitination and pyroptosis. We only detected TRIM21 in cell-free culture supernatants of LPS-primed and nigericin-treated control THP-1 cells, but not in *ASC*^KO^, *NLRP3*^KO^, or *TRIM21*^KO^ THP-1 cells by ELISA (Fig. 5g). Released, polymerized ASC particles also contain NLRP3 and caspase-1^5,6^. We therefore analyzed the composition of purified particles released into the cell-free culture supernatant of primed and nigericin-treated control and *TRIM21*^KO^ THP-1 cells by immunoblot, using *ASC*^KD^, *NLRP3*^KO^, and *CASP1*^KO^ cells as controls. We detected the inflammasome core components NLRP3, ASC, and caspase-1 in the particle fraction of cell-free culture supernatants of LPS-primed and nigericin-treated control THP-1 cells, but all three proteins were absent in the particle fraction purified from cell-free culture supernatants of *TRIM21*^KO^ THP-1 cells (Fig. 5h), indicating that TRIM21 was required for inflammasome particle release and was itself released after inflammasome activation. Since TRIM21 and ASC were released by a pyroptosis-dependent mechanism, we tested the possibility that TRIM21 was released as part of the polymerized ASC particle in response to inflammasome activation and immunoprecipitated ASC from culture supernatants of inflammasome-activated THP-1 cells. ASC was immunoprecipitated only in primed and nigericin-activated control THP-1 cells and was not present in *NLRP3*^KO^ and *ASC*^KD^ THP-1 cells. As previously described, NLRP3 co-immunoprecipitated with ASC^5^, but we also detected TRIM21 (Fig. 5i). Flow cytometric analysis of THP-1 cells with mScarlet-ASC and EGFP-TRIM21 expression further confirmed the presence of ASC^+^ particles only in LPS-primed and nigericin-treated culture supernatants and that most ASC^+^ particles were also TRIM21^+^ (Fig. 5j and Supplementary Fig. 7), demonstrating that ASC^+^TRIM21^*+*^ particles are released after inflammasome activation. ASC particles propagate and perpetuate inflammasome responses in bystander cells^5,6^ and purified ASC particles were able to induce IL-1β release in LPS-primed THP-1 cells. However, this response required NLRP3 and TRIM21 expression in recipient cells (Fig. 5k), indicating that TRIM21 was part of the polymerized and released ASC particles.

### TRIM21 deficiency alleviates autoinflammation in vivo

**Fig. 6:**
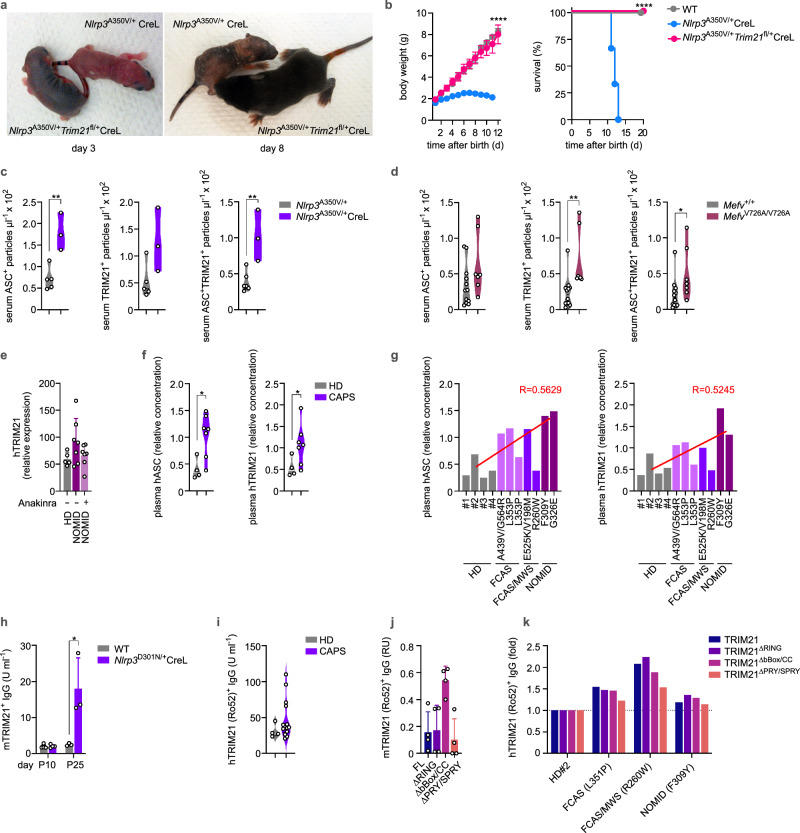
TRIM21 deficiency alleviates autoinflammation in vivo. **a** Representative pictures of *Nlrp3*^A350V/+^ CreL and *Nlrp3*^A350V/+^ *Trim21*^fl/+^ CreL pups at day 3 and day 8 after birth. **b** Body weight (**left**) and survival analysis (**right**) of wild-type (WT), *Nlrp3*^A350V/+^ CreL and *Nlrp3*^A350V/+^ *Trim21*^fl/+^ CreL pups (*n* = 6−11 pups, linear regression analysis [left] and Log-rank (Mantel–Cox) test [right]). **c**, **d** Flow cytometric detection and quantification of polymerized ASC^+^, TRIM21^+^ and ASC^+^TRIM21^+^ particles in the serum of *Nlrp3*^A350V/+^ and *Nlrp3*^A350V/+^ CreL pups at day 17 (**c**) and *Mefv*^*+/+*^ and *Mefv*^V726A/V726A^ mice at day 30–90 (**d**). (**c**: *n* = 6–11 pups; **d**: *n* = 7–12 pups, parametric two-tailed unpaired *t*-test). **e** Relative mRNA expression of *TRIM21* as determined by RNAseq of total human blood cells (GEO accession GSE57253)^36^ in healthy donors (HD) and Neonatal Onset Multisystem Inflammatory Disease (NOMID) patients before and after treatment with Anakinra (*n* = 5–7 patients, mean ± s.d.). **f**, **g** Plasma from HD and patients with active flares of Familial Cold Autoinflammatory Syndrome (FCAS), and/or Muckle–Wells Syndrome (MWS), or NOMID was analyzed for ASC and TRIM21 by ELISA and presented as HD versus CAPS (**f**) and each patient sample and corresponding mutation grouped by disease (**g**). (*n* = 11 human plasma samples, **f** parametric two-tailed unpaired *t*-test; **g** linear regression analysis of ASC and TRIM21 present in HD, FCAS, MWS, and NOMID). **h**, **i** Sera from WT and *NLRP3*^D301N/+^CreL mice at day 10 and 25 after birth (**h**) and plasma from HD and CAPS patients (**i**) were analyzed for TRIM21 IgG by ELISA. (**h**: *n* = 3–4 pups, parametric two-tailed unpaired *t*-test, mean ± s.d.), (**i**: *n* = 18 human plasma samples). **j** Sera from *NLRP3*^D301N/+^CreL mice were analyzed for TRIM21 IgG by ELISA using full-length (FL) TRIM21 and domain-deleted TRIM21 as antigen and presented as relative units (RU) (*n* = 4 pups, mean ± s.d.). **k** Plasma samples from HD and CAPS patients were analyzed for TRIM21 IgG by ELISA using full-length TRIM21 and domain-deleted TRIM21 as antigen and presented as fold increase compared to HD. Results are representative of *n* = 2 experiment(s).

CAPS is a spectrum of autoinflammatory diseases, including familial cold autoinflammatory syndrome (FCAS), Muckle–Wells syndrome (MWS), and neonatal onset multisystem inflammatory disease (NOMID), which are caused by hereditary mutations and somatic mosaicism of NLRP3^31,32^. The human CAPS phenotype can be recapitulated in mice by knock-in of CAPS-associated NLRP3 mutations^33,34^. We specifically utilized mice expressing a floxed *Nlrp3*^A350V^ MWS allele, corresponding to human *NLRP3*^A352V^, and CreL expression, which leads to NLRP3 inflammasome activation in myeloid cells in vivo^33^. *Nlrp3*^A350V/+^CreL mice developed systemic inflammation and displayed the characteristic inflammatory skin abscesses and lesions shortly after birth, which developed into scaling erythema^33^. However, *Nlrp3*^A350V/+^*Trim21*^fl/+^CreL mice completely lacked this inflammatory skin phenotype (Fig. 6a). *Nlrp3*^A350V/+^CreL mice also displayed stunted growth and lethality within 2 weeks after birth, while *Nlrp3*^A350V/+^*Trim21*^fl/+^CreL mice developed normally and were indistinguishable from wild-type mice (Fig. 6b). Extracellular polymerized ASC particles have been identified in serum and other bodily fluids of patients and mice with autoinflammatory disease^5,6^. Since we detected ASC^+^TRIM21^+^ particles in culture supernatants of pyroptotic macrophages after NLRP3 activation, we tested serum from *Nlrp3*^A350V/+^ and *Nlrp3*^A350V/+^CreL mice by flow cytometry. *Nlrp3*^A350V/+^CreL mice not only revealed increased levels of ASC^+^ particles, but also the presence of TRIM21^+^ particles, and that almost all ASC^+^ particles were also TRIM21^+^ (Fig. 6c and Supplementary Fig. 8). The presence of TRIM21^+^ particles was not limited to *Nlrp3*^A350V/+^CreL mice, as we also detected the presence of ASC^+^ as well as TRIM21^+^ particles in the serum of *Mefv*^V726A^ mice. This mutated pyrin is responsible for constitutive pyrin inflammasome activation and causes FMF^35^. Most ASC^+^ particles from *Mefv*^V726A^ mouse serum were also TRIM21^+^ (Fig. 6d and Supplementary Fig. 8). To correlate our findings with human disease, we analyzed GEO accession GSE57253, which consists of mRNAseq results from whole blood cells obtained from five healthy pediatric controls and seven patients with NOMID with active disease before anakinra treatment and the same seven patients with NOMID with inactive disease after anakinra treatment^36^. Interestingly, we noticed a trend for elevated TRIM21 mRNA expression in whole blood of our small NOMID patient cohort with active disease compared to healthy donors, which was however, reduced in patients with inactive disease after treatment with the IL-1β blocking drug anakinra (Fig. 6e). Hence, there could be a link between TRIM21 expression and human CAPS but further verification is required due to the small cohort. Importantly, we also discovered significantly elevated levels of TRIM21 in the plasma of CAPS patients compared to healthy donors, similar to ASC (Fig. 6f). The severity spectrum of CAPS ranges from FCAS, MWS to NOMID with some overlap, and we observed a disease severity-dependent increase of plasma ASC and TRIM21 with a comparable pattern (Fig. 6g). Anti-ASC autoantibodies have also been identified in several autoinflammatory diseases and may further propagate inflammation by opsonization of polymerized ASC particles^5,6^. On the other hand, anti-TRIM21 autoantibodies (anti-Ro52) are commonly present in autoimmune connective tissue disorders, where they have a high clinical diagnostic value^9^ but have not been identified in autoinflammatory disease. Since we identified systemic TRIM21^+^ particles, we analyzed CAPS plasma for the presence of anti-TRIM21 autoantibodies. The severity of different CAPS diseases is inverse in mice compared to humans, and MWS mice have a short life span^33^. We thus analyzed serum from myeloid-specific NOMID (D301N) KI mice (*Nlrp3*^D301N/+^CreL), which have mild disease. Congruent with the newly identified systemic TRIM21^+^ particles, we detected anti-TRIM21 (Ro52) IgG in the serum of *Nlrp3*^D301N/+^CreL mice 25 days after birth (Fig. 6h). Importantly, we also observed anti-TRIM21 (Ro52) IgG in the plasma of some CAPS patients by ELISA (Fig. 6i). Most autoantibodies in systemic autoimmune rheumatic diseases target the coiled coil region and rarely also the RING domain^37^. Using full-length and domain-deleted mouse and human TRIM21 as antigens purified from transiently transfected HEK293 cells or from stably expressing THP-1 cells, respectively, we observed that the anti-TRIM21 autoantibodies in CAPS mice (Fig. 6j) and patients (Fig. 6k) target all domains. These data revealed that TRIM21 and anti-TRIM21 autoantibodies are present in autoinflammatory disease patients and mice in addition to the previously described systemic ASC.

## Discussion

Assembly of the inflammasome scaffold facilitates the nucleated polymerization of the adaptor ASC into a filamentous, spherical structure and subsequent polymerization and induced-proximity activation of caspase-1 by increasing the local concentration of inflammasome components. The prion-like self-propagating polymerization of ASC acts as a point of no return^2,3^. Therefore, inflammasome assembly requires a tight control and is governed by a complex regulatory network, including multiple steps of post-translational modifications, such as ubiquitination of NLRP3 and other inflammasome sensors, as well as ASC, and caspase-1, which positively and negatively regulates inflammasome activation^1,21^. Because polymerization of ASC is the common and central step downstream of the activation of distinct inflammasome sensors, we focused our study on proteins that may regulate this step by purification of ASC-binding proteins, which revealed TRIM21 as a previously unknown ASC-interacting protein. Since TRIM21 can function as a high-affinity receptor for the immunoglobulin Fc domain^11^, anti-ASC antibodies used for ASC labeling and isolation could be captured non-specifically by TRIM21 to facilitate an indirect interaction. However, we eliminated this possibility by using Ni^2+^ affinity purification of His-tagged TRIM21 as well as co-localization of EGFP-TRIM21 and mScarlet-ASC fusion proteins. In addition, we find that TRIM21 ubiquitinates ASC. Preliminary analysis based on ubiquitin mutants with only one of its seven lysine residues available for conjugation revealed K27- and K29-linked ASC ubiquitination. However, the ubiquitination pattern with ubiquitin-K27 or ubiquitin-K29 alone appears partial and distinct from wild-type ubiquitin. This could be a result of different types of conjugation to the two distinct sites identified within the PYD and the CARD, or may suggest possible heterotypic, branched, or mixed ubiquitin chains, and future studies are necessary to define these modifications in detail^38^. There is limited information on the functional consequences of these types of ubiquitination, but effects on autophagy, type I interferon response, and autoimmune disease have been reported^39–41^. For example, RNF34 mediates K27/K29 ubiquitination of MAVS, which is like ASC a central adaptor for intracellular PRRs^42^. In addition, other TRIM members, including TRIM14 and TRIM24, have recently been identified to conjugate K27 and/or K29 Ubiquitin^41,43^. Protein ubiquitination can be reversed by deubiquitinases. The ubiquitin-specific protease (USP) 7 regulates NLRP3 inflammasome activation and has been reported to collaborate with TRIM21 in SNAI2 homeostasis and p53 stability^44–46^. However, any role in the deubiquitination of TRIM21-mediated ASC ubiquitination is unknown and will require future studies. However, other enzymes can also ubiquitinate ASC^21^. TRAF6-mediated K63-linked ASC ubiquitination results in degradation of ASC by autophagy and K63-linked deubiquitination of ASC by USP50 promotes NLRP3 inflammasome activation^47,48^. On the other hand, virus-induced TRAF3-mediated K63-linked ubiquitination of ASC by TRAF3 and MAVS promotes ASC polymerization and IL-1β release^49,50^. ASC is also ubiquitinated by Peli1 on K55 and *Peli1*^–/–^ BMDM show reduced ASC polymerization and release of IL-1β ^51^. In addition, conjugation of linear ubiquitin chains to ASC enhances NLRP3 inflammasome assembly^52^. Other TRIM family members also participate in inflammasome activation but regulate NLRP3 protein levels by ubiquitination. TRIM24, TRIM31, and TRIM40 cause proteasomal degradation, while TRIM62 stabilizes NLRP3, ASC and caspase-1^53–56^. However, in contrast to the previously identified ubiquitin modifications, we showed that genetic deletion of *TRIM21* almost completely impaired ASC polymerization, caspase-1 activation, pyroptosis, and release of IL-1β and IL-18, as well as polymerized ASC particles. We observed a minor background of polymerized ASC in *TRIM21*-deficient human and mouse macrophages, which could be due to our approach and quantification. However, we cannot exclude that other mechanisms allow some cells to overcome this defect. This inflammasome response required the RING domain of TRIM21, which harbors the catalytic domain, as well as the PRY/SPRY domain, which determines substrate specificity as well as immunoglobulin Fc binding^16,57^, without having consequences on ASC stability, suggesting that TRIM21 is responsible for a crucial step without impact on protein stability of ASC. Indeed, we identified TRIM21 interaction with ASC before inflammasome activation as well as on the released polymerized ASC particles, suggesting that TRIM21 interacted with ASC not just temporarily for ubiquitination, but that it remained a structural component of the polymerized ASC particle, further emphasizing the crucial role of TRIM21 for the function of ASC. Reminiscent of the TRIM21-mediated ASC polymerization, K27 and K29 ubiquitination of LRRK2 has been shown to promote its aggregation^58^.

TRIM21 has been proposed to function analogous to bacterial superantigens through an antibody bipolar bridging mechanism, resulting in pathological accumulation of anti-TRIM21 autoantibody-immune complexes^59^. In addition, anti-TRIM21 autoantibodies (anti-Ro52) are a common characteristic in several systemic autoimmune rheumatic diseases, including Systemic Lupus Erythematosus, Sjögren’s syndrome, and myopathies and can be detected with frequencies of up to 95% in patients. Some correlations have been detected between *TRIM21* allelic polymorphisms and autoantibody production^60^. Significantly, determining the anti-TRIM21 autoantibody titer is routinely used for the diagnosis and prognosis of both organ-specific and systemic autoimmune diseases, but they have not been linked to autoinflammatory disease^9,15^. We now find anti-TRIM21 autoantibodies in CAPS autoinflammation, but in contrast to autoantibodies primarily targeting the coiled coil domain in autoimmune diseases^37^, we observed targeting of all TRIM21 domains.

There are some important unanswered questions about anti-TRIM21 autoantibodies. For instance, how does the usually nuclear and cytosolic TRIM21 become a key autoantigen, and how and why do these autoantibodies reach the nuclear and cytosolic TRIM21 and contribute to disease pathogenesis^16,61^? Our study possibly provides an explanation, since we showed that upon inflammasome activation, TRIM21 is released from pyroptotic cells as part of the polymerized ASC particle, thereby providing immune cells access to possibly allow recognition and targeting of ASC/TRIM21 particles as autoantigen. Significantly, we detected anti-TRIM21 autoantibodies in the serum of CAPS mice and in the plasma of human CAPS patients. TRIM21 autoantibodies may serve a role beyond a biomarker by potentially opsonizing polymerized ASC^+^TRIM21^+^ particles, which may play a role in enhancing their phagocytosis by bystander cells to propagate and perpetuate inflammasome responses^5,6^. Since endogenous ASC is incorporated into the internalized polymerized ASC particle^5^, the presence of TRIM21 may also directly enhance ASC polymerization by ubiquitinating endogenous ASC in bystander cells, and we showed that TRIM21 is also necessary for the ASC particle-induced inflammasome response in recipient cells. Furthermore, TRIM21 may retain catalytic activity in the circulation once released by pyroptosis. Active caspase-1 is also released by pyroptosis and has been suggested to be catalytically active and can proteolytically cleave extracellular pro-IL-1β of dying cells^5^.

TRIM21 has been recently shown to bind to GSDMD via its PRY/SPRY to promote GSDMD expression and to subsequently facilitate GSDMD-N oligomerization. This activity is independent of its E3 ligase activity and the RING domain of TRIM21^17^. Similarly, over-expression of TRIM21 in B cells results in RING domain-dependent apoptotic cell death^10^. This contrasts with our biochemical and genetic results that showed that the TRIM21 RING and PRY/SPRY domains are necessary for inflammasome-mediated IL-1β release. Furthermore, we did not observe significantly different GSDMD expression upon *TRIM21* knock-out in human or mouse macrophages, but identified that TRIM21 is necessary for ASC recruitment to NLRP3, ASC polymerization, and caspase-1 activation, which are upstream of GSDMD cleavage and pore formation. We also provide evidence that TRIM21 and ASC can already interact in naïve cells and TRIM21-mediated ASC ubiquitination could be necessary for the recruitment to upstream PYD-containing PRRs such as NLRP3, which enables nucleation of ASC polymerization and inflammasome responses. However, a fundamental aspect of ubiquitination is the existence of multiple targets for each E3 ubiquitin ligase, as well as multiple E3 ubiquitin ligases targeting the same substrate, resulting in redundancy, flexibility, and conjugation of different ubiquitin chains, which yields unique mechanisms for each specific substrate^62^.

Inconsistent results have been reported for the phenotype of global *Trim21*^–/–^ mice. In agreement with negative regulation of NF-κB and type I IFN, *Trim21*^–/–^ mice developed a systemic autoimmune phenotype caused by increased IRF3/5/8 transcription factor activity and expression of Th17 pathway cytokines, including IL-6, IL-12/IL-23p40, and IL-17 as well as IFN-β in effector T cells of the draining lymph node and spleen and macrophages^63^. These mice were also born at a reduced Mendelian ratio. Other studies, however, observed that TRIM21 expression in macrophages causes IRF3 and IRF8 ubiquitination and enhanced activity, resulting in Toll-like receptor (TLR) -induced IFN-β and IL-12p40 expression^12,64^. In contrast, another study in these *Trim21*^–/–^ mice observed that TRIM21 ubiquitinates and decreases IRF7 expression in line with earlier identified TRIM21-mediated degradation of IRF3, and that mice showed reduced type I IFN expression in response to TLR agonists^13,65^. On the other hand, independently-generated *Trim21*^–/–^ mice revealed impaired ubiquitination of IRF3/8, induction of NF-κB, and upregulation of pro-inflammatory cytokines, including IL-1β, IL-6, and CXCL10 in MEFs, but no impact in immune cells without any inflammatory or autoimmune phenotype in vivo. However, numerous other TRIM family members were also upregulated and it is possible that functionally redundant TRIM family proteins can compensate for the loss of *Trim21*^22^. Another study using MEFs from these *Trim21*^–/–^ mice showed enhanced NF-κB and IRF3/5/7, but not IRF8 activation in response to antibody-bound virus internalization in MEFs and consequently, reduced release of IL-6, TNF, and IL-12 in macrophages^66^. This TRIM21-mediated response of antibody-bound DNA or RNA viruses engages the cytosolic nucleic acid sensors cGAS and RIG-I, respectively^67^. These differences were attributed to differences in deletion strategies and mouse strains, deleting exons 3–5 or 5–8, using C57BL/6 and BALB/c ES cells, respectively and either deleting or retaining the PGK-neo cassette in addition to housing differences^22,63,68^. Considering the potential functional compensation and disparate phenotypes in existing *Trim21*^–/–^ mice, we generated conditional *Trim21*^fl^ mice to analyze inflammasome responses in macrophages. We floxed exons 4–8 and did not obtain homozygous *Trim21*^fl/fl^CreL mice and therefore performed our studies in *Trim21*^fl/+^CreL mice. We also observed a comparable defect in inflammasome-mediated IL-1β release in independently generated *Trim21*^–/–^ iBMDM and THP-1 cells and in human macrophages with siRNA-mediated reduction of TRIM21 expression of about 50%. This lethality could be a consequence from TRIM21 targeting key developmental pathways, since TRIM21 ubiquitination regulates Hippo/YAP inactivation, which is crucial for regulating organ size during embryonic development^69^. Moreover, embryonic lethality has also been observed for the essential NLRP3 inflammasome component NEK7^70^.

Our study identified the involvement of TRIM21 in the regulation of inflammasome assembly, ASC polymerization, and the release of ASC/TRIM21 particles. Accordingly, we detected systemic ASC/TRIM21 particles in mice and human patients with autoinflammatory disease as well as TRIM21 autoantibodies, known and used as diagnostic biomarker in autoimmune disease. Our work also links autoinflammatory and autoimmune responses, which may have important clinical ramifications for both disorders. Our findings could also explain the elevated inflammasome activity in autoimmune disease and, in a wider context, in other chronic low-grade inflammatory diseases. Hence, circulating ASC/TRIM21 particles and TRIM21 autoantibodies may be used as diagnostic and prognostic tools for autoinflammatory disorders. Future studies, however, will still need to determine whether ASC/TRIM21 particles are also present in autoimmune diseases. Expression of TRIM21 is induced by interferons, which can be regulated by IL-1β/IL-1R^71,72^, in agreement with our data showing that the IL-1R antagonist anakinra reduces TRIM21 expression. Consequently, future studies should investigate the role of TRIM21 itself in such a regulatory loop, as well as identify the identity of the specific deubiquitinases regulating this response.

Overall, our novel discovery that TRIM21 plays an essential role in the regulation of ASC polymerization during inflammasome activation provides important novel insights into the still incompletely understood mechanism of inflammasome assembly and the crucial aspect of inflammasome response propagation to bystander cells. Our study further linked one of the best described autoantibodies to the pathology of autoinflammatory disease in humans and in mice, which may have important clinical ramifications.

## Methods

This research complies with all relevant ethical regulations and has been approved by the Institutional Animal Care and Use Committees (IACUC) at Cedars-Sinai Medical Center and University of California San Diego and adheres to the NIH Guide for the Care and Use of Laboratory Animals, the Institutional Review Board at Cedars-Sinai Medical Center, University of California San Diego and Rady Children’s Hospital-San Diego, as well as the Institutional Biosafety Committee (IBC) at Cedars-Sinai Medical Center. Investigators were blinded for in vivo experiments, as mice were only marked by numbers, while investigators were not blinded for cell-based studies and human subjects, the latter being labelled by their genotype. Non-blinding had no impact on any results, as quantitative data were obtained and no values were excluded. Sample sizes were determined based on prior experience with comparable studies or were determined by the number of available human subjects. Participants were not randomly assigned to this study, as inclusion was determined by their NLRP3 genotype, but we randomly assigned animals and cells to different treatment groups.

### Mice

*Trim21*^*fl/+*^ mice were generated by Ingenious Targeting Laboratory. A 13.58 Kb region containing *Trim21* was subcloned from a positively identified C57BL/6 BAC clone (RP23: 191P13) into pSP72 (Promega, P2191). The short homology arm extended 2.48 Kb 5’ to exon 5. The long homology arm ended 3’ to exon 8 and was 6.25 Kb in length. A pGK-gb2 loxP/FRT flanked Neo cassette was inserted 536 bp upstream of exon 5. The single loxP site, containing engineered Bgl II, Xba I, HpaI, and EcoRI sites for Southern Blot analysis, was inserted 215 bp downstream of exon 8. The entire targeted region was 4.85 Kb and includes exons 5–8. The total size of the targeting construct was 17.68 Kb. Ten μg of the targeting vector was linearized by NotI, electroporated into BA1 (C57BL/6 × 129/SvEv hybrid) ES cells and G418 selected. The targeting vector was confirmed by PCR and sequencing using the primers P6, T7, N1, N2, loxP, A2, UNI, SDL2, and RIPA8. Selected ES cell clones were confirmed by PCR and DNA sequencing for short arm integration and clones were verified by Southern Blotting analysis using an internal short arm probe (PB7/8) targeted against the 5’ internal region, following digestion with BgIII. Clones were also confirmed for long arm integration following digestion with BgIII using the PB1/2 probe and an internal long arm probe (PB5/6) after XbaI digestion. Correctly targeted clones were injected, and mice were backcrossed 10 generations. Lysozyme 2 (LysM)-Cre recombinase knock-in (CreL) mice (B6.129P2-Lyz2^tm1(cre)Ifo^/J; strain# 004781), C57BL/6J mice (strain# 000664), and C57BL/6-*Trim21*^tm1Hm^/J (strain# 010724) were obtained from The Jackson Laboratory. *Nlrp3*^A350V^, *Nlrp3*^D301N^, *Mefv*^V726A^, *Nlrp3*^–/–^, and *Trim21*^–/–^ mice were described earlier^22,24,33,35^. The primers used for genotyping are indicated in Supplementary Table 1. Mice were housed in specific pathogen-free (SPF) conditions, bred in house, group housed and allowed water and normal diet chow *ad libitum*. Experimental and control animals were co-housed. Light on/off cycles were controlled in a 12 h/12 h shift, while the temperature of the animal room was kept steady at 22 ± 2 °C. All experiments used age- and sex-matched, randomly assigned mice 12–16 weeks of age and mice were euthanized with carbon dioxide. All animal studies were conducted at Cedars-Sinai Medical Center and University of California San Diego according to procedures approved by the Institutional Animal Care and Use Committees (IACUC) and adherence to the NIH Guide for the Care and Use of Laboratory Animals.

### LPS-induced peritonitis

For LPS-induced peritonitis, 10–12 week-old male mice had their abdomen shaved under anesthesia and were randomly selected for i.p. injection with PBS (Corning, 21-031-CV) or with LPS (2.5 mg kg^−1^, *E. coli* Serotype O111:B4, Sigma-Aldrich, L2630-100MG). 3 h later, mice were i.p. injected with 200 mg kg^−1^ of XenoLight Rediject inflammation probe (PerkinElmer, 760535). Mice were transferred into an anesthesia chamber and in vivo bioluminescence was captured by imaging (IVIS Spectrum, PerkinElmer) 10 min post-injection with a 5 min exposure on anesthetized mice. Images were quantified with Living Image software (PerkinElmer)^73^. 4 h post-LPS injection, mice were euthanized, and serum levels of IL-1β were measured by ELISA (Invitrogen, 88-7013-22). Endotoxic shock was induced in 10–12 week-old male and female mice by i.p. injection of a lethal dose of 20 mg kg^−1^ LPS (*E. coli* Serotype O111:B4) and mice were monitored four times daily for survival.

### Cells

Authenticated THP-1 cells (ATCC, clone TIB-202) were maintained in RPMI 1640 (Cytiva, SH30027FS) supplemented with 10% heat-inactivated FBS (Gibco, 16140071), 25 mM HEPES buffer (Corning, 25-060-Cl), and 1% penicillin and streptomycin (Gibco, 15140122). THP-1 cells originate from the peripheral blood of a one-year-old male patient with acute monocytic leukemia. Cells were used at low passage and routinely screened for mycoplasma infections (MycoStrip, Invivogen, rep-mysnc-100). Primary human peripheral blood mononuclear cells were isolated by counter-current centrifugation in the presence of 10 μg mL^−1^ polymyxin B (Gibco, 15140122) with a JE-6B rotor (Beckman Coulter) from de-identified healthy donor buffy coats (Red Cross) after obtaining informed consent under a protocol approved by Cedars-Sinai Institutional Review Board, as described in ref. ^74^. *NLRP3*^KO^, *CASP1*^KO^, and *ASC*^KD^ THP-1 cells were described earlier^75–77^. *ASC*^KO^ THP-1 cells were obtained from Invivogen (#thp-koascz) and maintained according to the manufacturer’s instructions. Mouse BMDM were isolated by flushing cells from femurs of 10–12 week-old males or females and differentiated for 6 days in DMEM high glucose (Corning, 10-013-CV) supplemented with 10% FBS, 1 mM HEPES, and 1% penicillin and streptomycin in the presence of 20 ng mL^−1^ M-CSF (Gibco, AF-315-02-10UG). WT BMDM were immortalized by infection with the raf/myc-encoding J2 retrovirus^78^. Briefly, J2-containing culture supernatant was collected from AMJ2-C11 cells (ATCC, CRL-2456), 0.45 µm filtered and used to infect BMDM at day 7 after isolation in the presence of polybrene (3 µg mL^−1^, Selleck Chemical, 50-313-4437) and HEPES (5 mM) at 32 °C for 12–16 h.

### Generation of *TRIM21*^KO^ cells

*TRIM21*^KO^ THP-1 cells were generated using CRISPR/Cas9 targeting^79^. Two gRNAs were designed to target hTRIM21 Exon 2: gRNA#1: ATGCTCACAGGCTCCACGAA (Exon2 ORF bp67-86); gRNA#2: ATGTTGGCTAGCTGTCGATT (Exon2 ORF bp196-215) and cloned into lentiCRISPRv1 (Addgene, plasmid 49535). After lentiviral transduction of Cas9-expressing THP-1 cells using lentiCRISPRv1 as control as described below, clonal populations were screened by PCR amplification and sequencing of the targeted genomic sequence using hTRIM21gRNA1-F and hTRIM21gRNA2-R primers. *Trim21*^KO^ iBMDM were generated using CRISPR/Cas9 targeting. Two gRNAs were designed to target msTrim21 Exon 3: gRNA#1: TGGCCACATTCGATACTCAT (Exon 3 ORF bp67-86); gRNA#2 GTCTATTGGGCCTGAGGTTT (Exon 3 ORF bp171-190) and transfected into WT iBMDM stably expressing Cas9-GFP using Lipofectamine RNAi/MAX (Invitrogen, 13778150). After transfection, clonal populations were screened by PCR amplification and sequence verified using msTRIM21gRNA1-F, msTRIM21gRNA1-R, msTRIM21gRNA2-F, and msTRIM21gRNA2-R primers.

### Gene expression and silencing

*TRIM21* cDNA was amplified by PCR from a human cDNA library and subcloned into custom pcDNA3 or pLEX-MCS expression vectors with His-, Myc-, or EGFP- N-terminal tags. TRIM21^ΔRING^ (Δaa 1–66), TRIM21^ΔBBOX/CC^ (Δaa 69–261), and TRIM21^ΔPRY/SPRY^ (Δaa 262–475) were generated by PCR and cloned into pCDNA3 and pLEX-MCS expression vectors. Mouse *Trim21* full-length (aa 1–462), ΔRING (aa 89–462), ΔbBOX/CC (aa 1–88_250–462), and ΔPRY/SPRY (aa 1–277) were synthesized with a His tag. and cloned into pHIV-IRES-dTomato-based expression vectors by Azenta Life Sciences. ASC, ASC^PYD^, or ASC^CARD^ expression constructs have been described previously^80^. ASC was amplified by PCR and subcloned into pLEX-MCS expression vector with a mScarlet N-terminal tag. pcDNA3 expression plasmids encoding pro-caspase-1 and NLRP3 have been described previously^80,81^. *Ubiquitin C* cDNA was amplified by PCR from a human cDNA library and subcloned into pcDNA3 expression plasmid with a N-terminal His-tag. Ubiquitin (Addgene, plasmid 17608), ubiquitin-KO (Addgene, plasmid 17603), ubiquitin-K33 (Addgene, plasmid 17607), ubiquitin-K48 (Addgene, plasmid 17605), and ubiquitin-K63 (Addgene, plasmid 17606) tagged with N-terminal HA epitope tag in pRK5^82^, and pRK5 expressing N-terminal HA epitope-tagged ubiquitin-K6 (Addgene, plasmid 22900), ubiquitin-K11 (Addgene, plasmid 22901), ubiquitin-K27 (Addgene, plasmid 22902), ubiquitin-K29 (Addgene, plasmid 22903)^83^. All expression constructs were sequence verified.

### Lentivirus production and cell transduction

Lentiviral virions were produced by transfecting lentiviral expression constructs into HEK293T-Lenti cells (Takara Bio, 632180) along with pMD.2G and psPAX2 (Addgene, plasmids 12259 and 12260). 48 h post-transfection, lentiviral suspensions were harvested, passed through 0.45 μm filters and mixed with THP-1 cells in the presence of 0.5 μg mL^−1^ polybrene. Cells were centrifuged at 700 × *g* for 50 min at 32 °C. After overnight incubation at 37 °C, the medium was changed, and cells were sorted 72 h post-infection.

### RNAi-mediated silencing of *TRIM21*

Primary human macrophages in 24-well dishes (2.5 × 10^5^ cells per well) were transfected with 120 nM siRNA duplexes (hTRIM21 stealth siRNA, Invitrogen, 1299001) and non-targeting stealth control siRNAs (Invitrogen, 12935300) using F2 reagent (1.5 μL) plus Virofect (3.125 μL) (Targeting Systems, 007). THP-1 cells (3.3 × 10^5^ cells per well) were electroporated (Neon, Invitrogen, MPK10096) with 60 nM siRNA duplexes. Cells were analyzed 72 h post-transfection.

### Cell stimulation for cytokine release

THP-1 cells were seeded at a density of 3 × 10^5^ cells in 48-well plates. Cells were primed with ultrapure LPS from *E. coli* Serotype O111:B4 (1 μg mL^−1^, Invivogen, tlrl-3pelps) for 3 h followed by activation with nigericin (10 μM, Invivogen, tlrl-nig) for 30 min, ATP (5 mM, Sigma-Aldrich, A6419) for 20 min, CPPD crystals (125 μg mL^−1^, Invivogen, tlrl-cppd) for 6 h, silica crystals (400 μg mL^−1^, Invivogen, tlrl-sio-2) for 6 h, Rho inhibitor TcdB (2 μg mL^−1^, Cytoskeleton, CT04) for 4 h. Poly(dA:dT) (2 μg mL^−1^, Invivogen, tlrl-patn-1), *Bacillus anthracis* Lethal Factor (1 μg mL^−1^, List Biological Laboratories, 172 L), Protective Antigen (1 μg mL^−1^, List Biological Laboratories, 171E), and ultrapure flagellin from *Salmonella* enterica subsp. enterica serovar Typhimurium (100 ng mL^−1^, Invivogen, tlrl-epstfla) were transfected into THP-1 cells using Lipofectamine 2000 (0.8 μL/well, Invitrogen, 11668019) for 6 h or 16 h as indicated. After stimulation, cell-free supernatants were harvested for ELISA. BMDM were seeded at a density of 3 × 10^5^ cells in 48-well plates. Cells were primed with Pam3CSK4 (1 μg mL^−1^, 3 h, Invivogen, tlrl-pms) and activated with nigericin (3 μM, 30–60 min). iBMDM were seeded at a density of 3 × 10^5^ cells in 48-well plates. Cells were primed with ultrapure LPS from *E. coli* Serotype O111:B4 (0.5 μg mL^−1^, 4 h) and activated with nigericin (2.5 μM, 30 min).

### Immunostaining and flow cytometric detection of polymerized ASC and TRIM21

ASC polymerization was analyzed as previously described^29^. THP-1 cells (2 × 10^6^ cells) were seeded in 6-well plates, primed with Pam3CSK4 (1 μg mL^−1^, 4 h) and activated with nigericin (10 μM, 30 min). Cells were washed with 1× PBS and fixed and permeabilized using Cytofix/Cytoperm reagent (BD Biosciences, 554714) for 15 min at room temperature in the dark. After washing the cells once with Perm/Wash buffer (BD Biosciences, 554723), non-specific Fc receptor binding was blocked by adding Human TruStain FcX 1:50 v/v (BioLegend, 422302) and incubation on ice for 10 min. Cells were then stained with mouse monoclonal PE-conjugated anti-ASC antibody (50 ng mL^−1^, BioLegend, 653903) and goat polyclonal anti-TRIM21 antibody (1 μg mL^−1^, Invitrogen, PA5-18147) for 20 min on ice in the dark. After washing the cells twice with cold autoMACS Running Buffer (MACS, Miltenyi Biotec, 130-091-221), cells were stained with AlexaFluor (AF) 647-conjugated donkey anti-goat antibody (2 μg mL^−1^, Invitrogen, A-21447) for 20 min on ice in the dark. After washing twice with cold MACS buffer, cells were resuspended in cold MACS buffer, passed through 40 μm cell strainers, and analyzed using an Aurora Northern Lights spectral cytometer (Cytek), with capture of height and width signals for PE and AF647 emission peak detectors, Blue4 and Red2, respectively.

### Flow cytometric detection of polymerized ASC and TRIM21 particles

GFP-TRIM21 and mScarlet-ASC stably expressing THP-1 cells (2 × 10^6^ cells) were seeded in 6-well plates, primed with ultrapure LPS (0.5 μg mL^−1^, 2 h) and activated with nigericin (10 μM, 30 min). Cells were washed with 1× PBS, fixed, and resuspended in MACS buffer as described above. Cells were passed through 40 μm cell strainers and analyzed using an Aurora Northern Lights spectral cytometer (Cytek). Male and female mice were euthanized at 17 days old (*Nlrp3*^A350V^) and at 30–90 days old (*Mefv*^V726A^) and blood collected by intracardiac puncture. After incubating the samples at room temperature for 15–30 min to allow coagulation, blood samples were centrifuged at 4 °C at 1200 × *g* for 10 min and serum collected. 10 μL of serum was mixed with 500 μL of cold 1× PBS, centrifuged at 4 °C at 19,800 × *g* for 10 min to pellet particles. Pellets were resuspended in 89 μL cold MACS buffer and immunostained with mouse monoclonal anti-ASC antibody (400 ng mL^−1^, Santa Cruz Biotechnology, sc-514414) and rabbit polyclonal anti-TRIM21 antibody (1 μg mL^−1^, Novus Biologicals, NBP3-03809) for 20 min on ice in the dark. After washing the samples twice with cold MACS buffer, cells were stained with AF647-conjugated donkey anti-mouse antibody (2 μg mL^−1^, Invitrogen, A-31571) and AF488-conjugated donkey anti-rabbit antibody (2 μg mL^−1^, Invitrogen, A-21206) for 20 min on ice in the dark. Samples were washed and analyzed as above. WT THP-1 cells (5 × 10^6^ cells) were seeded in T25 flasks, primed with ultrapure LPS (0.5 μg mL^−1^, 2 h) and activated with nigericin (10 μM, 30 min). After stimulation, cells and culture supernatant were harvested and centrifuged at 200 × *g* for 5 min at room temperature. Cell-free supernatants were centrifuged at 375 × *g* for 5 min at room temperature to pellet large debris and the supernatants transferred to new microcentrifuge tubes. Particles were then pelleted by centrifugation at 19,800 × *g* for 10 min at 4 °C, washed once with 1× PBS and resuspended in MACS buffer and immunostained with mouse monoclonal PE-conjugated anti-ASC antibody (50 ng mL^−1^, BioLegend, 653903) for 20 min on ice in the dark. After washing the particles twice with cold MACS buffer, particles were resuspended in cold MACS buffer, passed through 40 μm cell strainers and analyzed using an Aurora Northern Lights spectral cytometer (Cytek).

### Release of fluorescent polymerized ASC and TRIM21 particles

GFP-TRIM21 and mScarlet-ASC stably expressing THP-1 cells (5 × 10^6^ cells) were seeded in 24-well plates, pre-treated with PYR-41 (25 μM, Millipore-Sigma, 662105) or DSF (40 μM, Millipore-Sigma, 86720) for 4 h, primed with ultrapure LPS (0.5 μg mL^−1^, 2.5 h), and activated with nigericin (10 μM, 20–30 min). After cell stimulation, fluorescent signals for eGFP (excitation: 489 nm; emission: 511 nm) and mScarlet (excitation: 569 nm; emission: 594 nm) were measured in cell-free supernatants with a Varioskan Lux plate reader (Thermo Scientific).

### Polymerized ASC particle purification and stimulation of recipient THP-1 cells

THP-1 cells were seeded (5 × 10^6^ cells) in T25 flasks, primed with ultrapure LPS (0.5 μg mL^−1^, 2 h) and activated with nigericin (10 μM, 30 min) and cells and culture supernatant were harvested and centrifuged at 200 × *g* for 5 min at room temperature. Cell pellets were washed once with 1× PBS and then resuspended in Laemmli buffer. Supernatants were centrifuged at 375 × *g* for 5 min at room temperature to pellet large debris and the supernatants transferred to new microcentrifuge tubes. Particles were then pelleted by centrifugation at 19,800 × *g* for 10 min at 4 °C, washed once with 1× PBS and resuspended in Laemmli buffer. Total cell lysates and particles were analyzed by immunoblot. For activation of recipient cells, GFP-TRIM21 and mScarlet-ASC stably expressing THP-1 cells (20 × 10^6^ cells) were seeded in T25 flasks, primed with Pam3CSK4 (1 μg mL^−1^, 2.5 h) and activated with nigericin (10 μM, 45 min). After stimulation, cells and culture supernatant were harvested and centrifuged at 200 × *g* for 5 min at room temperature. Cell-free supernatants were centrifuged at 375 × *g* for 5 min at room temperature to pellet large debris and the supernatants transferred to new microcentrifuge tubes. Particles were then pelleted by centrifugation at 19,800 × *g* for 10 min at 4 °C, washed once with 1× PBS, and resuspended in sorting buffer (2% BSA, 0.5 mM EDTA, 25 mM HEPES in PBS). mScarlet-ASC^+^GFP-TRIM21^+^ particles were sorted into collection buffer (25 mM HEPES in PBS) and used for stimulation of recipient THP-1 cells primed with ultrapure LPS (0.5 μg mL^−1^, 3 h). Twelve hours post-stimulation with particles, cells, and culture supernatant were harvested and centrifuged at 200 × *g* for 5 min at 4 °C. Supernatants were transferred to new microcentrifuge tubes for analysis by ELISA.

### ASC polymerization assay by crosslinking

5 × 10^6^ THP-1 cells were primed with ultrapure LPS (1 μg mL^−1^, 2.5 h) and activated with nigericin (10 μM, 15 min). Cells were washed with ice-cold 1× PBS and lysed in conjugation buffer (20 mM HEPES, pH 7.4) by sheering through a 23-gauge needle. Total cell lysates were crosslinked by adding 2 mM disuccinimidyl suberate (DSS, Thermo Scientific, A39267) and incubated for 30 min at room temperature. The reaction was quenched in 50 mM Tris-HCl pH 7.5 for 15 min at room temperature. After centrifugation at 5000 × *g* for 8 min at 4 °C, crosslinked pellets were resuspended in non-reducing sample buffer (125 mM Tris-HCl pH 6.8, 20% glycerol, 4% SDS, 0.1% bromophenol blue) and analyzed by immunoblot for ASC.

### Inflammasome reconstitution assay

HEK293 cells were transfected with Lipofectamine 2000 and pcDNA3-based expression constructs for pro-IL-1β, pro-caspase-1, ASC, NLRP3, and TRIM21 and IL-1β in the culture supernatant was quantified by ELISA, as described earlier^74^.

### ELISA

Cell-free supernatants from primed and activated THP-1 cells, BMDM, or iBMDM were analyzed for IL-1β (Invitrogen, 88-7261-22 (hIL-1β), 88-7013-22 (mIL-1β), IL-18 (R&D systems, DY318-05 (hIL-18), TNF (Invitrogen, 887346-76 (hTNF), 88-7324-88 (mTNF)), or IL-6 (BD Biosciences, 555220 (hIL-6)). To measure levels of mIL-18, high-binding plates were coated with rat monoclonal anti-IL-18 antibody (1 μg mL^−1^, MBL, MBL-D047-3) and incubated overnight at 4 °C. All further incubations were performed at room temperature. After 3 washes in 0.05% Tween-20 in 1× PBS, plates were blocked using 1× ELISA/ELISPOT diluent (Invitrogen, 00-4202-56) for 1 h. Samples, diluted if needed in 1× ELISA/ELISPOT diluent were then added onto the plate and incubated for 2 h. After 3 washes, detection antibodies were added, i.e., biotinylated rat monoclonal anti-IL-18 antibody (1:2000, MBL, MBL-D048-6), and the plates sealed and incubated for 2 h. Plates were washed 3 times and Avidin-HRP (Invitrogen, 18-4200-89) was added and the plates incubated for 30 min. After 6 washes, 1× tetramethylbenzidine (TMB) substrate (Invitrogen, 00-4201-56) was added and the reaction stopped with 2 N sulfuric acid solution. Optical densities were measured at 490 nm as well as 570 nm for background subtraction with a Varioskan Lux plate reader (Thermo Scientific). Human plasma samples from healthy donors and CAPS patients were analyzed for ASC and TRIM21. High binding plates were coated with mouse monoclonal anti-ASC antibody (200 ng mL^−1^, Santa Cruz Biotechnology, sc-514414) or goat polyclonal anti-TRIM21 antibody (500 ng mL^−1^, Invitrogen, PA5-18147) and incubated overnight at room temperature. All incubations were performed at room temperature. After 3 washes in 0.05% Tween-20 in 1× PBS, plates were blocked using 1× ELISA/ELISPOT diluent for 1 h. Samples, diluted if needed in 1× ELISA/ELISPOT diluent were then added onto the plate and incubated for 2 h. After 3 washes, detection antibodies were added, i.e., rabbit polyclonal anti-ASC (1 μg mL^−1^, Adipogen, AG-25B-0006-C100) or rabbit monoclonal anti-TRIM21 antibody (606 ng mL^−1^, Abcam, ab207728), and the plates sealed and incubated for 2 h. After 3 washes, biotin-conjugated donkey anti-rabbit antibody (750 ng mL^−1^, Invitrogen, A-16039) was added, and the plates incubated for an additional 30 min. Plates were washed 3 times and Avidin-HRP was added and the plates incubated for 30 min. After 6 washes, 1× TMB substrate was added and the reaction stopped with 2 N sulfuric acid solution. Optical densities were measured at 490 nm as well as 570 nm for background subtraction with a Varioskan Lux plate reader (Thermo Scientific). Human anti-TRIM21 IgG was quantified using the Human Ro52 Autoantibody AssayMax ELISA Kit (AssayPro, ER7501-1) and mouse anti-TRIM21 IgG was quantified using the Mouse Anti-SSA/Ro52 (TRIM21/RNF81) IgG ELISA Kit (Alpha Diagnostic, 5730).

### Human autoantibody epitope mapping

*TRIM21*^KO^ THP-1 cells (40 × 10^6^ cells mL^−1^) restored with GFP-TRIM21, GFP-TRIM21^ΔRING^, GFP-TRIM21^ΔbBox/CC^ or GFP-TRIM21^ΔPRY/SPRY^ were lysed by 40× passages through 1 mL pipette tips in hypotonic lysis buffer (10 mM HEPES, pH 7.5, 10 mM KCl, 1.5 mM MgCl2, 0.5 mM DTT, 7% glycerol, 1 mM sodium orthovanadate, 1 mM phenylmethylsulfonyl fluoride, protease inhibitor cocktail (Thermo Scientific, A32963)). Cell lysates were precleared by centrifugation at 19,800 × *g* for 10 min at 4 °C. High binding plates were coated with mouse monoclonal anti-GFP antibody (200 ng mL^−1^, Santa Cruz Biotechnology, sc-9996) and incubated overnight at 4 °C. After 3 washes in 0.05% Tween-20 in 1× PBS, plates were blocked using 1× ELISA/ELISPOT diluent for 1 h at room temperature. Cell lysates were loaded onto the plate and incubated overnight at 4 °C. After 3 washes, plasma samples from healthy donors or CAPS patients were diluted (1:10) in 1× ELISA/ELISPOT diluent, added onto the plate, and incubated for 2 h at room temperature. After 3 washes, biotin-conjugated anti-Human IgG antibody (AssayPro, ER7501-1) was added onto the plate and incubated for 30 min at room temperature. Plates were washed 3 times and Avidin-HRP was added and the plates incubated for 30 min. After 6 washes, 1× TMB substrate was added, and the reaction stopped with 2 N sulfuric acid solution. Optical densities were measured at 450 nm as well as 570 nm for background subtraction with a Varioskan Lux plate reader (Thermo Scientific).

### Mouse autoantibody epitope mapping

HEK293 cells (2 × 10^6^ cells) in 6-well plates were transiently transfected with Lipofectamine 2000 and His-m*Trim21* full-length, His-m*Trim21*^ΔRING^, His-m*Trim21*^ΔbBOX/CC^, or His-m*Trim21*Δ^PRY/SPRY^. Forty-eight hours post-transfection, mTRIM21 proteins were purified by Ni^2+^ affinity purification with magnetic beads (Thermo Scientific, 88832) as described above. Immunoglobulins (Ig) were affinity-purified from mouse serum with Protein A/G agarose beads (Santa Cruz Biotechnology, sc-2003) overnight on a rocking platform at 4 °C and washed once in 1× ice-cold PBS. mTRIM21-bound magnetic beads were mixed (1:1 volume) with Ig-bound agarose beads overnight on a rocking platform at 4 °C. mTRIM21/Ig complexes were captured on a magnetic rack, washed once in 1× ice-cold PBS, and resuspended in 1× ice-cold PBS. mTRIM21/Ig complexes were stained using AF488-conjugated donkey anti-mouse IgG antibody (2 μg mL^−1^, Invitrogen, A-21202) for 20 min on ice in the dark. After one wash in 1× ice-cold PBS, mTRIM21/Ig complexes were resuspended in 1× ice-cold PBS for fluorescence reading (OD_exc_ 490 nm, OD_em_ 520 nm).

### Quantitative real-time PCR

THP-1 cells or BMDM (8 × 10^5^ cells/well were seeded in 12-well plates and primed with ultrapure LPS (0.1–1 μg mL^−1^, 4 h). Cells were washed with ice-cold 1× PBS. Total RNA was isolated using Phenol/Chloroform extraction. Purified RNA samples (1 μg) were then subjected to reverse transcription using Verso cDNA synthesis kit (Thermo Scientific, AB1453B). Taqman-based multiplexed gene expression analysis was performed on a QuantStudio 3 (ThermoFisher Scientific) using FAM-labelled exon-spanning assays, normalized to VIC-labelled *ACTB* (Invitrogen, Hs99999903, Mm04394036).

### Fluorescence analysis of ASC and TRIM21 subcellular localization

HEK293 cells were transiently transfected with epitope-tagged cDNAs and then seeded (2 × 10^5^ cells) onto coverslips in 12-well plates. Cells were fixed (3.7% paraformaldehyde in PBS, 15 min, room temperature), permeabilized (ice-cold 0.5%Triton ×-100 in PBS, 10 min, room temperature) and blocked (2% BSA, 2% (v/v) goat sera (Fisher, NC9660079), 5% (v/v) donkey sera (Fisher, NC9719162), 0.5% Triton ×-100 in PBS, 1 h, room temperature). Coverslips were then immunostained overnight with rabbit monoclonal anti-Myc (400 ng mL^−1^, Cell Signaling Technology, 2278), rat monoclonal anti-HA (1 μg mL^−1^, Roche, 11867423001) and mouse monoclonal anti-Flag M2 (1 μg mL^−1^, Sigma-Aldrich, F1804) in a humidified chamber, washed and subsequently incubated with AF-conjugated secondary antibodies (1–2 μg mL^−1^, Invitrogen) for 1 h, washed and mounted onto slides with Prolong glass antifade with Nucblue stain (Invitrogen, P36981). Images were captured on a Nikon TE2000E2-PFS with Å~60 and Å~100 oil objectives) with image deconvolution (Nikon, NIS Elements). GFP-TRIM21 and mScarlet-ASC stably expressing THP-1 cells were seeded (3 × 10^5^) on 18-mm diameter glass coverslips in 12-well plates, differentiated with phorbol 12-myristate-13-acetate (PMA, 200 ng/mL o/n, Invitrogen, J63916.MCR) and rested for 48 h. Cells were primed with ultrapure LPS (0.5 μg mL^−1^, 2 h) and activated with nigericin (5 μM, 5–45 min). Cells were washed with 1× PBS and fixed with 4% paraformaldehyde (Electron Microscopy Sciences, 15713S) for 15 min at room temperature in the dark. Cells were washed 3 times in 1× PBS for 5 min and mounted using ProLong Glass Antifade with NucBlue stain. Images were captured using a Nikon Eclipse TE2000-E microscope with 40, 60, or 100× oil immersion objectives (Nikon, NIS Elements). Z-stack images were acquired with a step size of 0.3 μm across the entire cell volume (40 optical sections). Image stacks were subjected to deconvolution with the Richardson-Lucy method and 3D reconstruction (wide-field modality, iterations 90, calibration 0.065 μm) using NIS Elements. Analyses and quantifications were performed using NIS Elements and ImageJ (Fiji).

### Caspase-1 activity assay by FLICA

THP-1 cells were seeded (2 × 10^6^ cells) in 6-well plates and primed with 1 μg mL^−1^ ultrapure LPS for 40 min. Priming was continued for another 20 min in the presence of biotinylated pan-caspase inhibitor (10 μM, AnaSpec, AS-60841). Cells were then activated with nigericin (10 μM) for 20 min. Cells were washed once in 1× PBS and twice with cold MACS buffer. Cells were fixed and permeabilized using Cytofix/Cytoperm buffer for 15 min at room temperature in the dark. After washing the cells once with Perm/Wash buffer, cells were resuspended in Perm/Wash buffer and stained with AF647-conjugated streptavidin (400 ng mL^−1^, Invitrogen, S21374) on ice for 20 min in the dark. Cells were washed twice and resuspended in MACS buffer and analyzed using an Aurora Northern Lights spectral cytometer (Cytek).

### Lactate dehydrogenase cytotoxicity assay

Lactate dehydrogenase (LDH) release was assessed in the culture supernatant using CyQUANT LDH Cytotoxicity Assay kit (Invitrogen, C203021). Cytotoxicity is calculated and displayed as the percentage of LDH release compared to the total amount of LDH released upon cell lysis.

### Ni^2+^ affinity purification

HEK293 cells (2 × 10^6^ cells) were seeded in 6-well plates and transiently transfected. 48 h post-transfection, cells were collected, washed once in 1× ice-cold PBS and lysed by sonication in lysis buffer (50 mM Tris-HCl, pH 8.0, 200 mM NaCl, 1% NP-40, 1% sodium deoxycholate, 100 mM imidazole, 0.1% SDS, 0.05% Tween-20, 30 mM sodium pyrophosphate, 50 mM sodium fluoride, 1 mM sodium orthovanadate, 1 mM phenylmethylsulfonyl fluoride, protease inhibitor cocktail, pH 8.0). Cell lysates were precleared by centrifugation at 19,800 × *g* for 10 min at 4 °C. Magnetic Ni-NTA agarose beads were added to each sample and incubated for 2 h on a rocking platform at 4 °C. Beads were magnetically collected, washed 4 times with wash buffer (50 mM Tris-HCl, pH 8.0, 200 mM NaCl, 1% NP-40, 1% sodium deoxycholate, 200 mM imidazole, 0.1% SDS, 0.05% Tween-20, 30 mM sodium pyrophosphate, 50 mM sodium fluoride, 1 mM sodium orthovanadate, 1 mM phenylmethylsulfonyl fluoride, protease inhibitor cocktail, pH 8.0). Bound proteins were eluted in elution buffer (50 mM Tris-HCl, pH 8.0, 200 mM NaCl, 1% NP-40, 1% sodium deoxycholate, 400 mM imidazole, 0.1% SDS, 0.05% Tween-20, 30 mM sodium pyrophosphate, 50 mM sodium fluoride, 1 mM sodium orthovanadate, 1 mM phenylmethylsulfonyl fluoride, protease inhibitor cocktail, pH 8.0) and Laemmli buffer and analyzed by immunoblotting.

### Immunoblotting

Cells were collected and washed once with ice-cold 1× PBS and lysed in lysis buffer (50 mM Tris-HCl, pH 8.0, 150 mM NaCl, 1% NP-40, 30 mM sodium pyrophosphate, 50 mM sodium fluoride, 1 mM sodium orthovanadate, 1% NP-40, 1 mM phenylmethylsulfonyl fluoride, protease inhibitor cocktail), and precleared by centrifugation at 19,800 × *g* for 10 min at 4 °C. Proteins were separated on polyacrylamide gels and transferred onto PVDF membranes (Millipore-Sigma, IPVH00010). Membranes were blocked (5% non-fat dry milk, 0.1 M Tris-buffered saline, pH 7.4, 0.1% Tween 20) for 45 min at room temperature followed by incubation with primary antibodies as indicated overnight at 4 °C: rabbit monoclonal antibody to TRIM21 (Abcam, ab207728, clone #EPR20290), rabbit polyclonal antibody to ASC (Adipogen, AG-25B-0006-C100, AL-177; Sigma-Aldrich AB3607), mouse monoclonal antibody to ASC (Santa-Cruz Biotechnology, sc-514414, clone #B-3), mouse monoclonal antibody to NLRP3 (Adipogen, AG-20B-0014-C100, clone #Cryo-2), mouse monoclonal antibody to cleaved caspase-1 (p20)/pro-caspase-1 (Adipogen, AG-20B-0048-C100, clone #Bally-1), mouse monoclonal antibody to cleaved caspase-1 (p20)/pro-caspase-1 (Adipogen, AG-20B-0042-C100, clone #Casper-1), rabbit antibody to β-tubulin (Cell Signaling Technology, 2146), rabbit monoclonal antibody to cleaved GSDMD (Cell Signaling Technology, 36425, clone E7H9G), rabbit monoclonal antibody to GSDMD (Cell Signaling Technology, 93709, clone L60), rabbit monoclonal antibody to cleaved/total GSDMD (Abcam, ab209845, clone #EPR19828), mouse monoclonal antibody to Myc (Cell Signaling Technology, 2276, clone 9B11), mouse monoclonal antibody to GFP (Santa-Cruz Biotechnology, sc-9996, clone B-2) and rabbit monoclonal antibody to GAPDH (Cell Signaling Technology, 2118, clone 14C10). Antibodies were diluted 1:1,000. Membranes were washed and incubated with HRP-conjugated secondary antibodies, including goat anti-rabbit IgG (H + L) HRP (Cell Signaling Technology, 7074) or horse anti-mouse IgG (H + L) HRP (Cell Signaling Technology, 7076) diluted 1:5000 when necessary for 1 h at room temperature and proteins were visualized by ECL detection (Super Signal West Femto, ThermoFisher Scientific, 34096) and digital image acquisition (Thermo iBright and Ultralum Omega 14vR).

### Protein concentration and precipitation

Serum-free culture supernatants were collected and adjusted to 5% (v/v) ice-cold trichloroacetic acid (VWR, BDH7372-2), incubated on ice for 10 min and centrifuged at 19,800 × *g* for 5 min at 4 °C. Pellets were washed twice with ice-cold acetone, briefly air-dried, resuspended in Laemmli buffer, sonicated, and analyzed by immunoblot.

### Co-Immunoprecipitation

THP-1 cells (10 × 10^6^ cells) were lysed (50 mM Tris-HCl, pH 8.0, 150 mM NaCl, 5 mM EDTA, 1% NP-40, 50 mM sodium fluoride, 1 mM sodium orthovanadate, 1 mM phenylmethylsulfonyl fluoride, protease inhibitor cocktail) and lysates were precleared by centrifugation at 19,800 × *g* for 10 min at 4 °C. Total cell lysates or cell-free supernatants were incubated with 1 μg of rabbit polyclonal antibody to ASC (Adipogen, AG-25B-0006-C100, AL-177) on a rocking platform for 2 h at 4 °C. Protein A/G magnetic beads were added to the samples and incubated for 12 h on a rocking platform at 4 °C. Beads were magnetically collected, washed 3 times with lysis buffer and bound proteins were eluted in Laemmli buffer and analyzed by immunoblotting. Human primary macrophages were transfected with Ctrl siRNA or *TRIM21* siRNA and left untreated or primed with LPS (100 ng mL^−1^, 90 min). After lysis (50 mM Tris-HCl, pH 8.0, 150 mM NaCl, 5 mM EDTA, 1% NP-40, 50 mM sodium fluoride, 1 mM sodium orthovanadate, 1 mM phenylmethylsulfonyl fluoride, protease inhibitor cocktail), lysates were precleared by centrifugation at 19,800 × *g* for 10 min at 4 °C. Total cell lysates or cell-free supernatants were incubated with 1 μg of rabbit polyclonal antibody to ASC (Adipogen, AG-25B-0006-C100, AL-177) or rabbit IgG isotype control (Invitrogen, 02-6102) on a rocking platform for 2 h at 4 °C. Protein A/G magnetic beads were added to the samples and incubated for 12 h on a rocking platform at 4 °C. Beads were magnetically collected, washed 3 times with lysis buffer, and bound proteins were eluted in Laemmli buffer and analyzed by immunoblotting.

### Human subjects

RNAseq data were analyzed from a previously published data set (GEO accession GSE57253)^36^. GSE57253 is comprised of RNAseq results of mRNA isolated from whole blood cells obtained from five healthy pediatric controls and seven patients with NOMID with active disease before anakinra treatment and the same seven patients with NOMID with inactive disease after anakinra treatment. De-identified plasma samples from healthy donors (HD) and patients with active flares of Familial Cold Autoinflammatory Syndrome (FCAS), and/or Muckle–Wells Syndrome (MWS), or NOMID were obtained under a protocol approved by the University of California San Diego, and the Rady Children’s Hospital-San Diego, and approved by the Institutional Review Board at Cedars-Sinai Medical Center and stored at −80 °C (Supplementary Table 2). Informed consent was obtained from participants, who were selected based on their genetics and disease status without any bias.

### Proximity ligation assay

Proximity ligation assay (PLA, Duolink In Situ PLA, Sigma-Aldrich, DUO92101-1KT) was performed as per the manufacturer’s instructions. THP-1 cells (3.3 × 10^5^) were seeded in 12-well plates on glass coverslips and differentiated with PMA (200 ng/mL, o/n), washed in PBS and rested for 48 h. After priming with ultrapure LPS (0.5 μg mL^−1^) for 2 h, cells were activated with nigericin (10 μM, 20 min). Cells were washed with PBS, fixed with 4% paraformaldehyde (10 min, room temperature), permeabilized with ice-cold 0.1% Triton ×-100 (10 min, room temperature) and washed with PBS. Unless specified, all incubations were performed at 37 °C in a humidified chamber. Cells were blocked using the Duolink Blocking Solution for 1 h and incubated with mouse/rabbit combinations of primary antibodies, rabbit monoclonal anti-TRIM21 (2.96 μg mL^−1^, Invitrogen, PA5-120224), and mouse monoclonal anti-ASC (2.96 μg mL^−1^, Santa Cruz Biotechnology, sc-514414) for 12 h at 4 °C in a humidified chamber. After washing with PBS, coverslips were incubated with PLUS and MINUS PLA probes, washed, incubated with the ligase for 30 min, washed and incubated with the polymerase for 100 min. After washing, samples were mounted on slides using the Duolink In Situ Mounting Medium with DAPI. Coverslips were imaged on a Nikon Eclipse TE2000-E2 and the data processed and analyzed using the NIS-Elements AR (Nikon) and Fiji (ImageJ) software.

### Statistics

All in vitro experiments have been repeated at least 3 times, and the in vivo experiments 2 times and graphs were prepared in Prism 10 (GraphPad) and represent the mean ± s.d. A standard parametric two-tailed unpaired *t*-test was used for statistical analysis of two groups and one-way ANOVA with Dunnett’s post-test for comparison of 3 or more groups with all data points showing a normal distribution. Data were also analyzed by linear regression and presented as R squared. Survival was determined by Log-rank Mantel–Cox test. Values of *P* ≤ 0.05 were considered significant and marked as *P ≤ 0.05, **P ≤ 0.01, ***P ≤ 0.001, and ****P ≤ 0.0001. All exact *P* values are available as statistical source data.

### Reporting summary

Further information on research design is available in the Nature Portfolio Reporting Summary linked to this article.

## Supplementary information

Supplementary Information

Transparent Peer Review file

Reporting Summary

## Source data

Source Data

## Data Availability

All data supporting the findings of this study are available within the paper and its Supplementary Information and source data are provided with this paper for all data. Primer sequences are provided in Supplementary Table 1. Human subject information is provided in Supplementary Table 2. The reagents and key resources used are detailed in Supplementary Table 3. Public RNAseq data were analyzed from a previously published data set (GEO accession GSE57253,https://www.ncbi.nlm.nih.gov/geo/query/acc.cgi?acc=GSE57253)^36^. Source data are provided with this paper.
