# Supplementary material for: TRIM21 facilitates inflammasome assembly and contributes to autoinflammatory disease
Source: Nat Commun. 2026 May 22;17:6726. doi: 10.1038/s41467-026-73350-3 (PMC13385917; doi:10.1038/s41467-026-73350-3)
Supplement: Supplementary file 1 — Supplementary Information [file 41467_2026_73350_MOESM1_ESM.pdf]

**TRIM21 facilitates inflammasome assembly and contributes to autoinflammatory disease.**

SUPPLEMENTARY INFORMATION

## SUPPLEMENTARY FIGURES

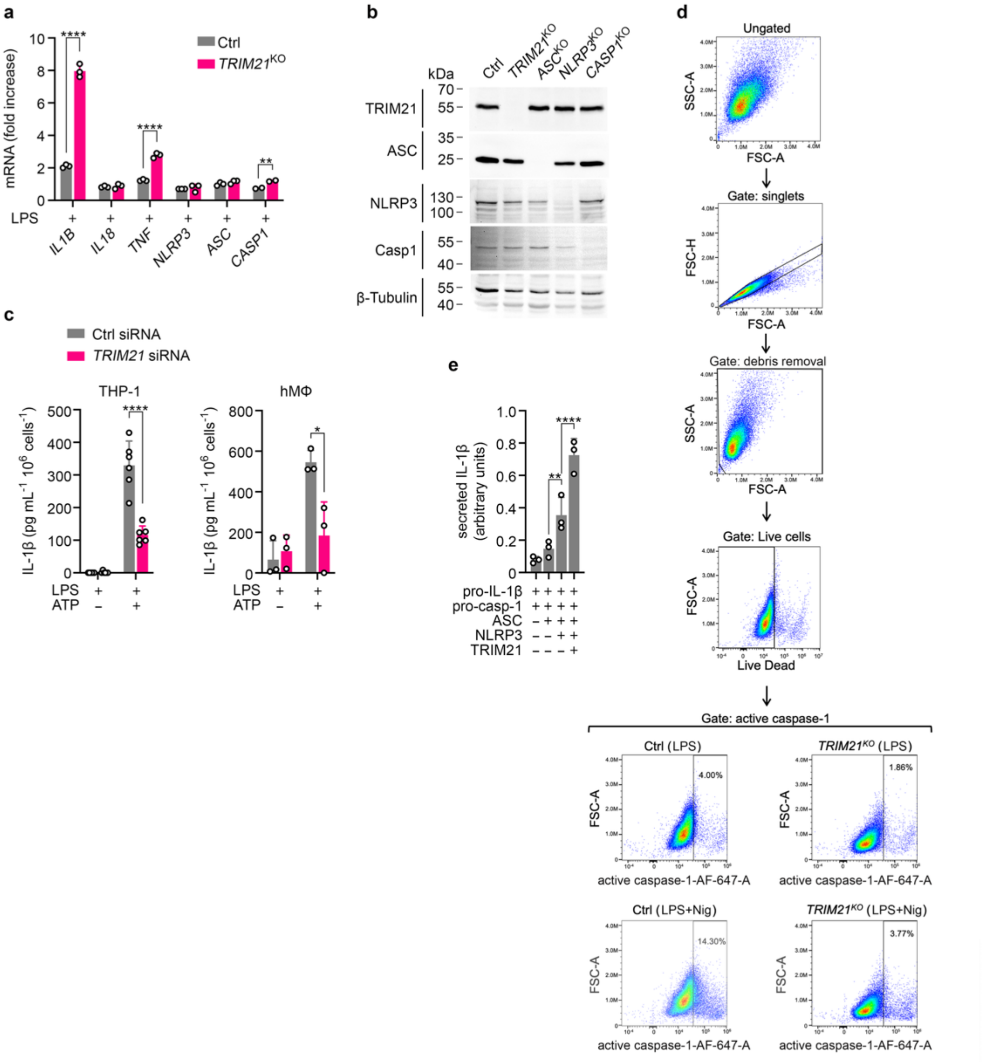


**Supplementary Fig. 1 | TRIM21 is required for inflammasome activation.**
**a** Quantitative RT-PCR for *IL1B*, *IL18*, *TNF*, *NLRP3*, *ASC* and *CASP1* of mRNA isolated from Ctrl and *TRIM21*$^{KO}$ THP-1 cells primed with LPS (1mg mL$^{-1}$, 4hr). (n=3 biological replicates, mean±s.d., parametric two-tailed unpaired t-test). **b** Immunoblot for TRIM21, ASC, NLRP3, Casp1 and β-tubulin using TCL from Ctrl, *TRIM21*$^{KO}$, *ASC*$^{KO}$, *NLRP3*$^{KO}$ and *CASP1*$^{KO}$ THP-1 cells. **c** IL-1β ELISA of cleared culture supernatants (SN) of THP-1 cells and primary human macrophages (hMΦ) transfected with control (Ctrl) or *TRIM21* siRNA and primed with LPS (1μg mL$^{-1}$, 4hr) or primed and then activated with ATP (5mM, 30min). (n=3-6 biological replicates, mean±s.d., parametric two-tailed unpaired t-test). **d** Gating strategy used for the FLICA in Fig. 2d of Ctrl and *TRIM21*$^{KO}$ THP-1 cells primed with LPS (1μg mL$^{-1}$, 4hr) or primed and activated with nigericin (Nig, 10μM, 20min). Total cells were gated for singlets, intact cells, live cells and active caspase-1. This gating strategy was used for all samples. **e** IL-1β ELISA of cleared SN from HEK293 cells with reconstituted NLRP3 inflammasomes by transient transfection of pro-IL-1β, pro-caspase-1, ASC, NLRP3 and TRIM21 as indicated (n=3 biological replicates, mean±s.d., parametric two-tailed unpaired t-test). Results **(a-c, e)** representative of n=3 experiments.

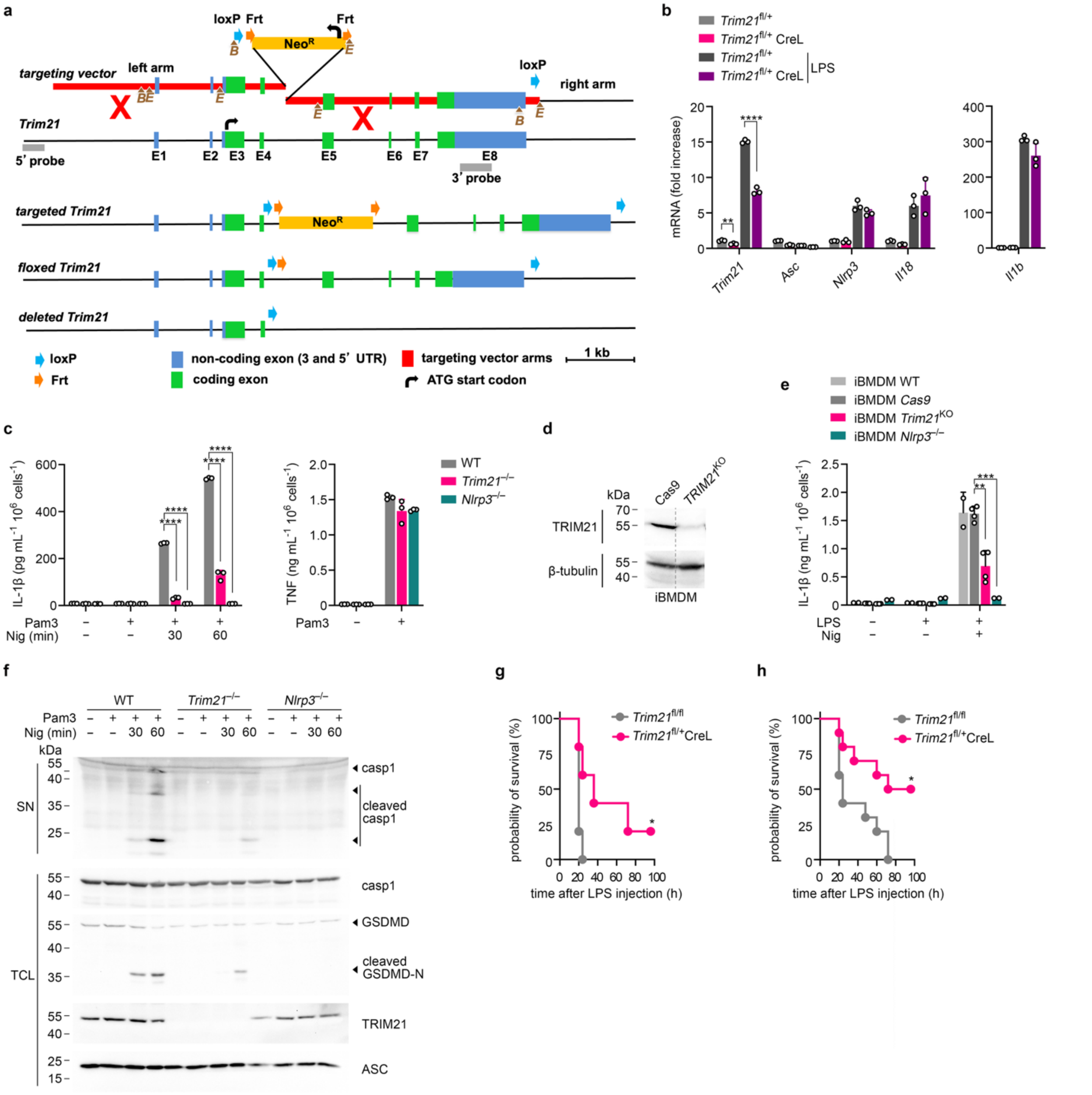


**Supplementary Fig. 2 | TRIM21 is required for inflammasome activation *in vivo*.**
**a** Schematic scheme of the strategy used to delete *Trim21* in mice. E, exon, Neo$^R$, neomycin resistance cassette. The red cross indicates where genomic recombination occurs. **b** Quantitative RT-PCR for *Trim21*, *Asc*, *Nlrp3*, *Il1b* and *Il18* mRNA isolated from LPS- (100ng mL$^{-1}$, 4hr) primed *Trim21*$^{fl/+}$ and *Trim21*$^{fl/+}$ CreL BMDM. (n=3 biological replicates, mean±s.d., parametric two-tailed unpaired t-test). **c** IL-1$\beta$ and TNF ELISA of cleared culture SN from WT, *Trim21*$^{–/–}$ and *Nlrp3*$^{–/–}$ BMDM left untreated, primed with Pam3CSK4 (Pam3, 1$\mu$g mL$^{-1}$, 3hr) or primed and activated with nigericin (Nig, 3$\mu$M, 30 or 60min). (n=3 biological replicates, mean±s.d., one-way ANOVA with Dunnett's post-test). **d** Immunoblot of TRIM21 and $\beta$-tubulin loading control from total cell lysates from Cas9 control and *Trim21*$^{KO}$ immortalized iBMDM. Both samples were separated on the same gel with additional samples in between. **e** IL-1$\beta$ ELISA of cleared culture SN from WT, Cas9 control, *Trim21*$^{KO}$ and *Nlrp3*$^{–/–}$ iBMDM left untreated, primed with LPS (0.5$\mu$g mL$^{-1}$, 4hr) or primed and

activated with Nig (2.5μM, 30min). (n=3 biological replicates, mean±s.d., one-way ANOVA with Dunnett's post-test). **f** Immunoblot of cleaved and total caspase-1 (casp1), cleaved and total gasdermin D (GSDMD), TRIM21 and ASC from SN or TCL from WT, *Trim21*$^{-/-}$ and *Nlrp3*$^{-/-}$ BMDM left untreated, primed with Pam3 (1μg mL$^{-1}$, 3hr) or primed and activated with Nig (3μM, 30 or 60min). **g-h** Survival of female (**g**) and male and female (**h**) *Trim21*$^{fl/+}$ and *Trim21*$^{fl/+}$CreL mice following i.p. injection of LPS (20mg kg$^{-1}$) presented as Kaplan-Meier estimate and Log-rank (Mantel-Cox) test (g: n=5 mice, h: n=10 mice). Results representative of n=2 **(g,h)** or n=3 **(b-f)** experiments.

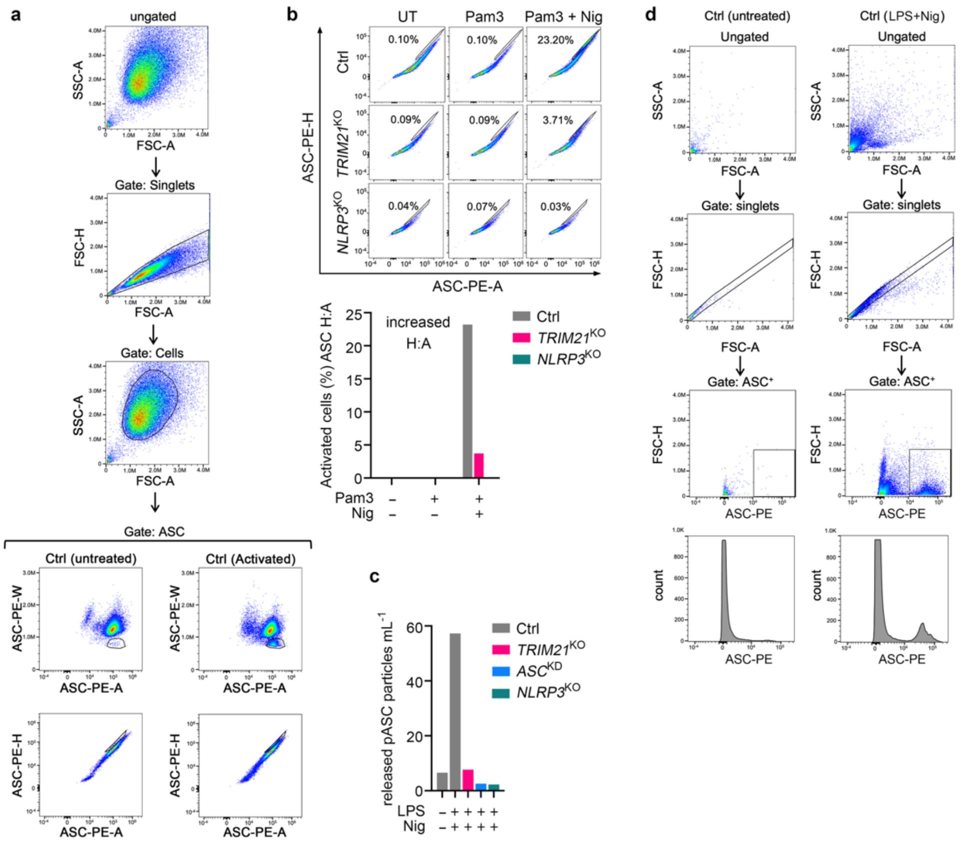


**Supplementary Fig. 3 | TRIM21 promotes ASC polymerization and release.**
**a** Gating strategy used for the flow cytometric detection of polymerized ASC (Fig. 4a, Supplementary Fig. 3b) in control (Ctrl), *TRIM21*$^{KO}$, and *NLRP3*$^{KO}$ THP-1 cells primed with Pam3CSK4 (Pam3, 1$\mu$g mL$^{-1}$, 4hr) or primed and activated with nigericin (Nig, 10$\mu$M, 30min). Total cells were gated for singlets, intact cells, and ASC signal i.e. Height (H), Width (W) and Area (A). **b** Flow cytometric detection of polymerized ASC in Ctrl, *TRIM21*$^{KO}$, and *NLRP3*$^{KO}$ THP-1 cells left untreated, primed with Pam3 (1$\mu$g mL$^{-1}$, 4hr) or primed and activated with Nig (10$\mu$M, 30min). Histograms represent ASC height (H):area (A) ratio. Percent of increased H:A ASC signal represents activated cells, which is also presented as bar graph (bottom). **c** Flow cytometric detection of polymerized ASC particles released from untreated Ctrl THP-1 cells and Ctrl, *TRIM21*$^{KO}$, *ASC*$^{KO}$ and *NLRP3*$^{KO}$ THP-1 cells primed with LPS (0.5$\mu$g mL$^{-1}$, 2hr) and activated with Nig (10$\mu$M, 30min) and presented as number of particles. **d** Gating strategy for the flow cytometric detection of polymerized ASC particles released from untreated Ctrl THP-1 cells and Ctrl, *TRIM21*$^{KO}$, *ASC*$^{KO}$ and *NLRP3*$^{KO}$ THP-1 cells primed with LPS (0.5$\mu$g mL$^{-1}$, 2hr) and activated with Nig (10$\mu$M, 30min). This gating strategy was used for all samples in Fig. 4e, Supplementary Fig. 3c. Results **(b,c)** representative of n=3 experiments.

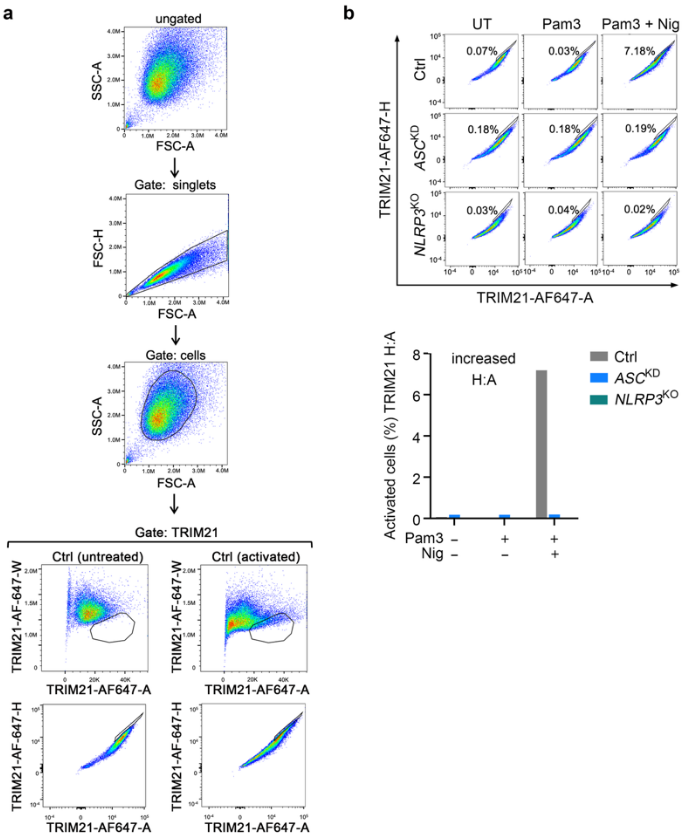


**Supplementary Fig. 4 | TRIM21 is co-released with polymerized ASC during inflammasome activation.**

**a** Gating strategy used for the flow cytometric detection of polymerized TRIM21 (Fig. 5a, Supplementary Fig. 4b) in Ctrl, *ASC*$^{KD}$, and *NLRP3*$^{KO}$ THP-1 cells, left untreated, primed with Pam3CSK4 (Pam3, 1μg mL$^{-1}$, 4hr) or primed and activated with nigericin (Nig, 10μM, 30min). Total cells were gated for singlets, intact cells, and TRIM21 signal i.e. height (H), Width (W) and Area (A). **b** Flow cytometric detection of polymerized TRIM21 in Ctrl, *ASC*$^{KD}$, and *NLRP3*$^{KO}$ THP-1 cells left untreated (UT), primed with Pam3 (1μg mL$^{-1}$, 4hr) or primed and activated with Nig (10μM, 30min). Histograms are presented as TRIM21 height (H):area (A) ratio. Percent of increased H:A TRIM21 signal represents activated cells, which is also presented as bar graph (bottom). Results **(b)** representative of n=3 experiments.

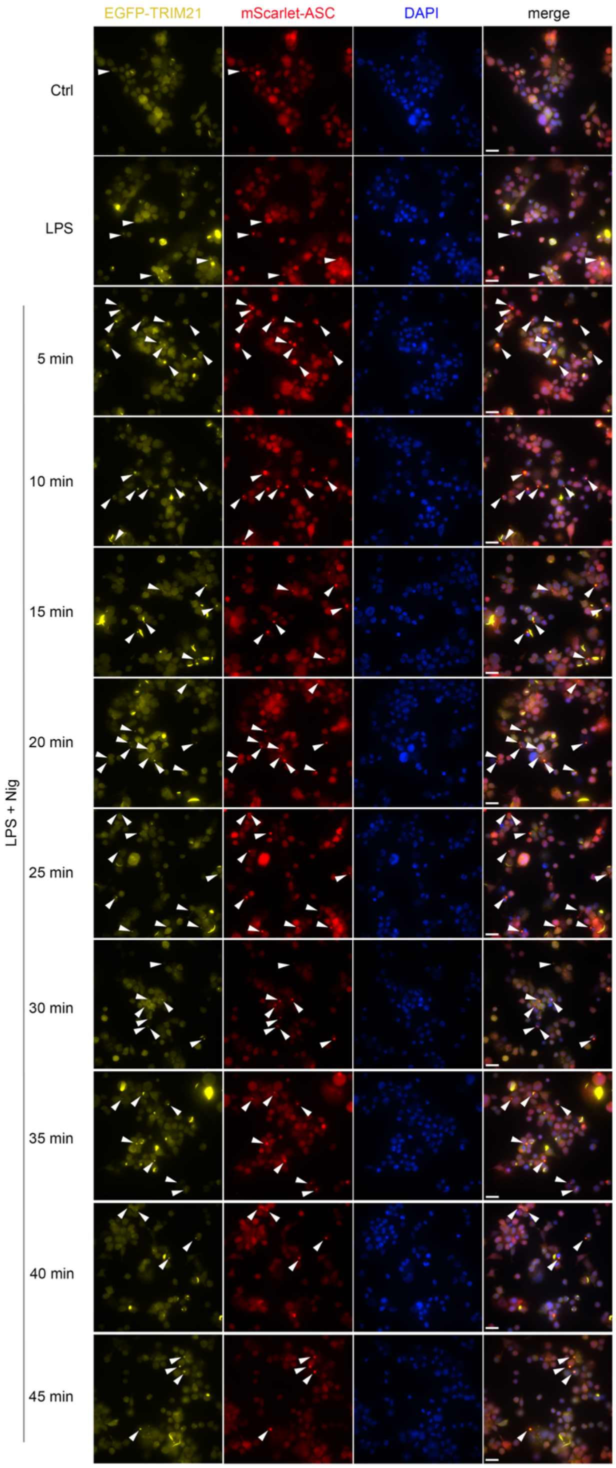


**Supplementary Fig. 5 | Microscopy time course of aggregated ASC and TRIM21 in cells.** Deconvolved fluorescence microscopy images of PMA-differentiated EGFP-TRIM21- and mScarlet-ASC-expressing THP-1 cells, left untreated, primed with LPS (0.5μg $mL^{-1}$, 2hr) or primed and activated with nigericin (Nig, 5μM, 5-45min) (untreated, LPS and LPS + 30min Nig shown in Fig. 5b). Nuclei (DNA) were stained with DAPI. Scale bar: 10 μm. Images representative of n=3 experiments.

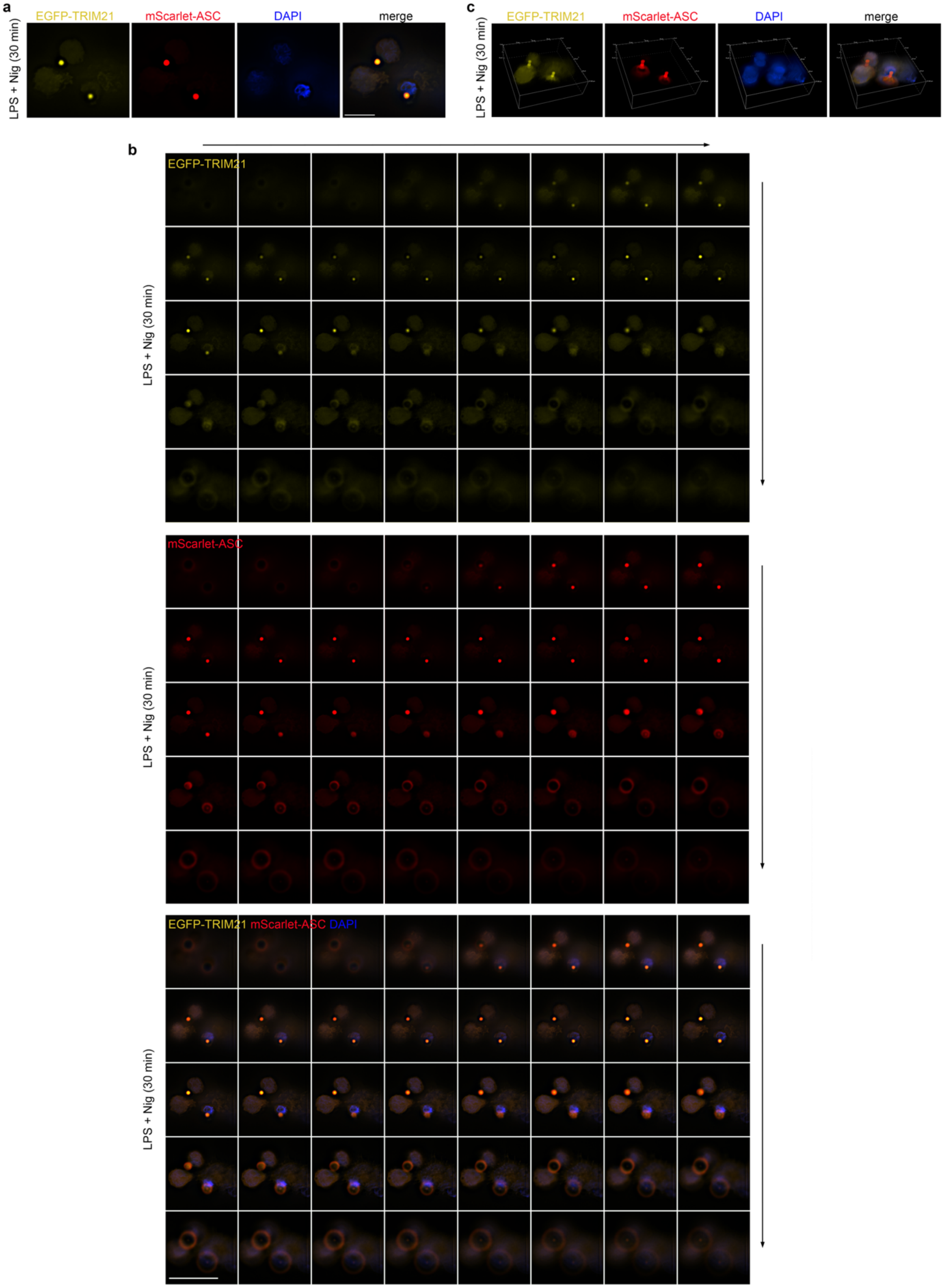


**Supplementary Fig. 6 | Tile and 3D view of aggregated ASC and TRIM21 in cells.**
**a-c** Deconvolved fluorescence microscopy images from z-series captured from PMA-differentiated EGFP-TRIM21- and mScarlet-ASC-expressing THP-1 cells primed with LPS (0.5μg $mL^{-1}$, 2hr) and

activated with nigericin (Nig, 5μM, 30min) as in Fig. 5b. Nuclei (DNA) were stained with DAPI. Shown is a magnified representative image of one focus plane. Scale bar: 5 μm **(a)**, tile view of all captured focus planes in 0.3μm increments, scale bar: 10 μm **(b)** and volumetric 3D view **(c)**. Scale bar: 10μm. Images representative of n=3 experiments.

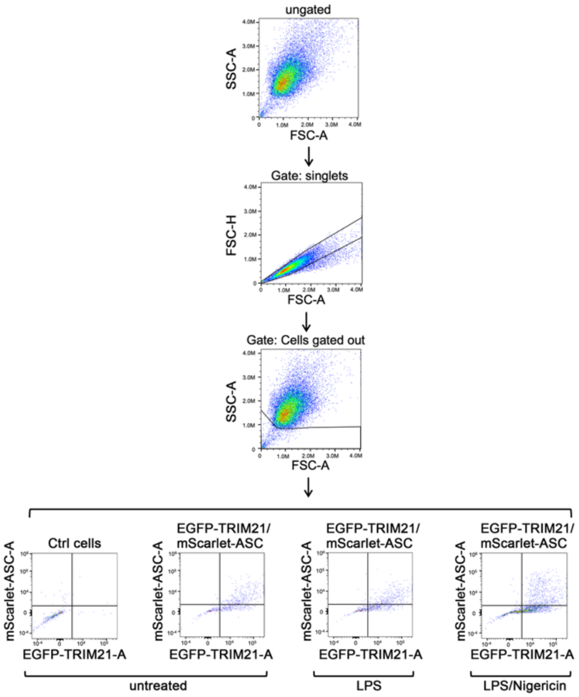


**Supplementary Fig. 7 | Gating strategy for the flow cytometric detection of polymerized $ASC^{+}TRIM21^{+}$ particles.**

Gating strategy used in Fig. 5j for the flow cytometric detection of polymerized mScarlet-$ASC^{+}$ and mScarlet-$ASC^{+}$EGFP-$TRIM21^{+}$ particles released from mScarlet-ASC- and EGFP-TRIM21-expressing THP-1 cells, left untreated, primed with LPS (0.5μg $mL^{-1}$, 2hr) or primed and activated with nigericin (Nig, 10μM, 30min). Total events were gated for singlets and cells gated out. Remaining events were gated for mScarlet-ASC and EGFP-TRIM21.

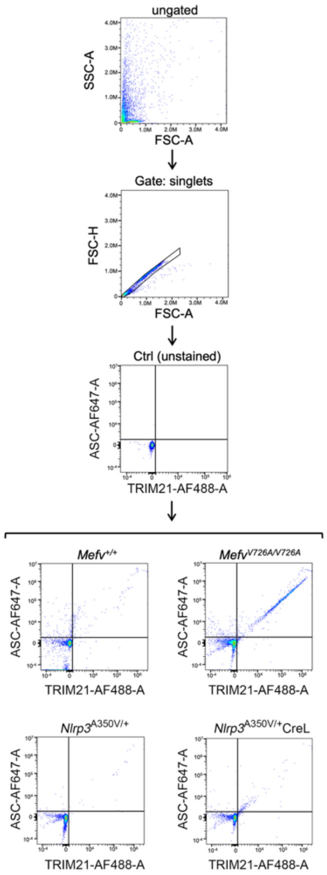


**Supplementary Fig. 8 | Gating strategy for the flow cytometric detection of polymerized ASC and TRIM21 in the serum of CAPS and FMF mice.**

Gating strategy used in Fig. 6c, d for the flow cytometric detection of polymerized $ASC^{+}$, $TRIM21^{+}$ and $ASC^{+}TRIM21^{+}$ particles in the serum of *Mefv*$^{+/+}$ and *Mefv*$^{V726A/V726A}$ mice, and of *Nlrp3*$^{A350V/+}$ and *Nlrp3*$^{A350V/+}$ CreL mice. Total events were gated for singlets and for ASC and TRIM21 signal.

## SUPPLEMENTARY TABLES

| Oligonucleotides | Sequence 5'-3'/Ref# | SOURCE |
|---|---|---|
| **Genotyping** | | |
| CreL-Common-Fwd | CTT GGG CTG CCA GAA TTT CTC | Integrated DNA Technologies |
| CreL-Wild-type-Rev | TTA CAG TCG GCC AGG CTG AC | Integrated DNA Technologies |
| CreL-Mutant-Rev | CCC AGA AAT GCC AGA TTA CG | Integrated DNA Technologies |
| Nlrp3-WT-Fwd | CCC TGC ATT TTG TTG TTG TTG | Integrated DNA Technologies |
| Nlrp3-WT-Rev | CCT GCT TCT CAC ATG TCG TC | Integrated DNA Technologies |
| Nlrp3-R350V Mutant-Fwd | GGG GAA CTT CCT GAC TAG GG | Integrated DNA Technologies |
| Nlrp3-R350V Mutant-Rev | CCT GCT TCT CAC ATG TCG TC | Integrated DNA Technologies |
| Mefv-WT-Fwd | CCG CTT TCT TCT GAT CCA AC | Integrated DNA Technologies |
| Mefv-WT-Rev | CCC AGG CAT CCT AAA CAC TG | Integrated DNA Technologies |
| Mefv-V726A Mutant-Fwd | GTC AAT CCG GGT GAA GAC AT | Integrated DNA Technologies |
| Mefv-V726A Mutant-Rev | CAC TTC CTT TGT GGC ATC | Integrated DNA Technologies |
| Trim21fl/+-SDL2 | GAA ACA TCT GTA CTG GCT CAG GCC | Integrated DNA Technologies |
| Trim21fl/+-RIPA8 | CAC CAG GCA CTG TTT ATG CTG C | Integrated DNA Technologies |
| Trim21-/-mice-9256 | CCT TGG CAT TAT TTG GGG GA | Integrated DNA Technologies |
| Trim21-/-mice-9257 | CTC CAT GCT TCA TGC AGT GC | Integrated DNA Technologies |
| Trim21-/-mice-9258 | GCG GAT CTT GAA GTT CAC CT | Integrated DNA Technologies |
| | | |
| **RT-qPCR** | | |
| Human ACTB-VIC | Hs99999903 | ThermoFisher |
| Human ASC/PYCARD-FAM | Hs00203118 | ThermoFisher |
| Human CASP1-FAM | Hs00354836 | ThermoFisher |
| Human IL18-FAM | Hs01038788 | ThermoFisher |
| Human IL1B-FAM | Hs01555410 | ThermoFisher |
| Human NLRP3-FAM | Hs00918082 | ThermoFisher |
| Human TNFA-FAM | Hs00174128 | ThermoFisher |
| Mouse ACTB-VIC | Mm04394036 | ThermoFisher |
| Mouse ASC/PYCARD-FAM | Mm00445747 | ThermoFisher |
| Mouse CASP1-FAM | Mm00438023 | ThermoFisher |
| Mouse IL18-FAM | Mm00434226 | ThermoFisher |
| Mouse IL1B-FAM | Mm00434228 | ThermoFisher |
| Mouse NLRP3-FAM | Mm00840904 | ThermoFisher |
| Mouse TNFA-FAM | Mm00443258 | ThermoFisher |
| | | |
| **Generation of TRIM21fl/+ mice** | | |
| P6 | GAG TGC ACC ATA TGG ACA TAT TGT C | Integrated DNA Technologies |
| T7 | CGA TAA GCC AGG TTA ACC TGC ATT A | Integrated DNA Technologies |
| N1 | TGC GAG GCC AGA GGC CAC TTG TGT AGC | Integrated DNA Technologies |
| N2 | TTC CTC GTG CTT TAC GGT ATC G | Integrated DNA Technologies |
| loxP | GTC GGC CAT CAC TGC AAA GAG | Integrated DNA Technologies |
| A2 | GTG ACT TTC CTA GTG CCC TGG AC | Integrated DNA Technologies |
| UNI | AGC GCA TCG CCT TCT ATC GCC TTC | Integrated DNA Technologies |
| SDL2 | GAA ACA TCT GTA CTG GCT CAG GCC | Integrated DNA Technologies |
| RIPA8 | CAC CAG GCA CTG TTT ATG CTG C | Integrated DNA Technologies |
| PB7 | CAG GAA ACG CAC TGC ATG AAG CAT | Integrated DNA Technologies |
| PB8 | AGC TCT TGC CTT CCA GTC TGT TCT | Integrated DNA Technologies |
| PB1 | CAT AAG ACC AAA GAC ACC CTC TTC C | Integrated DNA Technologies |

| | | |
|---|---|---|
| PB2 | GTG GAG CCT GAC AGT TGT GAA G | Integrated DNA Technologies |
| PB5 | CTT GCC ACC ACA GAG GAA CAT TGA C | Integrated DNA Technologies |
| PB6 | CCA TGG AGT GAG ATT GCA GCG G | Integrated DNA Technologies |
| | | |
| **Generation of TRIM21KO THP-1** | | |
| hTRIM21 gRNA#1 | ATG CTC ACA GGC TCC ACG AA | Integrated DNA Technologies |
| hTRIM21 gRNA#2 | ATG TTG GCT AGC TGT CGA TT | Integrated DNA Technologies |
| hTrim21gRNA1-Fwd | GTC TCC ACA CTG CTG TTT AAC G | Integrated DNA Technologies |
| hTrim21gRNA2-Rev | TTC CCA TCT TTC TCA CAG AAC A | Integrated DNA Technologies |
| | | |
| **Generation of Trim21KO iBMDMs** | | |
| mTrim21 gRNA#1 | TGG CCA CAT TCG ATA CTC AT | ThermoFisher |
| mTrim21 gRNA#2 | GTC TAT TGG GCC TGA GGT TT | ThermoFisher |
| mTrim21gRNA1-Fwd | GTC TCT GGA AAA GAT GTG GGA G | Integrated DNA Technologies |
| mTrim21gRNA1-Rev | GGC TAT ATG TCT ATT GGG CCT G | Integrated DNA Technologies |
| mTrim21gRNA2-Fwd | GAG GTC ACC TGT TCT ATC TGC C | Integrated DNA Technologies |
| mTrim21gRNA2-Rev | GAA GCT TCT CTC CAT GCT TCA T | Integrated DNA Technologies |

**Supplementary Table 1 | Oligonucleotides.** Nucleotide sequences and experimental applications of all oligonucleotides used.

| Patient# | HD/CAPS | NLRP3 Mutation |
|---|---|---|
| 1 | Healthy donor 1 | |
| 2 | Healthy donor 2 | |
| 3 | Healthy donor 3 | |
| 4 | Healthy donor 4 | |
| 5 | FCAS | L353P |
| 6 | FCAS | L353P |
| 7 | FCAS | A439V/G564R |
| 8 | FCAS/MWS | E525K/V198M |
| 9 | MWS/NOMID | R260W |
| 10 | NOMID | G326E |
| 11 | NOMID | F309Y |
| 12 | FCAS | L305P |
| 13 | FCAS/MWS | A439V |
| 14 | FCAS | L353P |
| 15 | FCAS | L353P |
| 16 | FCAS | L353P |
| 17 | FCAS | L353P |
| 18 | FCAS | A439V |

**Supplementary Table 2 | Human Subjects.** Summary of human plasma samples from healthy donors (HD) or patients with cryopyrin-associated autoinflammatory periodic syndrome (CAPS) and associated *Nlrp3* mutation used in this study. FCAS, familial cold autoinflammatory syndrome; MWS, Muckle-Wells syndrome; NOMID, neonatal onset multisystem inflammatory disease.

| REAGENT or RESOURCE | SOURCE | IDENTIFIER | RRID | Working concentration/dilution | Application |
|---|---|---|---|---|---|
| **Antibodies** | | | | | |
| Mouse monoclonal PE-conjugated anti-ASC, clone HASC-71 | BioLegend | Cat# 653903 | AB_2564507 | 50 ng mL-1 | Flow cytometry |
| Rabbit polyclonal anti-ASC, AL177 | Adipogen | Cat# AG-25B-0006-C100 | AB_2490440 | 1 µg mL-1/1:1,000/2.96 µg mL-1 | ELISA/Immunoblotting/PLA |
| Rabbit polyclonal antibody anti-ASC | Sigma-Aldrich | Cat# AB3607 | AB_2302813 | 1:1,000 | Immunoblotting |
| Mouse monoclonal antibody anti-ASC, clone B-3 | Santa-Cruz Biotechnology | Cat# sc-514414 | AB_2737351 | 400 ng mL-1/200 ng mL-1 | Flow cytometry/ELISA |
| Rabbit antibody anti-beta-tubulin | Cell Signaling Technology | Cat# 2146 | AB_2210545 | 1:1,000 | Immunoblotting |
| Mouse monoclonal antibody anti-cleaved caspase-1 (p20)/pro-caspase-1, clone Bally-1 | Adipogen | Cat# AG-20B-0048-C100 | AB_2490257 | 1:1,000 | Immunoblotting |
| Mouse monoclonal antibody anti-cleaved caspase-1 (p20)/pro-caspase-1, clone Casper-1 | Adipogen | Cat# AG-20B-0042-C100 | AB_2490248 | 1:1,000 | Immunoblotting |
| Mouse monoclonal anti-Flag, clone M2 | Sigma-Aldrich | Cat# F1804 | AB_262044 | 1 µg mL-1 | Immunostaining |
| Rabbit monoclonal antibody anti-GAPDH, clone 14C10 | Cell Signaling Technology | Cat# 2118 | AB_561053 | 1:1,000 | Immunoblotting |
| Mouse monoclonal antibody anti-GFP, clone B-2 | Santa-Cruz Biotechnology | Cat# sc-9996 | AB_627695 | 200 ng mL-1 | Epitope mapping (human) |
| Rabbit monoclonal antibody anti-GSDMD, clone L60 | Cell Signaling Technology | Cat# 93709 | AB_2800210 | 1:1,000 | Immunoblotting |
| Rabbit monoclonal antibody anti-cleaved/total GSDMD, EPR19828 | Abcam | Cat# ab209845 | AB_2783550 | 1:1,000 | Immunoblotting |
| Rabbit monoclonal antibody anti-cleaved GSDMD, clone E7H9G | Cell Signaling Technology | Cat# 36425 | AB_2799099 | 1:1,000 | Immunoblotting |
| Rat monoclonal anti-HA, clone 3F10 | Roche | Cat# 11867423001 | AB_390918 | 1 µg mL-1 | Immunostaining |
| Rat monoclonal anti-IL-18, clone 74 | MBL | Cat# MBL-D047-3 | AB_592016 | 1 µg mL-1 | ELISA |
| Rat monoclonal biotinylated anti-IL-18, clone 93-10C | MBL | Cat# MBL-D048-6 | AB_592012 | 1:2,000 | ELISA |
| Rabbit monoclonal anti-Myc, clone 71D10 | Cell Signaling Technology | Cat# 2278 | AB_490778 | 400 ng mL-1 | Immunostaining |
| Mouse monoclonal antibody anti-Myc, clone 9B11 | Cell Signaling Technology | Cat# 2276 | AB_331783 | 1:1,000 | Immunoblotting |
| Mouse monoclonal antibody anti-NLRP3, clone Cryo-2 | Adipogen | Cat# AG-20B-0014-C100 | AB_2490202 | 1:1,000 | Immunoblotting |
| Goat polyclonal anti-TRIM21 | Invitrogen | Cat# PA5-18147 | AB_10987024 | 1 µg mL-1/500 ng mL-1 | Flow cytometry/ELISA |
| Rabbit monoclonal anti-TRIM21, EPR20290 | Abcam | Cat# ab207728 | AB_2927717 | 606 ng mL-1 | ELISA/Immunoblotting |
| Rabbit polyclonal antibody anti-TRIM21 | Novus Biologicals | Cat# NBP3-03809 | AB_3532425 | 1 µg mL-1 | Flow cytometry |
| Rabbit polyclonal anti-TRIM21 | Invitrogen | Cat# PA5-120224 | AB_2913796 | 2.96 µg mL-1 | PLA |
| Donkey anti-goat, AlexaFluor-647-conjugated | Invitrogen | Cat# A-21447 | AB_141844 | 2 µg mL-1 | Flow cytometry |
| Donkey anti-mouse, AlexaFluor-647-conjugated | Invitrogen | Cat# A-31571 | AB_162542 | 2 µg mL-1 | Flow cytometry |
| Donkey anti-mouse, AlexaFluor-488-conjugated | Invitrogen | Cat# A-21202 | AB_141607 | 2 µg mL-1 | Flow cytometry/epitope mapping (mouse) |
| Donkey anti-rabbit, AlexaFluor-488-conjugated | Invitrogen | Cat# A-21206 | AB_2535792 | 2 µg mL-1 | Flow cytometry |
| Donkey anti-rabbit antibody, biotin-conjugated | Invitrogen | Cat# A-16039 | AB_2534713 | 750 ng mL-1 | ELISA |

| | | | | | |
|---|---|---|---|---|---|
| Goat HRP-conjugated anti-rabbit IgG (H+L) | Cell Signaling Technology | Cat# 7074 | AB_2099233 | 1:5,000 | Immunoblotting |
| Horse HRP-conjugated anti-mouse IgG (H+L) | Cell Signaling Technology | Cat# 7076 | AB_330924 | 1:5,000 | Immunoblotting |
| Rabbit IgG Isotype control | Invitrogen | Cat# 02-6102 | AB_2532938 | 1μg | Immunoprecipitation |

| **Chemicals, peptides, and recombinant proteins** | | |
|---|---|---|
| Acetone | Fisher | Cat# A929-1 |
| AlexaFluor-647-conjugated streptavidin | Invitrogen | Cat# S21374 |
| ATP | Sigma-Aldrich | Cat# A6419 |
| AutoMACS Buffer | Miltenyi Biotec | Cat# 130-091-221 |
| Avidin-HRP | Invitrogen | Cat# 18-4200-89 |
| Bacillus anthracis lethal factor | List labs | Cat# 104 |
| Biotinylated pan-caspase inhibitor, biotin-YVAD-CMK | Anaspec | Cat# AS-60841 |
| Bovine Serum Albumin, BSA | Fisher | Cat# BP1605-100 |
| Bromophenol blue | IBI Scientific | Cat# IB74040 |
| Chloroform | Sigma-Aldrich | Cat# C2432-500ML |
| Calcium Pyrophosphate Dihydrate (CPPD) crystals | Invivogen | Cat# Tlrl-cppd |
| Cytofix/Cytoperm | BD Biosciences | Cat# 554714 |
| Disuccinimidyl suberate, DSS | Thermo Scientific | Cat# A39267 |
| Disulfiram (Tetraethylthiuram disulfide), DSF | Millipore-Sigma | Cat#86720-50G |
| Dithiothreitol, DTT | GoldBio | Cat# DTT100 |
| DMEM high glucose | Corning | Cat# 10-013-CV |
| Donkey serum | Fisher | Cat# NC9719162 |
| ELISA/ELISPOT diluent | Invitrogen | Cat# 00-4202-56 |
| Ethylenediamine tetraacetic acid, EDTA | Fisher | Cat# S311-500 |
| Fetal bovine serum, FBS | Thermo Scientific | Cat# 16140071 |
| Flagellin (from *Salmonella* enterica subsp. enterica serovar Typhimurium) | Invivogen | Cat# Tlrl-epstfla-5 |
| Glycerol | Thermo Scientific | Cat# AC158920100 |
| Goat serum | Fisher | Cat# NC9660079 |
| HEPES buffer | Corning | Cat# 25-060-Cl |
| Human TruStain Fc Receptor Block | Biolegend | Cat# 422302 |
| Hydrochloric acid, HCl | Sigma-Aldrich | Cat# 258148-500ML |
| Imidazole | Alfa Aesar | Cat# A10221 |
| Isopropanol | Sigma-Aldrich | Cat# 190764-4L |
| Lethal toxin, LeTx (*Bacillus anthracis* Lethal Factor) | List Biological Laboratories | Cat# 172L |
| Lethal toxin, LeTx (*Bacillus anthracis* Protective Antigen) | List Biological Laboratories | Cat# 171E |
| Lipofectamine 2000 | Invitrogen | Cat# 11668019 |
| Lipofectamine RNAi/MAX | Invitrogen | Cat#13778150 |
| LPS *E. coli* O111:B4 | Sigma-Aldrich | Cat# L2630-100MG |
| LPS *E. coli* O111:B4 | Sigma-Aldrich | Cat# LPS25 |
| Magnesium chloride, MgCl2 | Sigma-Aldrich | Cat# M8266-1KG |
| M-CSF | Gibco | Cat# 315-02-10UG |
| Ni-NTA magnetic beads | Thermo Scientific | Cat# 88832 |
| Nigericin | Invivogen | Cat# Tlrl-nig |
| Non-fat dry milk | Fisher | Cat# NC9952266 |
| Non-targeting stealth control siRNAs | Invitrogen | Cat# 12935300 |
| NP-40, Igepal CA+A72-630 | Sigma-Aldrich | Cat# 13021-500ML |
| Opti-MEM | Gibco | Cat# 31985088 |
| Pam3csk4 | Invivogen | Cat# tlrl-pms |
| Paraformaldehyde | Electron Microscopy Sciences | Cat# 15713S |
| Phosphate buffer saline, PBS buffer | Corning | Cat# 21-031-CV |
| Penicillin and Streptomycin | Gibco | Cat# 15140122 |
| Perm/Wash buffer | BD Biosciences | Cat# 554723 |

| | | | |
|---|---|---|---|
| Phenylmethylsulfonyl fluoride, PMSF | Santa-Cruz Biotechnology | Cat# sc-3597 | |
| Phorbol 12-myristate-13-acetate, PMA | Invitrogen | Cat# J63916.MCR | |
| Polybrene | Selleck Chemical | Cat# 50-313-4437 | |
| poly(dA:dT) | Invivogen | Cat# Tlrl-patn | |
| Polymyxin B | Invivogen | Cat# tlrl-pmb | |
| Potassium chloride, KCl | EMD | Cat# 7300-500G | |
| Prolong glass antifade with Nucblue stain | Invitrogen | Cat# P36981 | |
| Protease inhibitor cocktail | Thermo Scientific | Cat# A32963 | |
| Protein A/G agarose beads | Santa-Cruz Biotechnology | Cat# sc-2003 | |
| Protein A/G magnetic beads | Thermo Scientific | Cat# 88802 | |
| PVDF membranes | Millipore Sigma | Cat# IPVH00010 | |
| PYR-41, E1 enzyme inhibitor | Millipore Sigma | Cat# 622105 | |
| RPMI 1640 | Cytiva Hyclone, Fisher Scientific | Cat# SH30027FS | |
| Sodium dodecyl sulfate, SDS | Invitrogen | Cat# 15525017 | |
| Silica/NanoSiO2 crystals | Invivogen | Cat# Tlrl-SiO-2 | |
| Sodium chloride, NaCl | Fisher | Cat# S271-10 | |
| Sodium deoxycholate | Alfa Aesar | Cat# J62288 | |
| Sodium fluoride | Sigma | Cat# S-7920 | |
| Sodium orthovanadate | Sigma | Cat# S6508-50G | |
| Sodium pyrophosphate | Sigma-Aldrich | Cat# 221368-500G | |
| Stealth siRNA, target hTRIM21 | Invitrogen | Cat# 1299001 | |
| Sulfuric acid | Sigma-Aldrich | Cat# 258105-500ML | |
| Super Signal West Femto ECL | Thermo Scientific | Cat# 34096 | |
| Clostridioides difficile Toxin B ,TcdB | Cytoskeleton | Cat# CT04 | |
| Tetramethylbenzidine, TMB | Invitrogen | Cat# 00-4201-56 | |
| Trichloroacetic acid, TCA | VWR | Cat# BDH7372-2 | |
| Tris Base | Fisher | Cat# BP152-10 | |
| Triton X-100 | EMD | Cat# 9410 | |
| Trizol | Thermo Scientific | Cat# 15596026 | |
| Tween 20 | Thermo Scientific | Cat# J20605.AP | |
| Ultra-pure LPS *E. coli* O111:B4 | Invivogen | Cat# Tlrl-3pelps | |
| Virofect | Targeting Systems | Cat #007 | |
| XenoLight Rediject Inflammation probe | Perkin Elmer | Cat# 760536 | |
| | | | |
| **Critical commercial assays** | | | |
| IL-1b ELISA (human) | Invitrogen | Cat# 88-7261-22 | |
| IL-1b ELISA (mouse) | Invitrogen | Cat# 88-7013-22 | |
| IL-6 ELISA (human) | BD Biosciences | Cat# 555220 | |
| IL-18 ELISA (human) | R&D Systems | Cat# DY318-05 | |
| TNF ELISA (human) | Invitrogen | Cat# 887346-76 | |
| TNF ELISA (mouse) | Invitrogen | Cat# 88-7324-88 | |
| Anti-TRIM21 (Ro52) IgG (human) | AssayPro | Cat# ER7501-1 | |
| Anti-TRIM21 IgG (mouse) | Alpha Diagnostic | Cat# 5730 | |
| MycoStrip Mycoplasma Detection Kit | Invivogen | Cat# rep-mysnc-100 | |
| CyQUANT LDH Cytotoxicity Assay kit | Invitrogen | Cat# C203021 | |
| Verso cDNA Synthesis Kit | Thermo Scientific | Cat# AB1453A | |
| Neon™ Transfection System | Invitrogen | Cat# MPK10096 | |
| Verso cDNA synthesis kit | Thermo Scientific | Cat# AB1453B | |
| Duolink® In Situ Red Starter Kit Mouse/Rabbit (PLA) | Sigma-Aldrich | Cat# DUO92101-1KT | |
| **Experimental models: Cell lines** | | | |
| AMJ2-C11 | American Type Culture Collection | Cat# CRL-2456 | CVCL_5913 |
| ASCKD THP-1 | https://doi.org/10.4049/jimmunol.0802367 | | |
| ASCKO THP-1 | Invivogen | Cat# thp-koascz | |

| | | | |
|---|---|---|---|
| CASP1KO THP-1 | https://doi.org/10.1038/s41467-018-03409-3 | | |
| GFP-TRIM21/mScarlet-ASC THP-1 | This manuscript | | |
| HEK293 | American Type Culture Collection | Cat# CRL-3216 | CVCL_0063 |
| HEK293T Lenti-X | Takara Bio | Cat# 632180 | |
| NLRP3KO iBMDMs | This manuscript | | |
| NLRP3KO THP-1 | https://doi.org/10.1038/s41467-018-03409-3 | | |
| TRIM21KO + GFP-TRIM21WT THP-1 | This manuscript | | |
| TRIM21KO + GFP-TRIM21ΔRING THP-1 | This manuscript | | |
| TRIM21KO + GFP-TRIM21ΔBBOX/CC THP-1 | This manuscript | | |
| TRIM21KO + GFP-TRIM21ΔPRY/SPRY THP-1 | This manuscript | | |
| Trim21KO iBMDMs | This manuscript | | |
| TRIM21KO THP-1 | This manuscript | | |
| WT Cas9-GFP iBMDMs | This manuscript | | |
| WT iBMDMs | This manuscript | | |
| WT THP-1 | American Type Culture Collection | Cat# TIB-202 | CVCL_0006 |
| **Experimental models: Animals** | | | |
| B6.129-Nlrp3tm1Hhf/J (Nlrp3A350V) | Provided by H.M.H. | Available at The Jackson Laboratory, strain# 017969 | IMSR_JAX:017969 |
| B6.129P2-Lyz2tm1(cre)Ifo/J | The Jackson Laboratory | Strain# 004781 | IMSR_JAX:004781 |
| C57BL/6-Trim21tm1Hm/J (Trim21-/-) | The Jackson Laboratory | Strain# 010724 | IMSR_JAX:010724 |
| C57BL/6J | The Jackson Laboratory | Strain# 000664 | IMSR_JAX:000664 |
| MefvV726A | Provided by D.L.K and J.J.C. | https://doi.org/10.1016/j.immuni.2011.02.020 | |
| Nlrp3–/– | Provided by Vishva Dixit (Genentech) | | |
| Nlrp3D301N | Provided by H.M.H. | Available at The Jackson Laboratory, strain# 017971 | IMSR_JAX:017971 |
| Trim21fl/+ | This manuscript | | |
| **Plasmids** | | | |
| lentiCRISPRv1 | Addgene | Cat# 49535 | Addgene_49535 |
| pMD.2G | Addgene | Cat# 12259 | Addgene_12259 |
| pRK5-HA-Ubiquitin-K11 | Addgene | Cat# 22901 | Addgene_22901 |
| pRK5-HA-Ubiquitin-K27 | Addgene | Cat# 22902 | Addgene_22902 |
| pRK5-HA-Ubiquitin-K29 | Addgene | Cat# 22903 | Addgene_22903 |
| pRK5-HA-Ubiquitin-K33 | Addgene | Cat# 17607 | Addgene_17607 |
| pRK5-HA-Ubiquitin-K48 | Addgene | Cat# 17605 | Addgene_17605 |
| pRK5-HA-Ubiquitin-K6 | Addgene | Cat# 22900 | Addgene_22900 |
| pRK5-HA-Ubiquitin-K63 | Addgene | Cat# 17606 | Addgene_17606 |
| pRK5-HA-Ubiquitin-KO | Addgene | Cat# 17603 | Addgene_17603 |
| pRK5-HA-Ubiquitin-WT | Addgene | Cat# 17608 | Addgene_17608 |
| pSP72 | Promega | Cat# P2191 | |
| psPAX2 | Addgene | Cat# 12260 | Addgene_12260 |

**Supplementary Table 3 | Key Resources.** Detailed information on reagents, suppliers, and identifiers used in experimental procedures.
