# Supplementary material for: TRIM21 facilitates inflammasome assembly and contributes to autoinflammatory disease
Source: Nat Commun. 2026 May 22;17:6726. doi: 10.1038/s41467-026-73350-3 (PMC13385917; doi:10.1038/s41467-026-73350-3)
Supplement: Supplementary file 3 — Reporting Summary [file 41467_2026_73350_MOESM3_ESM.pdf]

nature portfolio

Corresponding author(s): Christian Stehlik
Last updated by author(s): Mar 24, 2026

Reporting Summary

Nature Portfolio wishes to improve the reproducibility of the work that we publish. This form provides structure for consistency and transparency in reporting. For further information on Nature Portfolio policies, see our Editorial Policies and the Editorial Policy Checklist.

Statistics

For all statistical analyses, confirm that the following items are present in the figure legend, table legend, main text, or Methods section.

n/a	Confirmed	
☐	☒	The exact sample size (*n*) for each experimental group/condition, given as a discrete number and unit of measurement
☒	☐	A statement on whether measurements were taken from distinct samples or whether the same sample was measured repeatedly
☐	☒	The statistical test(s) used AND whether they are one- or two-sided<br>*Only common tests should be described solely by name; describe more complex techniques in the Methods section.*
☒	☐	A description of all covariates tested
☒	☐	A description of any assumptions or corrections, such as tests of normality and adjustment for multiple comparisons
☐	☒	A full description of the statistical parameters including central tendency (e.g. means) or other basic estimates (e.g. regression coefficient) AND variation (e.g. standard deviation) or associated estimates of uncertainty (e.g. confidence intervals)
☐	☒	For null hypothesis testing, the test statistic (e.g. *F*, *t*, *r*) with confidence intervals, effect sizes, degrees of freedom and *P* value noted<br>*Give P values as exact values whenever suitable.*
☒	☐	For Bayesian analysis, information on the choice of priors and Markov chain Monte Carlo settings
☒	☐	For hierarchical and complex designs, identification of the appropriate level for tests and full reporting of outcomes
☐	☒	Estimates of effect sizes (e.g. Cohen's *d*, Pearson's *r*), indicating how they were calculated

Our web collection on statistics for biologists contains articles on many of the points above.

Software and code

Policy information about availability of computer code

Data collection	Nikon NIS Elements Advanced Research 5.2, BD FACS Sortware, SKanIT 6.0.2, iBright FL1500 software version 1.7.0, Applied Biosystems QuantStudio3 1.3.3, PerkinElmer Living Image, SpectroFlo 3.1.0.
Data analysis	Adobe Photoshop 2022, Adobe Illustrator 2022, FlowJo 10, Prism Graphpad 10

For manuscripts utilizing custom algorithms or software that are central to the research but not yet described in published literature, software must be made available to editors and reviewers. We strongly encourage code deposition in a community repository (e.g. GitHub). See the Nature Portfolio guidelines for submitting code & software for further information.

Data

Policy information about availability of data

All manuscripts must include a data availability statement. This statement should provide the following information, where applicable:

- Accession codes, unique identifiers, or web links for publicly available datasets
- A description of any restrictions on data availability
- For clinical datasets or third party data, please ensure that the statement adheres to our policy

Data supporting the findings of this study are available within the article and its Supplementary Figures. The source data underlying Figures 1-6, Extended Data Figures 2-5 are provided as a Source Data file.

Research involving human participants, their data, or biological material

Policy information about studies with human participants or human data. See also policy information about sex, gender (identity/presentation), and sexual orientation and race, ethnicity and racism.

Reporting on sex and gender	Our available patient cohort for this rare disease was too small to perform analyses based on sex.
Reporting on race, ethnicity, or other socially relevant groupings	Our available patient cohort for this rare disease was too small to perform analyses based on race, ethnicity, or other socially relevant groupings and this information is also unknown to the research team.
Population characteristics	healthy and Cryopyrinopathy patients
Recruitment	recruited during routine clinical care
Ethics oversight	University of California San Diego

Note that full information on the approval of the study protocol must also be provided in the manuscript.

Field-specific reporting

Please select the one below that is the best fit for your research. If you are not sure, read the appropriate sections before making your selection.

[x] Life sciences [ ] Behavioural & social sciences [ ] Ecological, evolutionary & environmental sciences

For a reference copy of the document with all sections, see nature.com/documents/nr-reporting-summary-flat.pdf

Life sciences study design

All studies must disclose on these points even when the disclosure is negative.

Sample size	no pre-determination was performed, internal controls in each experiment with well known effect were used and sample size was based on prior experience or available human subjects
Data exclusions	no data were excluded
Replication	we replicated some data at 2 different institutes; asked multiple researchers to replicate key data; used complementary approaches for testing: for example, we used siRNA and CRISPR/Cas9 knock out to validate each approach. After pilot optimization, all representative results have been independently repeated at least 3 times with similar results and all “n” refer to biological replicates.
Randomization	we randomly assigned animals and cells to different treatment groups.
Blinding	no on purpose blinding was used, but we separated sample collection and data collection when possible and appropriate, except for mouse studies, where investigators were blinded.

Reporting for specific materials, systems and methods

We require information from authors about some types of materials, experimental systems and methods used in many studies. Here, indicate whether each material, system or method listed is relevant to your study. If you are not sure if a list item applies to your research, read the appropriate section before selecting a response.

Materials & experimental systems

n/a	Involved in the study	
[ ]	[x]	Antibodies
[ ]	[x]	Eukaryotic cell lines
[x]	[ ]	Palaeontology and archaeology
[ ]	[x]	Animals and other organisms
[x]	[ ]	Clinical data
[x]	[ ]	Dual use research of concern
[x]	[ ]	Plants

Methods

n/a	Involved in the study	
[x]	[ ]	ChIP-seq
[ ]	[x]	Flow cytometry
[x]	[ ]	MRI-based neuroimaging

Antibodies

Antibodies used	Mouse monoclonal PE-conjugated anti-ASC, clone HASC-71 (BioLegend, Cat# 653903)<br>Goat polyclonal anti-TRIM21 (Invitrogen, Cat# PA5-18147)<br>Rat monoclonal anti-IL-18, clone 74 (MBL, Cat# MBL-D047-3)

Rat monoclonal biotinylated anti-IL-18, clone 93-10C (MBL, Cat# MBL-D048-6)
Rabbit polyclonal anti-ASC, AL177 (Adipogen, Cat# AG-25B-0006-C100)
Rabbit monoclonal anti-TRIM21, EPR20290 (Abcam, Cat# ab207728)
Rabbit monoclonal anti-Myc, clone 71D10 (Cell Signaling Technology, Cat# 2278)
Rat monoclonal anti-HA, clone 3F10 (Roche, Cat# 11867423001)
Mouse monoclonal anti-Flag, clone M2 (Sigma-Aldrich, Cat# F1804)
Rabbit polyclonal antibody anti-ASC (Sigma-Aldrich,Cat# AB3607)
Mouse monoclonal antibody anti-ASC, clone B-3 (Santa-Cruz Biotechnology, Cat# sc-514414)
Mouse monoclonal antibody anti-NLRP3, clone Cryo-2 (Adipogen, Cat# AG-20B-0014-C100)
Mouse monoclonal antibody anti-cleaved caspase-1 (p20)/pro-caspase-1, clone Bally-1 (Adipogen, Cat# AG-20B-0048-C100)
Mouse monoclonal antibody anti-cleaved caspase-1 (p20)/pro-caspase-1, clone Casper-1 (Adipogen, Cat# AG-20B-0042-C100)
Mouse monoclonal antibody anti-Beta-tubulin, clone AA4.3 (DHSB, Cat# AA4.3)
Rabbit monoclonal antibody anti-cleaved GSDMD, clone E7H9G (Cell Signaling Technology, Cat# 36425)
Rabbit monoclonal antibody anti-GSDMD, clone L60 (Cell Signaling Technology, Cat# 93709)
Rabbit monoclonal antibody anti-cleaved/total GSDMD, EPR19828 (Abcam, Cat# ab209845)
Mouse monoclonal antibody anti-Myc, clone 9B11 (Cell Signaling Technology, Cat# 2276)
Mouse monoclonal antibody anti-GFP, clone B-2 (Santa-Cruz Biotechnology, Cat# sc-9996)
Rabbit monoclonal antibody anti-GAPDH, clone 14C10 (Cell Signaling Technology, Cat# 2118)
Goat HRP-conjugated anti-rabbit IgG (H+L) (Cell Signaling Technology, Cat# 7074)
Horse HRP-conjugated anti-mouse IgG (H+L) (Cell Signaling Technology, Cat# 7076)
Donkey anti-goat, AlexaFluor-647-conjugated (Invitrogen, Cat# A-21447)
Donkey anti-mouse, AlexaFluor-647-conjugated (Invitrogen, Cat# A-31571)
Donkey anti-rabbit, AlexaFluor-488-conjugated (Invitrogen, Cat# A-21206)
Donkey anti-rabbit antibody, biotin-conjugated (Invitrogen, Cat# A-16039)
Rat Anti-Mouse CD16/CD32, clone 93 (Mouse Fc Block, Biolegend, Cat# 101302)

Validation

except well established antibodies (tubulin) we tested antibodies using one of these approaches: epitope tag antibodies: using transient transfection of tagged cDNAs and controls and western blot analysis. Antibodies to inflammasome components were tested in cells with shRNA knock-down, CRISPR/Cas9 knock-out, or knock-out BMDM, as well as using resting cells and cells with active inflammasome for testing cleaved caspase-1 and GSDMD, release of inflammasome components as well as ELISA assays. Where appropriate, we included knock-out cells as controls in experiments.

Eukaryotic cell lines

Policy information about cell lines and Sex and Gender in Research

Cell line source(s)

HEK293T (ATCC, CRL-3216): female origin;
Lenti-X HEK293 (Takara Bio, 632180) female origin;
THP-1 (ATCC, TIB-202) and derivatives, ASC KO THP-1 (Invivogen, thp-koascz): male origin

Authentication

cell lines were directly obtained from the vendor or ATCC and those with a known genotype (stable expressing cells, knock out cells), were routinely tested by western blot for validation.

Mycoplasma contamination

we routinely test cell lines for Mycoplasma contamination, a statement is included in the methods section. Cells used for experiments tested negative for Mycoplasma.

Commonly misidentified lines
(See ICLAC register)

cell lines used in this study are not commonly misidentified and are not included in this database.

Animals and other research organisms

Policy information about studies involving animals; ARRIVE guidelines recommended for reporting animal research, and Sex and Gender in Research

Laboratory animals

Trim21fl/+ (generated by Ingenious Targeting Laboratory)
B6.129P2-Lyz2tm1(cre)Ifo/J (The Jackson Laboratory)
C57BL/6J (WT) (The Jackson Laboratory)
Nlrp3-/- (Genentech):
Trim21-/- (The Jackson Laboratory)
Nlrp3A350V (University of California San Diego)
Nlrp3D301N (University of California San Diego)
MefvV726A (National Institutes of Health, NIH)
all 12-16 weeks of age

Wild animals

N/A

Reporting on sex

sex-based analysis is included in some experiments

Field-collected samples

N/A

Ethics oversight

Cedars-Sinai Medical Center and University of California San Diego Institutional Animal Care and Use Committees (IACUC)

Note that full information on the approval of the study protocol must also be provided in the manuscript.

Plants

Seed stocks	N/A
Novel plant genotypes	N/A
Authentication	N/A

Flow Cytometry

Plots

Confirm that:

- [x] The axis labels state the marker and fluorochrome used (e.g. CD4-FITC).
- [x] The axis scales are clearly visible. Include numbers along axes only for bottom left plot of group (a 'group' is an analysis of identical markers).
- [x] All plots are contour plots with outliers or pseudocolor plots.
- [x] A numerical value for number of cells or percentage (with statistics) is provided.

Methodology

Sample preparation	Human and mouse cells as well as mouse serum samples were used for flow cytometry analysis. Cells were fixed, permeabilized and stained with primary antibodies followed by staining with a secondary antibody conjugated with the indicated fluorophores. After staining, samples were washed, strained and analyzed by flow cytometry.
Instrument	Flow cytometry data was collected using a Cytek Aurora NL-3000 cytometer. Cell sorting was performed using a BD Influx instrument
Software	Flow cytometry data was collected using the SpectroFlo 3.1.0 software and analyzed using FlowJo 10 software. Cell sorting data was acquired using BD FACS Sortware.
Cell population abundance	The final populations were 0.5-30%.
Gating strategy	All acquired events were gated for singlets then, depending on the experiment, gated on intact cells or cells gated out or populations positive for the indicated fluorophores.

- [x] Tick this box to confirm that a figure exemplifying the gating strategy is provided in the Supplementary Information.
