# Supplementary material for: TRIM21 facilitates inflammasome assembly and contributes to autoinflammatory disease
Source: Nat Commun. 2026 May 22;17:6726. doi: 10.1038/s41467-026-73350-3 (PMC13385917; doi:10.1038/s41467-026-73350-3)

Figure 1c

AP: Ni^{2+}
WB: ASC

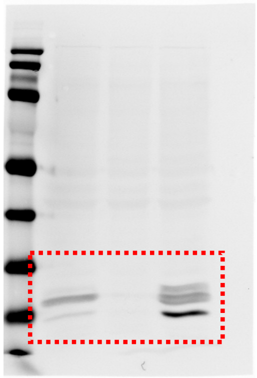

AP: Ni^{2+}
WB: TRIM21

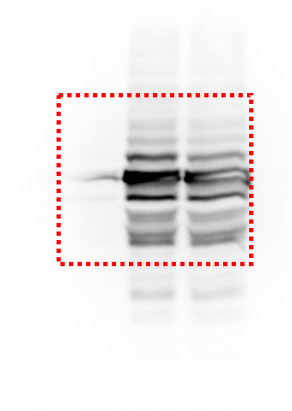

TCL:
WB: ASC

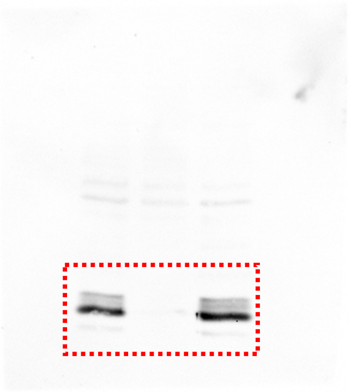

TCL:
WB: TRIM21

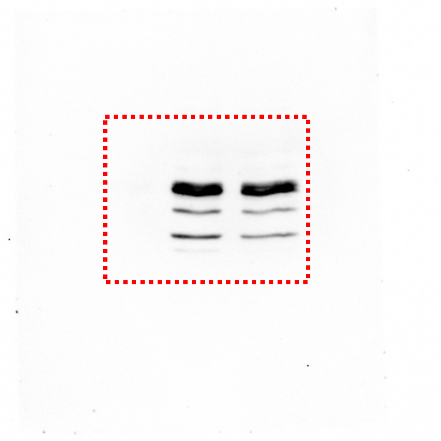

Figure 1d

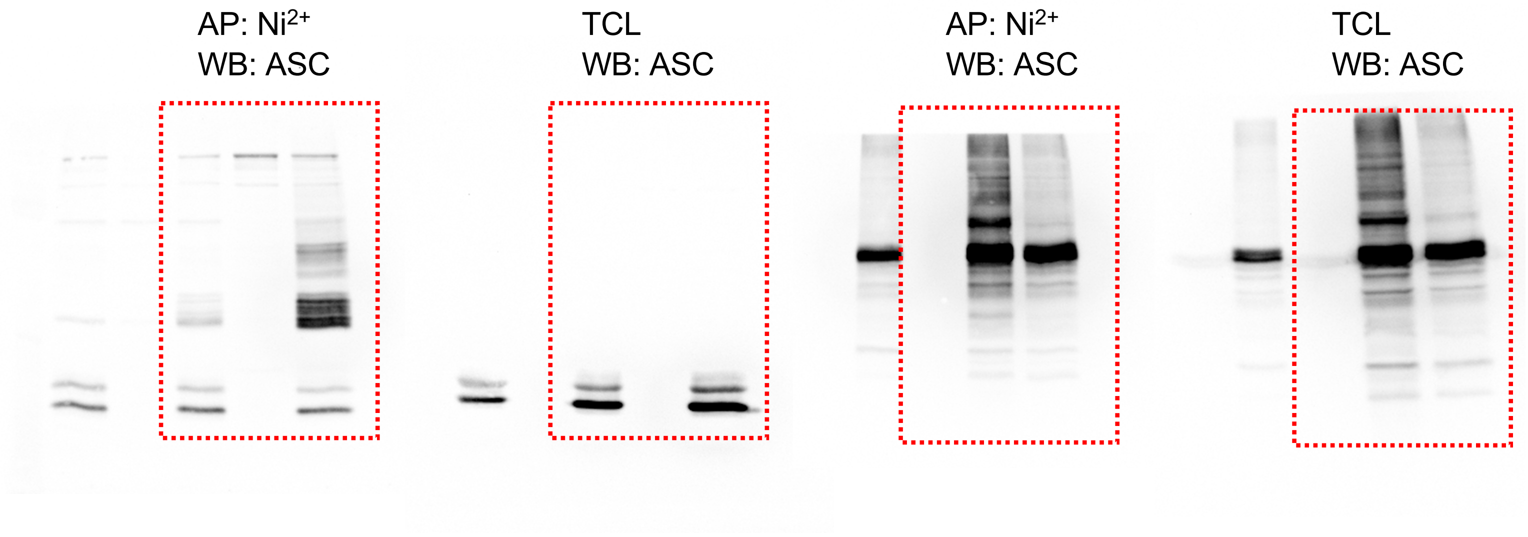

Figure 1e

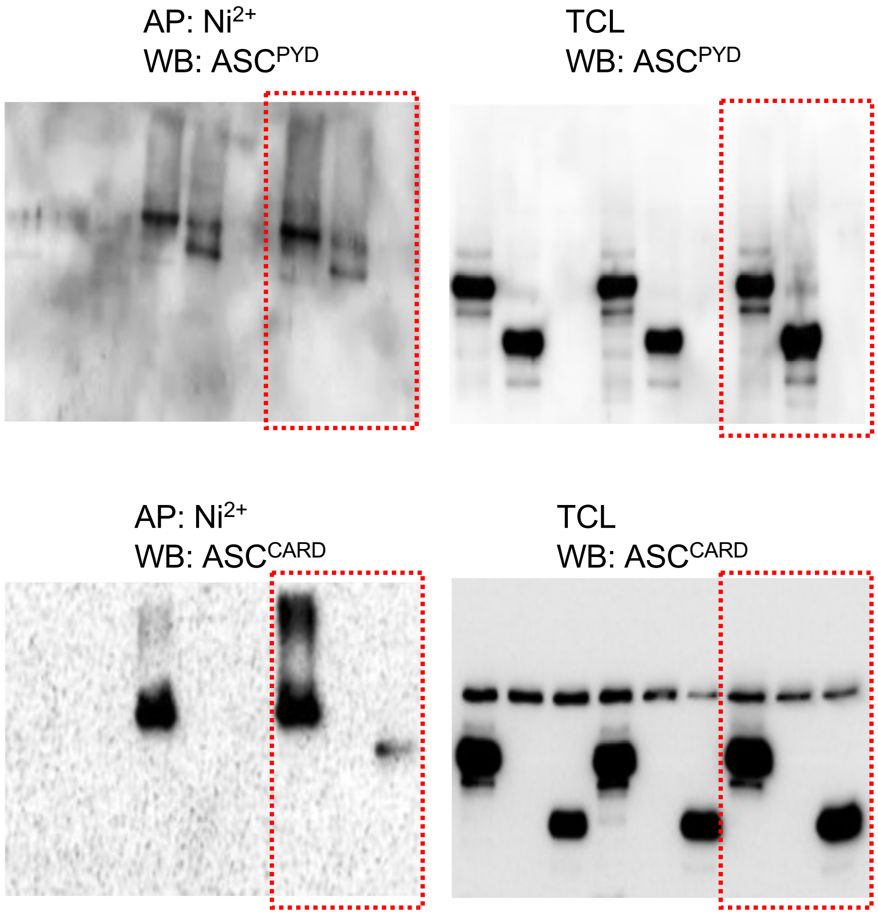

Figure 1f

WB: ASC

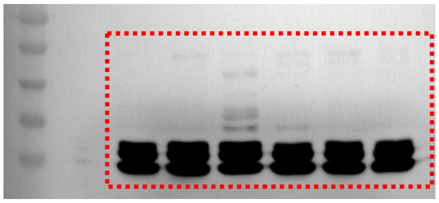

Figure 1g

WB: ASC

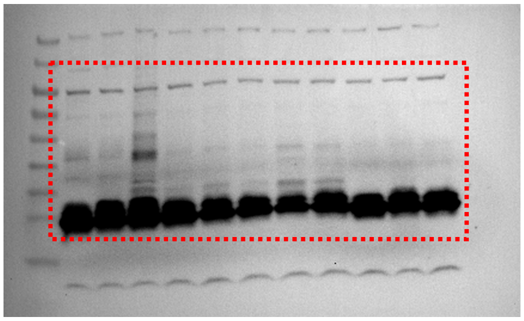

Figure 1h

IP: ASC
WB: Ubiquitin

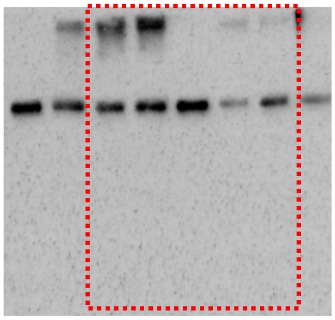

IP: ASC
WB: ASC

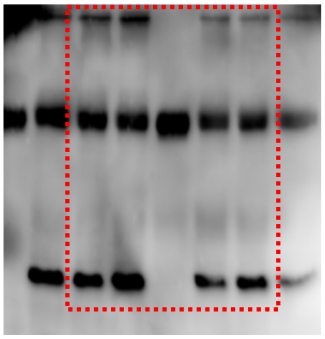

Figure 2c

SN: WB: cleaved Casp1

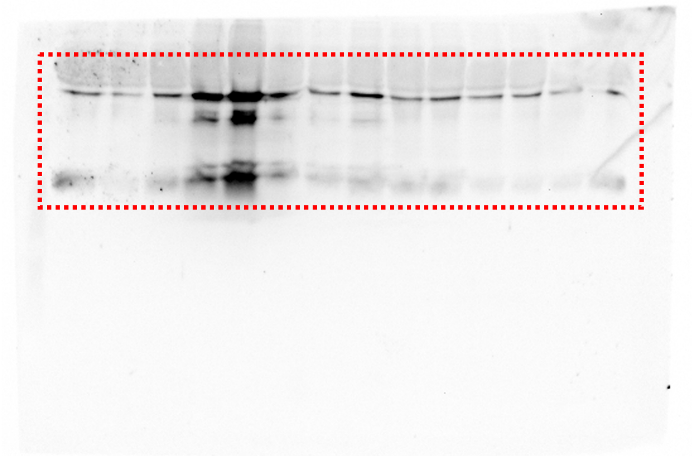

TCL: WB: Casp1

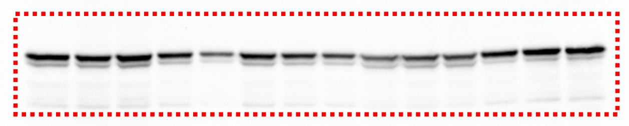

TCL: WB: cleaved GSDMD-N

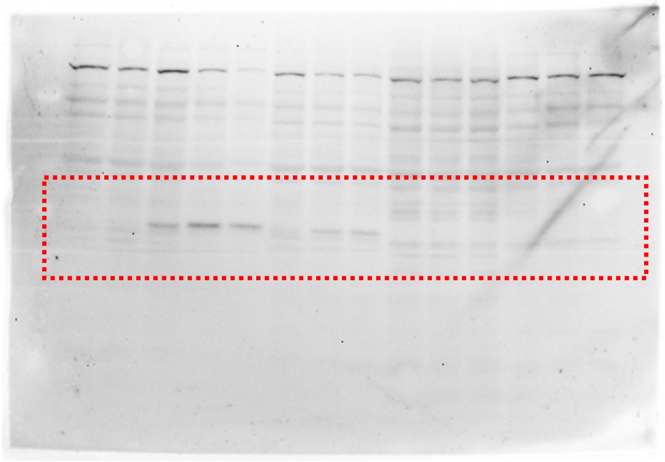

TCL: WB: GSDMD

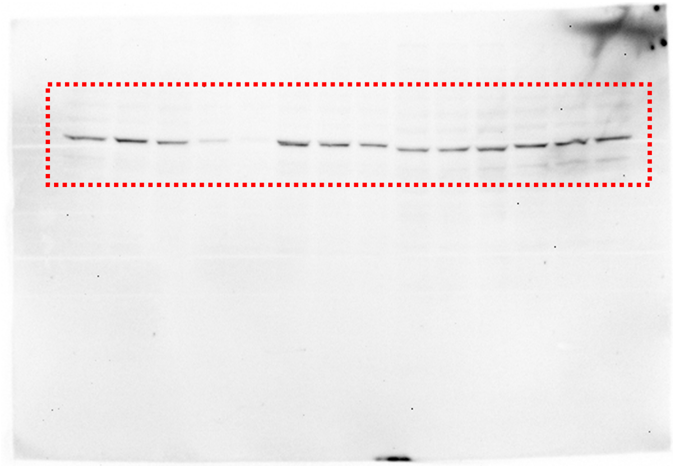

TCL: WB: TRIM21

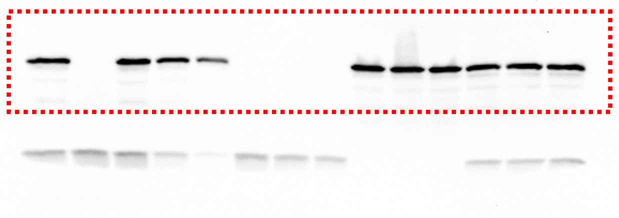

TCL: WB:ASC

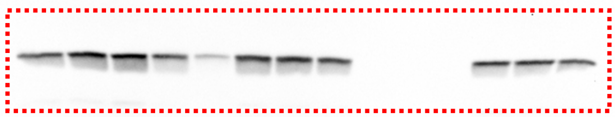

Figure 2c

TCL: WB: NLRP3

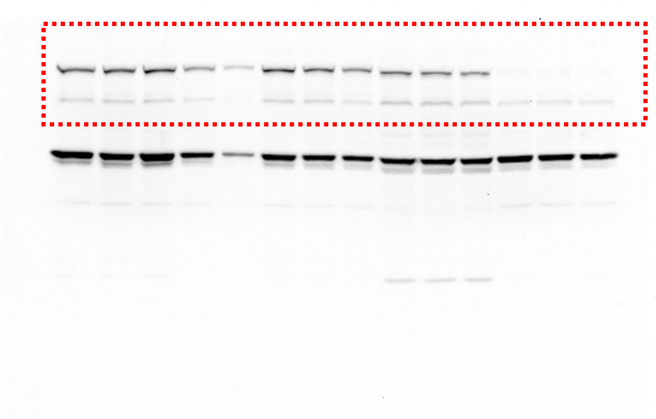

TCL: WB: β-tubulin

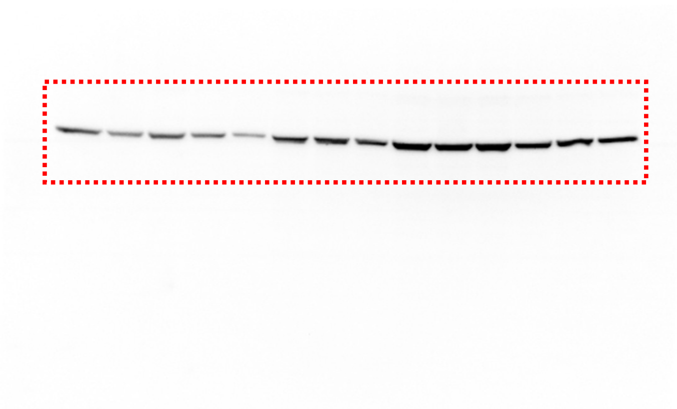

Figure 2f

IP: ASC, WB: NLRP3

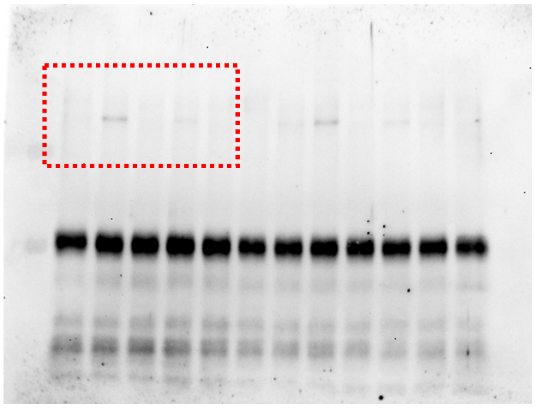

IP: ASC, WB: ASC

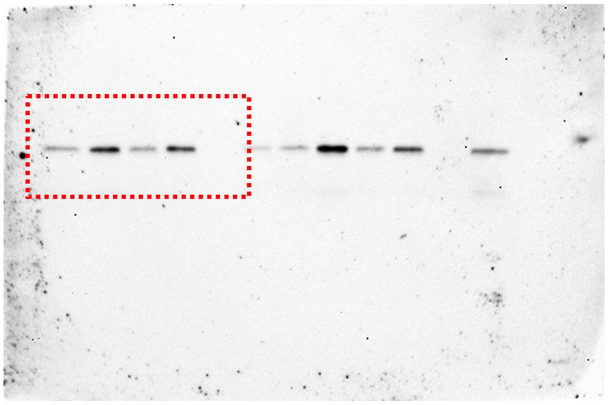

TCL: WB: NLRP3

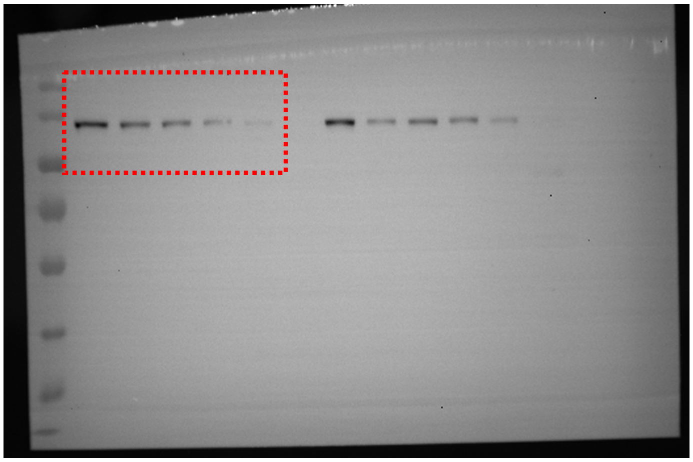

TCL: WB: ASC

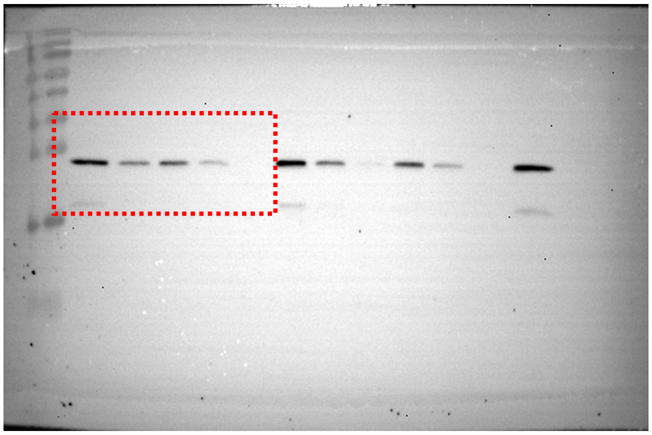

TCL: WB: β-tubulin

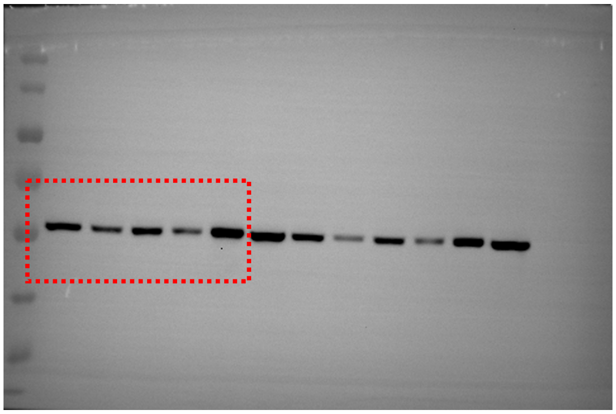

Figure 2g

WB: TRIM21

TCL: WB: GFP

TCL: WB: β-tubulin

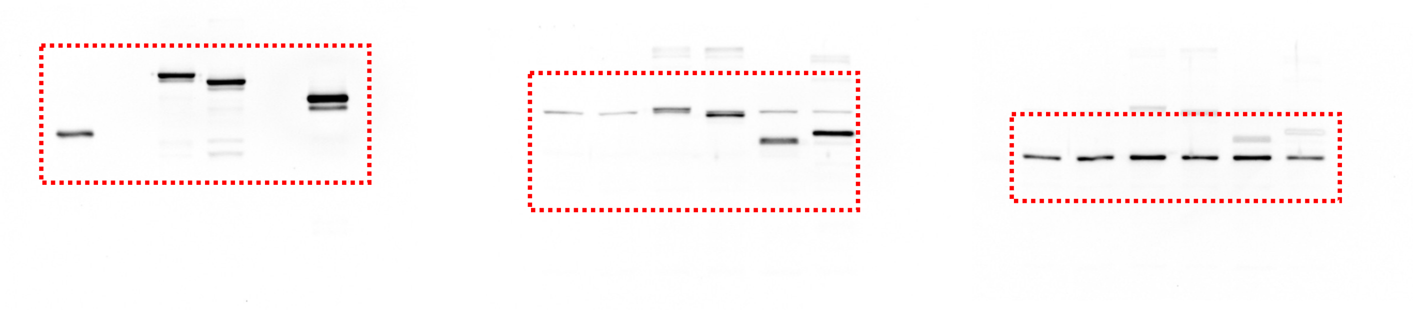

Figure 3b

SN: WB: cleaved Casp1

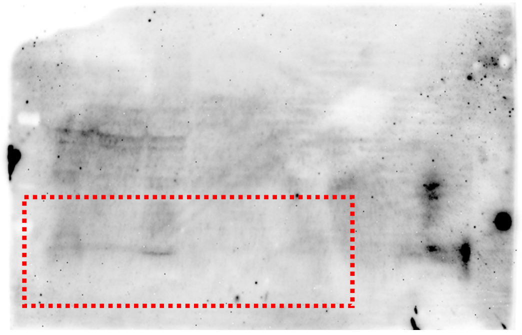

TCL: WB: Casp1

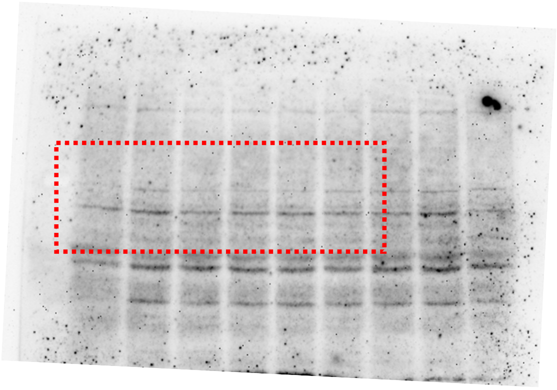

TCL: WB: GSDMD

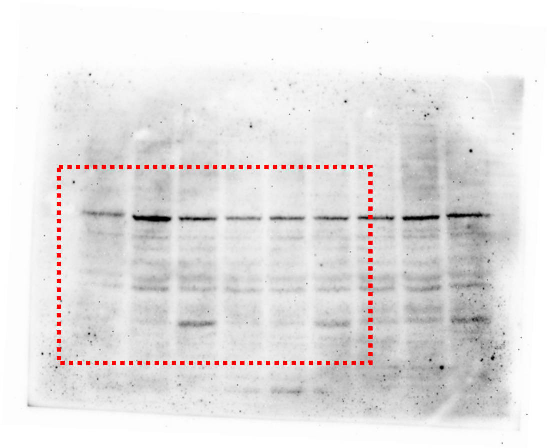

TCL: WB: Trim21

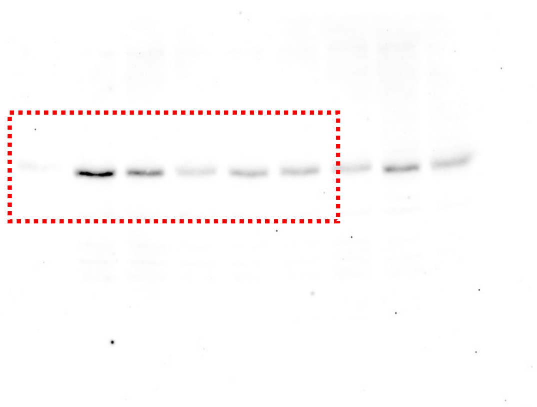

TCL: WB: β-tubulin

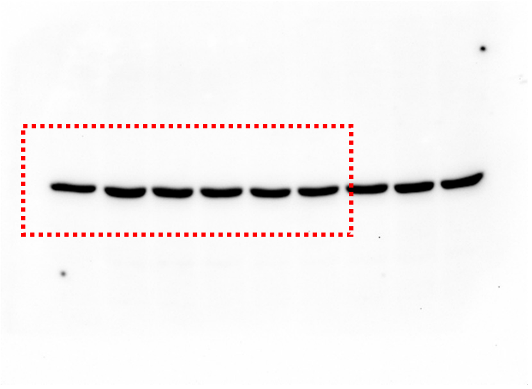

Figure 4b

TCL+DSS: WB: ASC

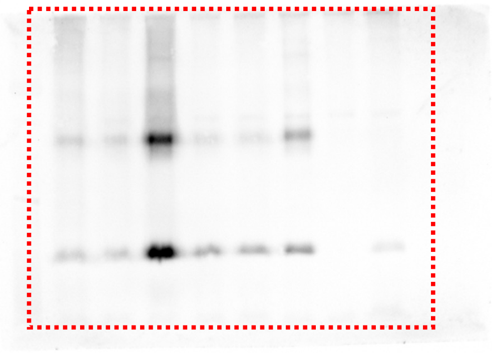

TCL: WB: ASC

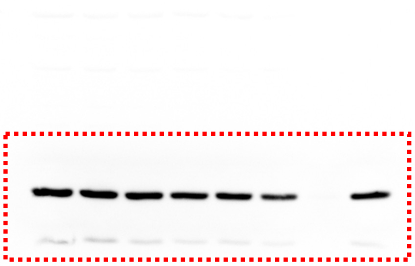

Figure 5h

released particles: WB: TRIM21

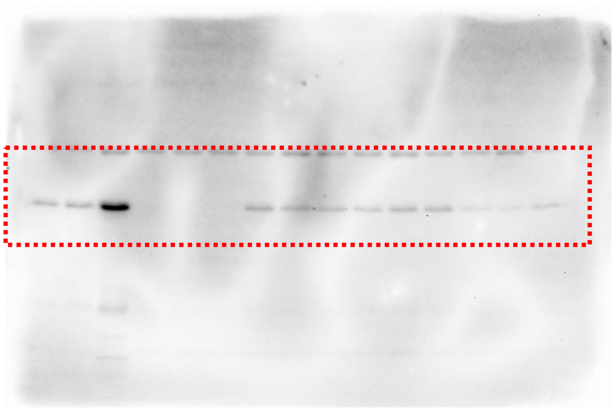

released particles: WB: NLRP3

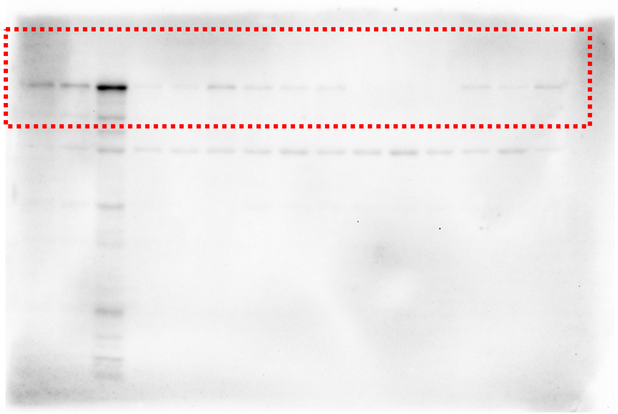

released particles: WB: ASC

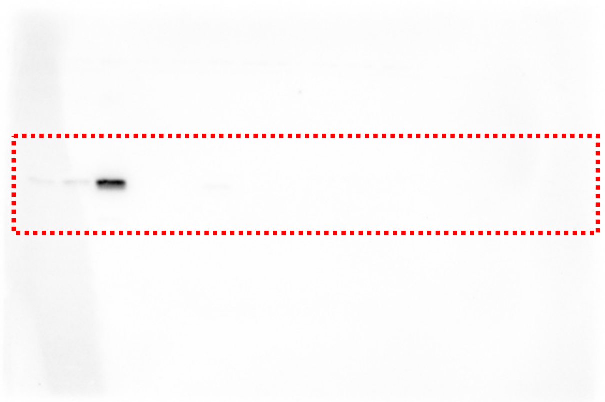

released particles: WB: cleaved Casp1

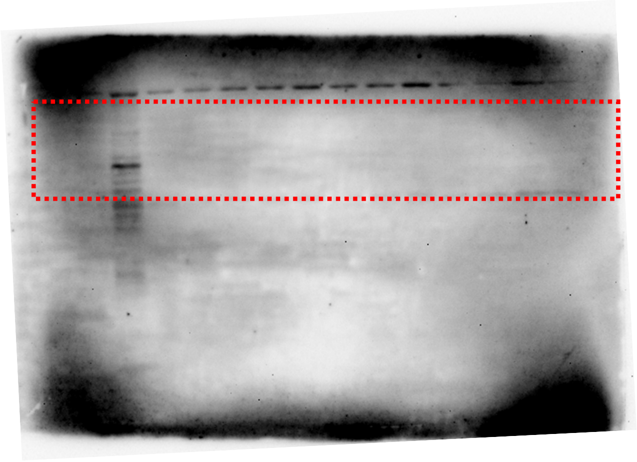

TCL: WB: TRIM21

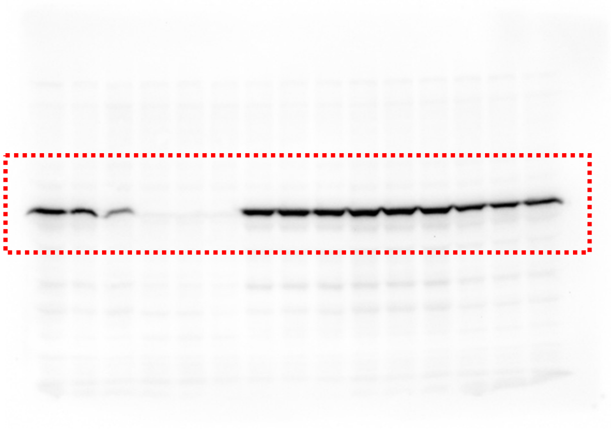

TCL: WB: ASC

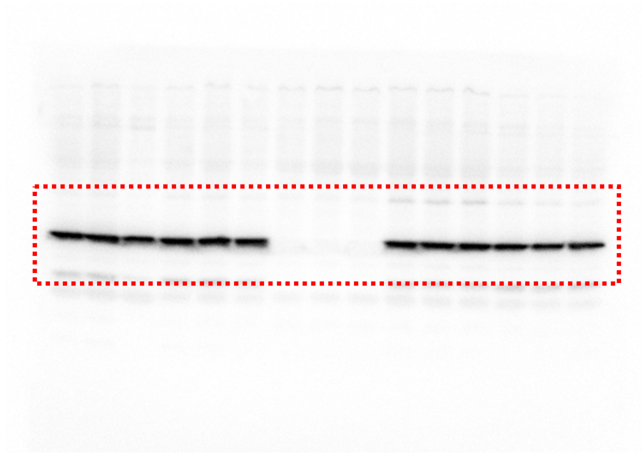

Figure 5h

TCL: WB: NLRP3

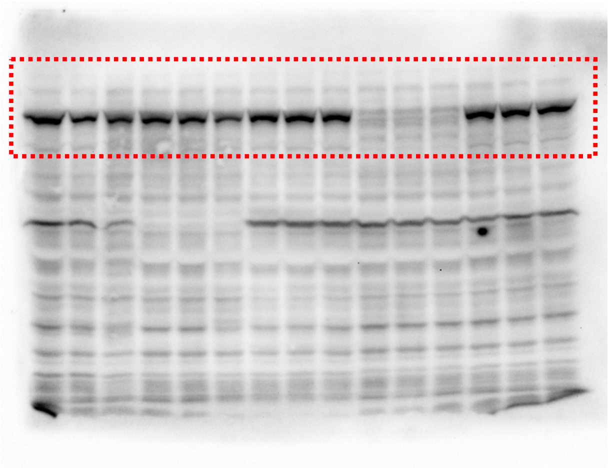

TCL: WB: Casp1

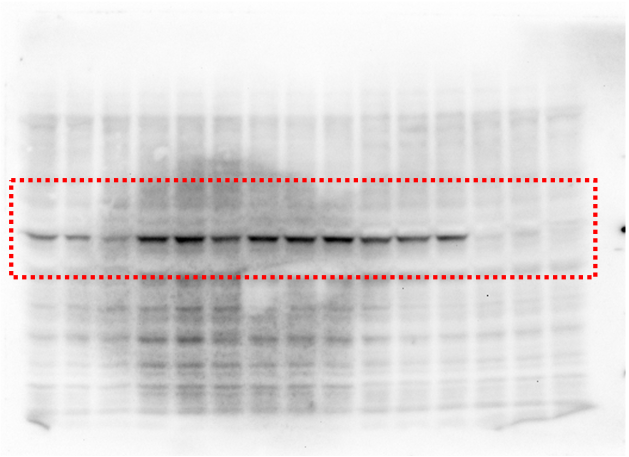

TCL: WB: β-tubulin

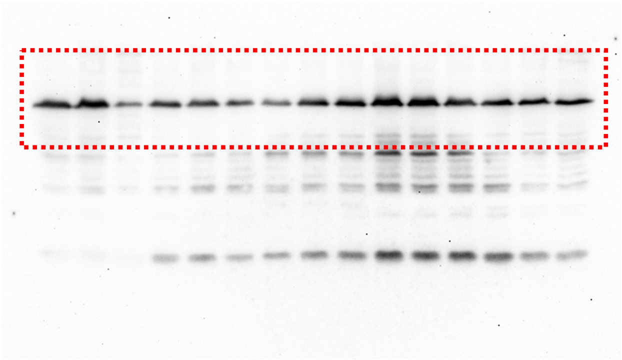

Figure 5i

SN IP: ASC: WB: TRIM21

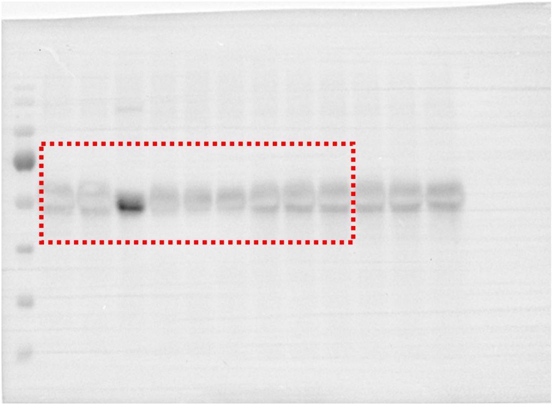

SN IP: ASC: WB: ASC

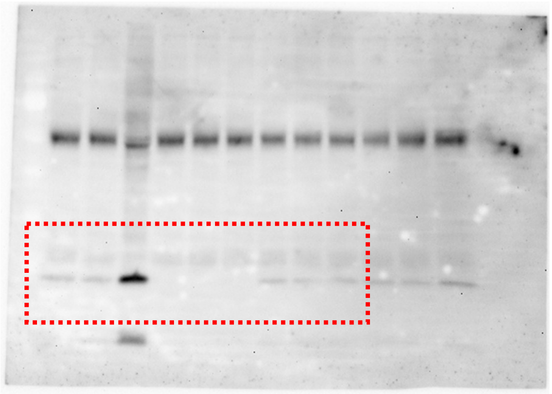

SN IP: ASC: WB: NLRP3

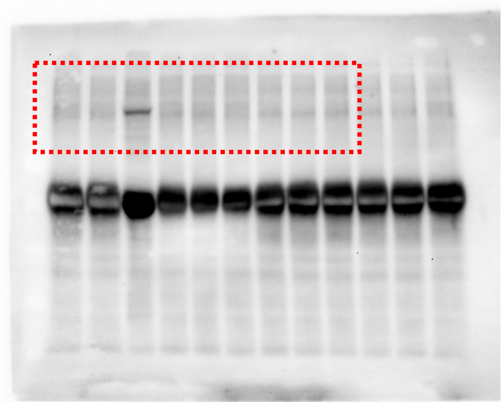

TCL: WB: TRIM21

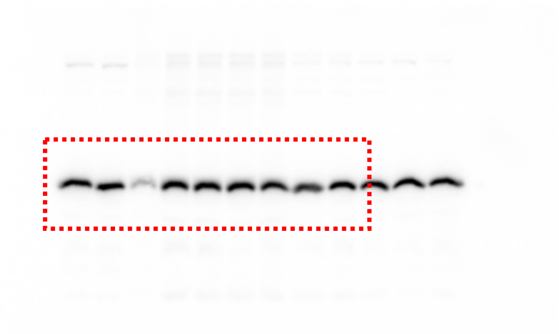

TCL: WB: ASC

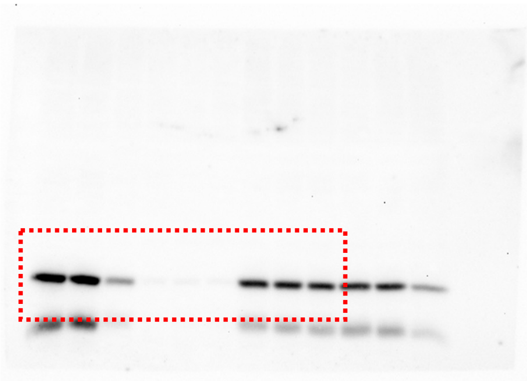

TCL: WB: NLRP3

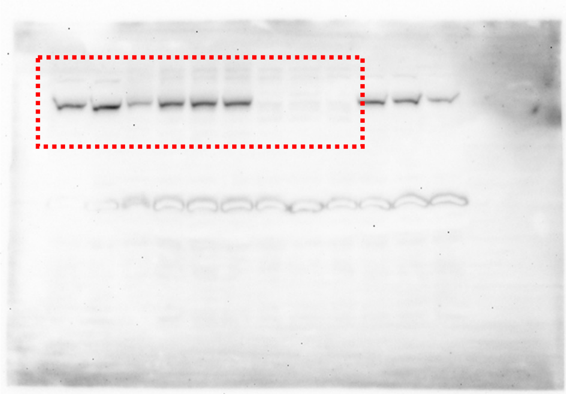

Extended data Figure 1b

WB: TRIM21

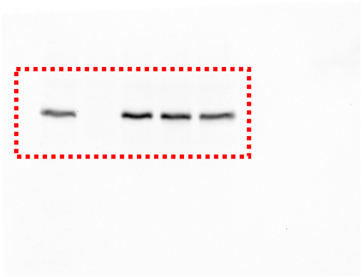

WB: ASC

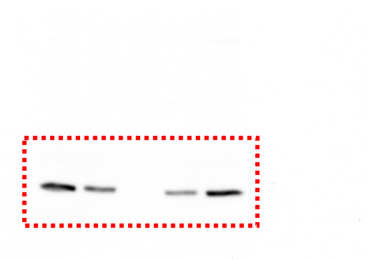

WB: NLRP3

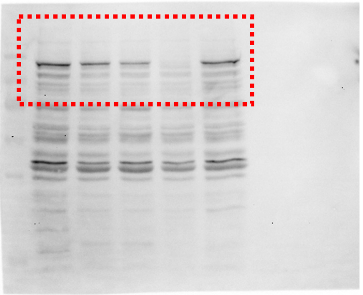

WB: CSP1

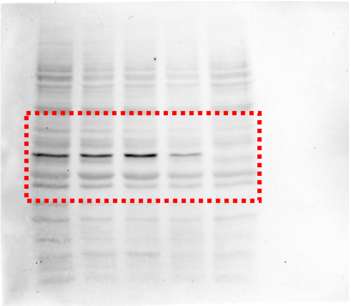

WB: β-Tubulin

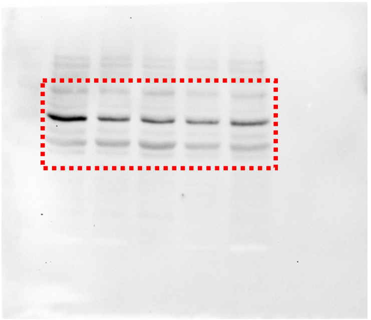

Extended data Figure 2d

WB: TRIM21

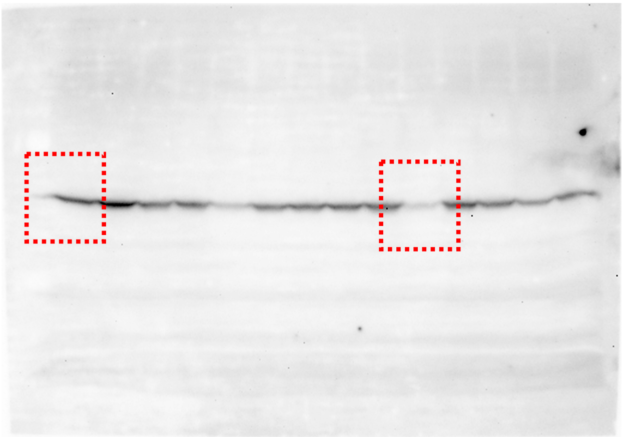

WB: β-Tubulin

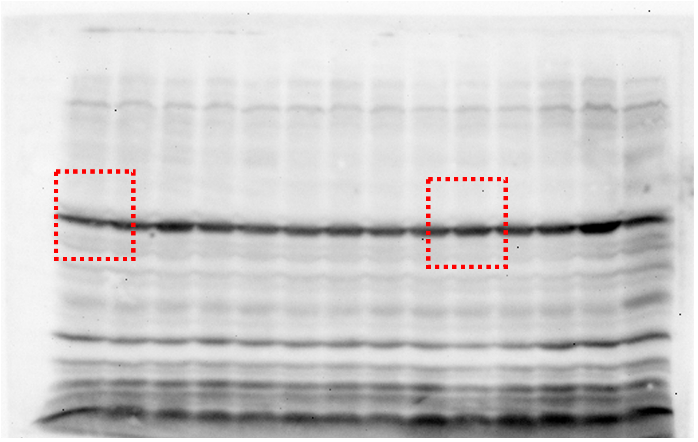

Extended data Figure 2f

SN: WB: Casp1

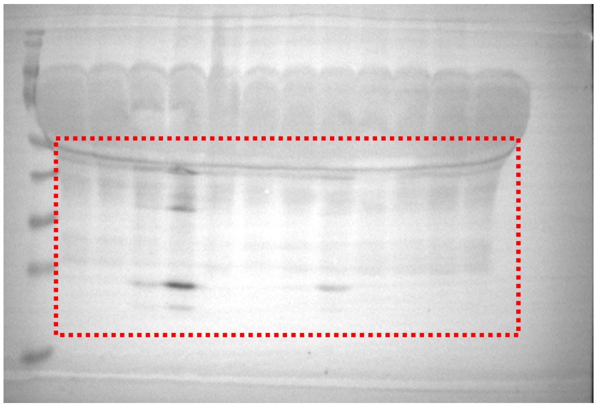

TCL: WB: Casp1

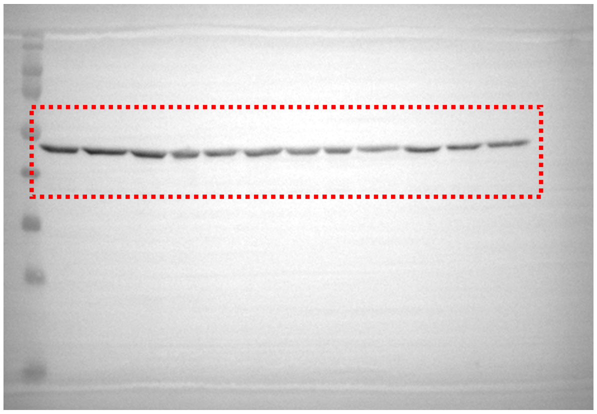

TCL: WB: GSDMD

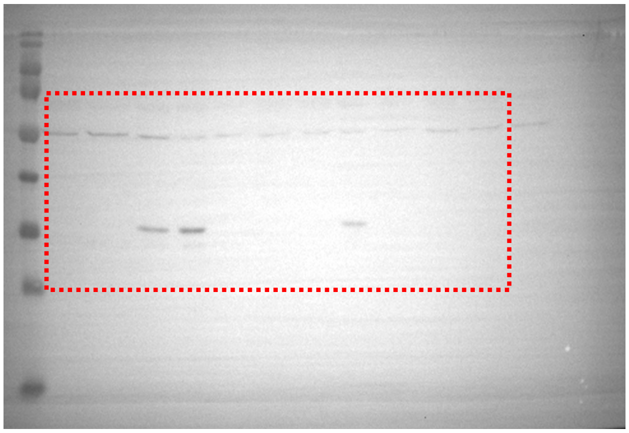

TCL: WB: TRIM21

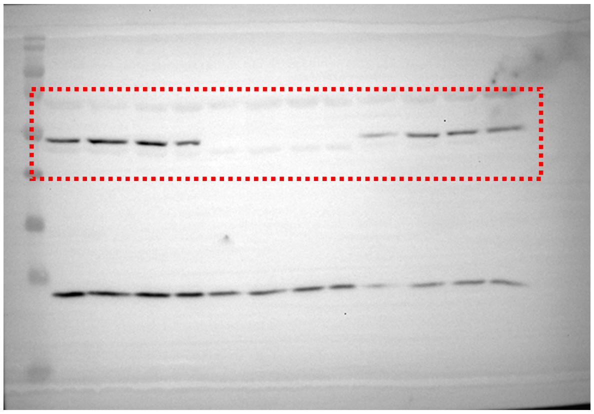

WB: β-Tubulin

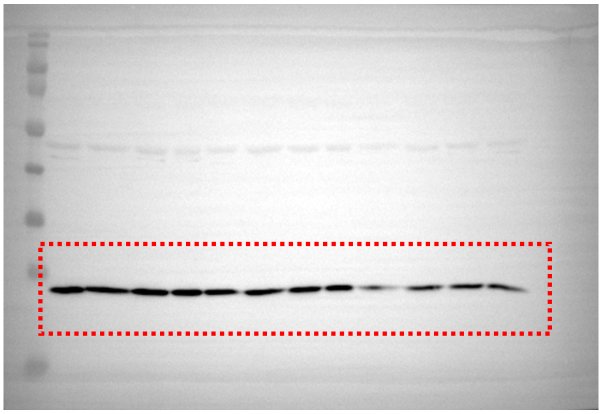

Reviewer Response Figure 1

AP: Ni^{2+}: WB: ASC

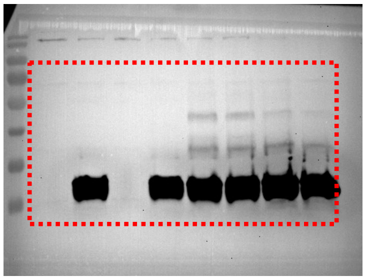

AP: Ni^{2+}: WB: Ubiquitin

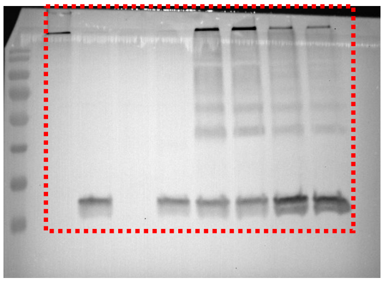

TCL: WB: ASC

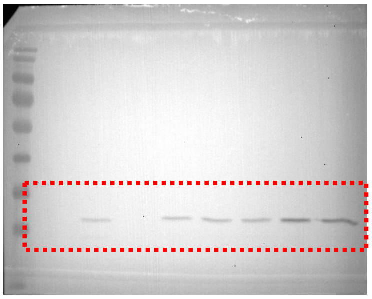

TCL: WB: TRIM21

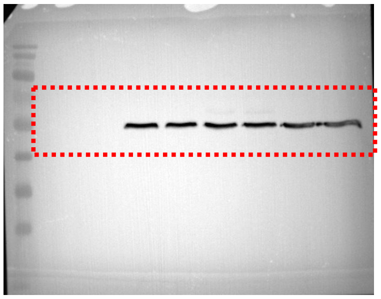

TCL: WB: Ubiquitin

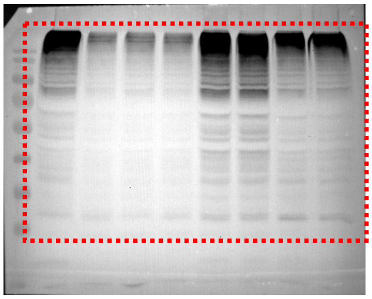

Supplement: Supplementary file 4 — Source Data [file 41467_2026_73350_MOESM4_ESM.zip › Source data revised/Carriere_original_WesternBlot_Images.pdf]
