# Supplementary material for: TRIM21 facilitates inflammasome assembly and contributes to autoinflammatory disease
Source: Nat Commun. 2026 May 22;17:6726. doi: 10.1038/s41467-026-73350-3 (PMC13385917; doi:10.1038/s41467-026-73350-3)
Supplement: Supplementary file 2 — Transparent Peer Review file [file 41467_2026_73350_MOESM2_ESM.pdf]

nature portfolio

# Peer Review File

# TRIM21 facilitates inflammasome assembly and contributes to autoinflammatory disease

Corresponding Author: Professor Christian Stehlik

**This file contains all reviewer reports in order by version, followed by all author rebuttals in order by version.**

Version 0:

Reviewer comments:

Reviewer #1

(Remarks to the Author)
The authors report that the E3 ubiquitin ligase, TRIM21, colocalizes with ASC during inflammasome assembly, mediates ASC ubiquitination, and is released from cells during pyroptosis. In human autoinflammatory diseases and in mice expressing autoinflammatory disease-associated mutant inflammasome components, particles containing TRIM21 and ASC can be detected in the serum. TRIM21-deficient cells have reduced inflammasome function. They conclude that TRIM21 is an essential component of inflammasomes. If correct, this is an important finding because TRIM21 is an autoantigen, and inflammasomes may contribute to its antigenicity. Furthermore, interference with TRIM21's roles in inflammasome assembly could represent a therapeutic option for autoinflammatory diseases.

Specific comments--major
1. Some assays (including immunostaining cells for polymerized ASC and TRIM21, described in lines 993-1007, and co-IP as described in lines 1200-1208) rely on co-immunoprecipitation or simultaneous binding of anti-ASC and anti-TRIM21 antibodies, or on detection of TRIM21 in material bound by anti-ASC antibodies. Because of the known high-affinity binding of TRIM21 to the immunoglobulin Fc domain, it is difficult to rule out the possibility that the Fc domains of the anti-ASC antibodies themselves contribute to the TRIM21-ASC associations observed. This caveat should be mentioned.

2. Figure 4, extended data fig. 5a: TRIM21 specks are not tightly associated with all ASC specks—instead, some of the apparent co-localization may be due to ASC specks superimposed on more diffuse TRIM21. Can the authors use confocal microscopy with Z-stacks to rigorously quantify co-localization, and can they discuss why specks form in the absence of TRIM21 if TRIM21 is truly a required?

3. Fig. 4h: All co-immunoprecipitation experiments with TRIM21 should be interpreted with caution since TRIM21 efficiently binds to immunoglobulin Fc domains. It is not unlikely that such binding is facilitated by clustering of Fc domains on aggregated ASC or NLRP3.

Other specific comments
4. Lines 202-203, 206-207, 212-217, 226-227, 233-234, and figures/figure legends throughout the manuscript: In most publications, more asterisks are used to designate greater statistical significance (lower p-values). Please use a consistent strategy to designate statistical significance throughout a given figure (e.g. *, $p < .05$; **, $p < 0.01$, ***, $p < .001$), as it is confusing to define statistical significance for each column of each graph as done here.

5. This also raises the issue of choosing appropriate statistical tests; when a single control condition is being compared to multiple test conditions, for example in figs. 1d and 1i, it is more appropriate to use one-way ANOVA followed by a post-test than to perform multiple T tests. In Fig. 4c, it would be more appropriate to use a repeated-measures ANOVA followed by a post-test rather than multiple T tests.

6. Lines 169-173: The statement that cleaved caspase-1 is "completely absent" in TRIM21-knockout cells is not supported by the blot shown; similarly, cleaved GSDMD-N is present in the TRIM21-deficient cell lysates.

7. Fig. 2a, c: For both panels, the figure legend says that BMDM were primed with Pam3CSK4, but the figures indicate they

were primed with LPS.

8. Fig. 3a, extended fig. 3b: ASC polymerization is reduced but not abolished in TRIM21-knockout cells. The conclusion that TRIM21 is 'required' for ASC polymerization may be an overstatement. This may be related to redundancy of E3 ubiquitin ligases, as the authors mention in lines 651-655.

9. Fig. 5e: The reported increase in TRIM21 mRNA in whole blood, and the decrease following Anakinra treatment, seem to be driven by a couple of outlying points and are likely not statistically significant. The authors may wish to revise the text in lines 533-535 to better reflect the data shown.

10. Fig. 5g: Presumably, the two points shown for each bar in this graph represent technical replicates from a single patient sample, rather than separate patients. The average of the two points should be used to calculate statistical significance.

Reviewer #2

(Remarks to the Author)
Thank you for the opportunity to read this excellent manuscript and congratulation on your essential findings. The study links for the first time TRIM21 to autoinflammatory disease. The detection of increased TRIM21 expression in whole blood from NOMID (CAPS) patients is striking. Moreover, the release of TRIM21⁺ASC⁺ particles and the detection of anti-TRIM21 (Ro52) autoantibodies in both mouse models and human patients with CAPS provide an intriguing link between inflammasome-driven inflammation and systemic autoimmunity. This could open new avenues for studying the role of the inflammasome in bridging autoimmune and autoinflammatory conditions. Manuscript is overall of high scientific value and novelty. The multimethodological approach employed in this study—integrating in vitro and in vivo systems, the use of complementary techniques to validate key findings - enhances the credibility of the data and enables plausible translation to clinical settings. Results are presented with meticulous attention and clarity.
A few points for the Authors for further consideration:
1. Anti-TRIM21 autoantibodies:
While the authors report TRIM21 release into the extracellular space and subsequent detection of anti-TRIM21 antibodies, it would strengthen the paper to clarify:
o Whether the autoantibodies recognize specific TRIM21 domains (e.g., coiled-coil vs PRY/SPRY), especially given that autoantibodies in SS and myopathies often target the coiled-coil region?
o Whether these antibodies correlate with disease severity or specific CAPS phenotypes?
2. Ubiquitination specificity and mechanism:
o Can the authors clarify the type of ubiquitination observed on ASC (e.g., K63 vs K48)? This would be helpful in interpreting whether ASC is marked for degradation or functional activation.
o Additionally, did the authors explored how exactly the ubiquitination process of ACS happens? Is it by direct action as E3 ligase or requires any indirect mechanisms?
3. TRIM21 expression downregulation by anakinra:
The observation that anakinra (IL-1 receptor antagonist) downregulates TRIM21 expression is intriguing and potentially paradoxical, since TRIM21 is shown to regulate IL-1β production. Do the authors propose a negative feedback loop? Could this reflect systemic cytokine normalization or changes in cell populations?
4. Co-localization of TRIM21 and ASC:
o Was co-localization of ASC and TRIM21 quantified using imaging methods (e.g., Pearson's coefficient)?
o Given that TRIM21 is known to shuttle between the cytoplasm and nucleus in response to stimuli, did the authors examine stimulus-specific localization dynamics?
o Were these experiments performed in primary monocytes or macrophages, or only in THP-1 cells?
5. Release mechanism of TRIM21⁺ASC⁺ particles:
o Can the Authors clarify how TRIM21⁺ASC⁺ particles are released - are they contained in exosomes or other types of extracellular vesicles, or are they a byproduct of pyroptosis?
o While possibly beyond the immediate scope of the study, it would be valuable check if systemic ASC+TRIM21+ particles are also detected in patients with autoimmune diseases
6. Cell death analysis:
As a marker of cell death, the authors use LDH release, which is rather non-specific. Did the Authors consider using more specific markers of pyroptosis?
7. Implications for autoimmunity and fibrosis:
While possibly beyond the immediate scope of the study, it would be valuable to explore whether patients with anti-TRIM21 autoantibodies exhibit increased inflammasome activation. This could help explain links to fibrotic disease, such as ILD, which may be perpetuated by low-grade inflammasome activation and cytokine production.
This work provides a major conceptual advance in our understanding of how TRIM21 contributes to inflammasome activation and propagation. It also raises fascinating questions about the interface between innate immunity and autoimmunity, and the role of released inflammasome particles as both danger signals and autoantigens. The identification of TRIM21 as a structural component of extracellular ASC particles and the detection of anti-TRIM21 autoantibodies in autoinflammatory syndromes establish a novel paradigm for understanding the origin of autoimmune responses. This study opens multiple avenues for future research, particularly in stratifying patients based on inflammasome biomarkers or autoantibody profiles.
Overall, we strongly support publication of this manuscript with minor clarifications as noted above.

Reviewer #3

(Remarks to the Author)
I co-reviewed this manuscript with one of the reviewers who provided the listed reports. This is part of the Nature Communications initiative to facilitate training in peer review and to provide appropriate recognition for Early Career Researchers who co-review manuscripts.

Reviewer #4

(Remarks to the Author)
Here the authors identify Ubiquitin ligase Trim21 as a novel regulator of the inflammasome by ubiquitinating ASC and co-localising with the ASC speck intra- and extra-celullarly. This work adds to the knowledge of how the ubiquitin systems controls inflammasome activation.
I enjoyed reading this paper. I think is very well planned and all the experiments follow a clear logic. Controls are also good for the experiments performed.
My comments about this work:
Results:
• In Fig 1B, how come you get a PLA signal in ASC-KO cells?
• In the experiments with different inflammasome activators, which clearly shows TRIM21 is involved in inflammasomes beyond NLRP3, authors should acknowledge that dA:dT activation in human macrophages is not dependent on AIM2, but STING-NLRP3. (DOI: 10.1016/j.cell.2017.09.039)
• In line 184 "…but also sufficient to promote NLRP3 inflammasome responses", what do you mean?
• Fig 1i, why is TNF not affected in any of the TRIM21 truncated versions? If TRIM21 regulates NfKB should it not?
• You need to indicate n numbers for WB and in general in the figures, as some of the flow data seems n=1 in ASC speck release experiments?
• I am confused, are TRIM21 viable? If so why do flox/Cre?
• I am aware that there are no selective inhibitors for TRIM21 and that makes things more complicated. However PYR41 data could be inhibiting any other step of inflammasome activation that requires ubiquitination, so need to be careful with the conclusions you make from its use.
• In Fig 5G, do you have any other markers to indicate severity of disease (I.e. IL-1 levels) or is this just based on described mutations?
• It is not clear to me from the results section if TRIM21 is actually acting on ASC-to ubiquitinate it and help assemble the complex or it ubiquitinates it in the complex. What do you propose? Do you hknow if TRIM21 is active when in the complex?
• As you will well know Ub-ligases work closely with deubiquitinases. USP7 has been shown to regulate NLRP3 inflammasome activation, and TRIM21-USP7 have been suggested to work together. Have you looked into this at all? Even if not, maybe this is something might be worth including in the discussion.
• 598-603 discussion, it feels like some references are missing here
• Define EMT

Version 1:

Reviewer comments:

Reviewer #1

(Remarks to the Author)
The authors have addressed most of my concerns in this revised manuscript. I still take issue with the statement that TRIM21 KO cells "TRIM21KO THP-1 cells and NLRP3KO controls largely lacked polymerized ASC specks" (line 376). As shown in fig. 4a, about 4-5% of TRIM21KO cells still formed specks compared to 28% of control cells, while NLRP3KO cells failed to form specks. It would be more accurate to say that TRIM21KO THP-1 cells had reduced ASC specks compared to control cells.

Reviewer #2

(Remarks to the Author)
We have read the revised manuscript with great interest. The Authors have made substantial efforts to enhance the quality of the work, resulting in significant improvement. We sincerely appreciate the additional experiments conducted to address our comments, as well as those raised by the other reviewers.
All of our previous concerns have been satisfactorily addressed. Just one final suggestion for the Authors' consideration - as both we and the other reviewers identified several points that extend beyond the scope of the current study, which the Authors have indicated they intend to pursue in future work, it may be beneficial to include a dedicated paragraph outlining prospective directions for future research.
Thank you.

Reviewer #3

(Remarks to the Author)
I co-reviewed this manuscript with one of the reviewers who provided the listed reports. This is part of the Nature Communications initiative to facilitate training in peer review and to provide appropriate recognition for Early Career

Researchers who co-review manuscripts.

Reviewer #4

(Remarks to the Author)
I am happy with how authors have addressed my comments.

**Open Access** This Peer Review File is licensed under a Creative Commons Attribution 4.0 International License, which permits use, sharing, adaptation, distribution and reproduction in any medium or format, as long as you give appropriate credit to the original author(s) and the source, provide a link to the Creative Commons license, and indicate if changes were made.
In cases where reviewers are anonymous, credit should be given to 'Anonymous Referee' and the source.
The images or other third party material in this Peer Review File are included in the article’s Creative Commons license, unless indicated otherwise in a credit line to the material. If material is not included in the article’s Creative Commons license and your intended use is not permitted by statutory regulation or exceeds the permitted use, you will need to obtain permission directly from the copyright holder.
To view a copy of this license, visit https://creativecommons.org/licenses/by/4.0/

**Reviewer Response (NCOMMS-25-42038-T-R1)**

We thank all reviewers for their thoughtful comments and are addressing all the suggested improvements below. Track changes was used to mark any changes in the revised manuscript.

**Reviewer #1**

The authors report that the E3 ubiquitin ligase, TRIM21, colocalizes with ASC during inflammasome assembly, mediates ASC ubiquitination, and is released from cells during pyroptosis. In human autoinflammatory diseases and in mice expressing autoinflammatory disease-associated mutant inflammasome components, particles containing TRIM21 and ASC can be detected in the serum. TRIM21-deficient cells have reduced inflammasome function. They conclude that TRIM21 is an essential component of inflammasomes. If correct, this is an important finding because TRIM21 is an autoantigen, and inflammasomes may contribute to its antigenicity. Furthermore, interference with TRIM21's roles in inflammasome assembly could represent a therapeutic option for autoinflammatory diseases.

Major:

*1. Some assays (including immunostaining cells for polymerized ASC and TRIM21, described in lines 993-1007, and co-IP as described in lines 1200-1208) rely on co-immunoprecipitation or simultaneous binding of anti-ASC and anti-TRIM21 antibodies, or on detection of TRIM21 in material bound by anti-ASC antibodies. Because of the known high-affinity binding of TRIM21 to the immunoglobulin Fc domain, it is difficult to rule out the possibility that the Fc domains of the anti-ASC antibodies themselves contribute to the TRIM21-ASC associations observed. This caveat should be mentioned.*

We included this caveat in the discussion (lines 664-666). However, we would like to emphasize that we utilized cells expressing TRIM21 and ASC fused to fluorescent proteins (EGFP-TRIM21 and mScarlet-ASC) for co-localization experiments by microscopy (Fig. 5b, c, d and ED Fig. 5, 6) and for released TRIM21-ASC particle analysis by flow cytometry (Fig. 5j, ED Fig. 7) to avoid any possible non-specific IgG binding. Further, we now also performed $Ni^{2+}$ affinity purification of His-tagged TRIM21 (Fig. 1c), which also demonstrates binding of TRIM21 to ASC independently of antibodies.

*2. Figure 4, extended data fig. 5a: TRIM21 specks are not tightly associated with all ASC specks—instead, some of the apparent co-localization may be due to ASC specks superimposed on more diffuse TRIM21. Can the authors use confocal microscopy with Z-stacks to rigorously quantify co-localization, and can they discuss why specks form in the absence of TRIM21 if TRIM21 is truly a required?*

We calculated the Manders' and Pearson's co-localization coefficients from deconvolved z-stacks, showing significantly increased ASC-TRIM21 co-localization within the speck compared to the remainder of the cell, and included the results as Fig 5d. Representative z-series and volumetric 3D images are shown in Extended Data Fig. 6, demonstrating co-localization of ASC and TRIM21. By flow cytometry and cross-linking we detect a minor background of polymerized ASC in *TRIM21* KO cells. We believe this is background due to the sensitive nature of the approach. The cells used for the co-localization experiments with tagged TRIM21 also express endogenous TRIM21, which is likely responsible for the ASC polymerization in cells not expressing EGFP-TRIM21. However, we cannot completely exclude that a minor subset of ASC polymers form in the absence of TRIM21 due to other mechanisms that allow to overcome this defect. We added a sentence to the discussion (line 696-698).

*3. Fig. 4h: All co-immunoprecipitation experiments with TRIM21 should be interpreted with caution since TRIM21 efficiently binds to immunoglobulin Fc domains. It is not unlikely that such binding is facilitated by clustering of Fc domains on aggregated ASC or NLRP3.*
Please see our response to question #1.

Minor:

*4. Lines 202-203, 206-207, 212-217, 226-227, 233-234, and figures/figure legends throughout the manuscript: In most publications, more asterisks are used to designate greater statistical significance (lower p-values). Please use a consistent strategy to designate statistical significance throughout a given figure (e.g. *, p < .05; **, p < 0.01, ***, p < .001), as it is confusing to define statistical significance for each column of each graph as done here.*

To indicate statistical significance, we are now using *P ≤ 0.05, **P ≤ 0.01, ***P ≤ 0.001, and ****P ≤ 0.0001, as suggested.

*5. This also raises the issue of choosing appropriate statistical tests; when a single control condition is being compared to multiple test conditions, for example in figs. 1d and 1i, it is more appropriate to use one-way ANOVA followed by a post-test than to perform multiple T tests. In Fig. 4c, it would be more appropriate to use a repeated-measures ANOVA followed by a post-test rather than multiple T tests.*

We recalculated statistics for all multi-group data using ANOVA with post test and indicate the used test in the figure legends.

*6. Lines 169-173: The statement that cleaved caspase-1 is "completely absent" in TRIM21-knockout cells is not supported by the blot shown; similarly, cleaved GSDMD-N is present in the TRIM21-deficient cell lysates.*

We changed these statements to "almost completely absent" (line 218) and "significantly diminished" (line 221).

*7. Fig. 2a, c: For both panels, the figure legend says that BMDM were primed with Pam3CSK4, but the figures indicate they were primed with LPS.*

We corrected this error in the figure legend (now Fig. 3a, c).

*8. Fig. 3a, extended fig. 3b: ASC polymerization is reduced but not abolished in TRIM21-knockout cells. The conclusion that TRIM21 is 'required' for ASC polymerization may be an overstatement. This may be related to redundancy of E3 ubiquitin ligases, as the authors mention in lines 651-655.*

We changed the wording to "largely lack ASC polymerization" (line 376) and "TRIM21 had an important role in ASC polymerization" (line 380).

*9. Fig. 5e: The reported increase in TRIM21 mRNA in whole blood, and the decrease following Anakinra treatment, seem to be driven by a couple of outlying points and are likely not statistically significant. The authors may wish to revise the text in lines 533-535 to better reflect the data shown.*

We agree and therefore we did not indicate that this is a significant result. We included it to show a trend, but due to the limited subject number statistical significance was not reached. We added that this is only a trend in the revised text (line 593.

*10. Fig. 5g: Presumably, the two points shown for each bar in this graph represent technical replicates from a single patient sample, rather than separate patients. The average of the two points should be used to calculate statistical significance.*

We removed the individual data points and used the average of duplicate measurements to calculate statistical significance.

**Reviewer #2**

Thank you for the opportunity to read this excellent manuscript and congratulation on your essential findings. The study links for the first time TRIM21 to autoinflammatory disease. The detection of increased TRIM21 expression in whole blood from NOMID (CAPS) patients is striking. Moreover, the release of $TRIM21^{+}ASC^{+}$ particles and the detection of anti-TRIM21 (Ro52) autoantibodies in both mouse models and human patients with CAPS provide an intriguing link between inflammasome-driven inflammation and systemic autoimmunity. This could open new avenues for studying the role of the inflammasome in bridging autoimmune and autoinflammatory conditions. Manuscript is overall of high scientific value and novelty. The multimethodological approach employed in this study—integrating in vitro and in vivo systems, the use of complementary techniques to validate key findings - enhances the credibility of the data and enables plausible translation to clinical settings. Results are presented with meticulous attention and clarity.

*1. Anti-TRIM21 autoantibodies: While the authors report TRIM21 release into the extracellular space and subsequent detection of anti-TRIM21 antibodies, it would strengthen the paper to clarify:*
*a. Whether the autoantibodies recognize specific TRIM21 domains (e.g., coiled-coil vs PRY/SPRY), especially given that autoantibodies in SS and myopathies often target the coiled-coil region?*

As the reviewer indicated, a majority of autoantibodies in systemic autoimmune rheumatic diseases target the coiled coil region, but auto-antibodies can also target the RING domain (DOI 10.1186/s13075-015-0871-3). To address this question, we purified mouse full length TRIM21 and TRIM21 domain deletions from transiently transfected HEK293 cells as well as human TRIM21 and TRIM21 domain deletions from stable expressing THP-1 cells and then used mouse and human CAPS sera or plasma as source for autoantibodies. Due to the very limited human samples, we only tested the patients that showed antibody responses in our earlier experiments. In contrast to systemic autoimmune rheumatic diseases, we observed that the anti-TRIM21 autoantibodies in CAPS mice and our human patients target all TRIM21 domains. We added these data as Fig 6j and k.

*b. Whether these antibodies correlate with disease severity or specific CAPS phenotypes?*

We did not observe any correlation between disease severity and anti-TRIM21 antibody titer in our small patient cohort.

*2. Ubiquitination specificity and mechanism:*
*a. Can the authors clarify the type of ubiquitination observed on ASC (e.g., K63 vs K48)? This would be helpful in interpreting whether ASC is marked for degradation or functional activation.*

Using ubiquitin expression constructs that have all (Ub KO) or all but one of the 7 lysine residues mutated to arginine Ub KO-6K, Ub KO-11K, Ub KO-27K, Ub KO-29K, Ub KO-33K, Ub KO-48K, Ub KO-63K we observed that the common canonical ubiquitin linkages K48 and K63 are not utilized, but rather the non-canonical K27 and K29. We added the results of this experiment as Fig. 1f and 1g.

*b. Additionally, did the authors explored how exactly the ubiquitination process of ACS happens? Is it by direct action as E3 ligase or requires any indirect mechanisms?*

Although we did not directly investigate ASC ubiquitination due to the established role of TRIM21 as an E3 ubiquitin ligase, our functional results indicate that the RING domain of TRIM21, which contains the catalytic domain, is required for inflammasome activation (Fig. 2g, h). We added this information to the discussion (line 699).

*3. TRIM21 expression downregulation by anakinra: The observation that anakinra (IL-1 receptor antagonist) downregulates TRIM21 expression is intriguing and potentially paradoxical, since TRIM21 is shown to regulate IL-1β production. Do the authors propose a negative feedback loop? Could this reflect systemic cytokine normalization or changes in cell populations?*

This is an intriguing concept to be investigated in follow up studies. Indeed, there is evidence of IL-1R-mediated regulation of type I and type III interferon responses (doi.org/10.1128/mbio.00342-18, doi.org/10.1086/339410, doi.org/10.1101/2024.01.08.574713), which could affect type I/III interferon-induced expression of TRIM21. We did describe earlier that other regulators of the inflammasome, the PYRIN domain only proteins (POPs), are also functioning in an IL-1β-regulated inflammasome feedback loop.

*4. Co-localization of TRIM21 and ASC:*
*a. Was co-localization of ASC and TRIM21 quantified using imaging methods (e.g., Pearson's coefficient)?*

Please see our response to Reviewer 1 #2.

*b. Given that TRIM21 is known to shuttle between the cytoplasm and nucleus in response to stimuli, did the authors examine stimulus-specific localization dynamics?*

We also observed some nuclear TRIM21 in our staining, but in the current study we focused on inflammasome assembly in the cytosol and release of polymerized ASC/TRIM21 particles. We are planning to investigate stimulus-specific localization dynamics in our follow up studies.

*5. Were these experiments performed in primary monocytes or macrophages, or only in THP-1 cells?*

We silenced TRIM21 in primary human peripheral blood-derived macrophages and determined IL-1β release (ED Fig. 1c) and utilized primary mouse BMDM in Fig. 3a, b, c, ED Fig. 2b, c, f, as well as immortalized iBMDM (ED Fig. 2d, e).

*6. Release mechanism of TRIM21$^+$ASC$^+$ particles:*
*a. Can the Authors clarify how TRIM21$^+$ASC$^+$ particles are released - are they contained in exosomes or other types of extracellular vesicles, or are they a byproduct of pyroptosis?*

While ASC containing pyroptotic extracellular vesicles with anti-inflammatory activity through blocking of TLR4 have been described (doi:10.1016/j.ymthe.2023.12.008), it is most commonly believed that inflammasome particles are membrane free (doi:10.1038/ni.2913, doi:10.1038/ni.2919). We utilized the GSDMD pore formation inhibitor disulfiram (DSF), which significantly reduced ASC and TRIM21 release after inflammasome activation (Fig. 4d and 5f), which indicates that the GSDMD-dependent ASC release is mediated by pyroptosis. We indicated this in the text (lines 390 and 464).

*b. While possibly beyond the immediate scope of the study, it would be valuable check if systemic ASC+TRIM21+ particles are also detected in patients with autoimmune diseases*

This is indeed a very interesting question that we are planning to pursue in future studies but in this manuscript we specifically wanted to focus on the newly discovered TRIM21 autoantibodies and particle release in autoinflammation.

*7. Cell death analysis: As a marker of cell death, the authors use LDH release, which is rather non-specific. Did the Authors consider using more specific markers of pyroptosis?*

While LDH alone is a general marker of cell death, we are using the same cell types and conditions to also show unique characteristics of pyroptosis. For human cell death (Fig. 2e) we are also showing pyroptosis specific proteolytic processing of caspase-1 and gasdermin D (Fig. 2c) and caspase-1 activity by FLICA (Fig. 2d) as well as ASC polymerization and inflammasome component containing particle release (ED Fig.3b, c, Fig. 4a-e, Fig. 5h-j). For mouse BMDM death (Fig. 3c) we are also showing pyroptosis specific proteolytic processing of caspase-1 and gasdermin D (Fig. 3b). Collectively these assays are considered to be hallmarks of pyroptosis.

*8. Implications for autoimmunity and fibrosis: While possibly beyond the immediate scope of the study, it would be valuable to explore whether patients with anti-TRIM21 autoantibodies exhibit increased inflammasome activation. This could help explain links to fibrotic disease, such as ILD, which may be perpetuated by low-grade inflammasome activation and cytokine production.*

While extending this study to autoimmunity is one of our future goals we found that it is beyond the scope of this manuscript, which is specifically focused on novel aspects in autoinflammation.

*This work provides a major conceptual advance in our understanding of how TRIM21 contributes to inflammasome activation and propagation. It also raises fascinating questions about the interface between innate immunity and autoimmunity, and the role of released inflammasome particles as both danger signals and autoantigens. The identification of TRIM21 as a structural component of extracellular ASC particles and the detection of anti-TRIM21 autoantibodies in autoinflammatory syndromes establish a novel paradigm for understanding the origin of autoimmune responses. This study opens multiple avenues for future research, particularly in stratifying patients based on inflammasome biomarkers or autoantibody profiles. Overall, we strongly support publication of this manuscript with minor clarifications as noted above.*

We thank the reviewer for the encouraging assessment of our manuscript and excitement for our planned future studies.

**Reviewer #3 and Reviewer #4 (co-review)**

Here the authors identify Ubiquitin ligase Trim21 as a novel regulator of the inflammasome by ubiquitinating ASC and co-localising with the ASC speck intra- and extra-celullarly. This work adds to the knowledge of how the ubiquitin systems controls inflammasome activation. I enjoyed reading this paper. I think is very well planned and all the experiments follow a clear logic. Controls are also good for the experiments performed. My comments about this work:

*1. In Fig 1B, how come you get a PLA signal in ASC-KO cells?*

We believe that this is background due to the very sensitive nature of the PLA approach which is based on signal amplification. This result however, clearly demonstrates the loss of co-localization (PLA signal) in *TRIM21* KO and *ASC* KO cells compared to wild type cells.

*2. In the experiments with different inflammasome activators, which clearly shows TRIM21 is involved in inflammasomes beyond NLRP3, authors should acknowledge that dA:dT activation in human macrophages is not dependent on AIM2, but STING-NLRP3. (DOI: 10.1016/j.cell.2017.09.039).*

Contrary to human monocytes and BlaER cells, THP-1 cells have AIM2 and not NLRP3 dependent inflammasome activation in response to cytosolic DNA, which was also observed and acknowledged as

cell type specific responses by Dr Hornung's group in Fig. S1D of the referenced study. Hence, our results agree with the findings of Dr Hornung's group, which we added to our manuscript. (line 211-213)

*3. In line 184 "…but also sufficient to promote NLRP3 inflammasome responses", what do you mean?*

We removed this sentence but initially intended to highlight that adding TRIM21 to otherwise identical conditions it alone is able to enhance the response in our inflammasome reconstitution assay in HEK293 cells.

*4. Fig 1i, why is TNF not affected in any of the TRIM21 truncated versions? If TRIM21 regulates NfKB should it not?*

There are conflicting observations about NF-κB dependent TRIM21 regulation, which we also mention in the discussion starting at line 754. We observed only very minor or no significant differences regarding NF-κB in our experiments and our cell systems.

*5. You need to indicate n numbers for WB and in general in the figures, as some of the flow data seems n=1 in ASC speck release experiments?*

We added this information and the indicated experiments (WB, flow cytometry) show representative results.

*6. I am confused, are TRIM21 viable? If so why do flox/Cre?*

Conventional KO mice are viable, but the described lines showed inconsistent and conflicting phenotypes and effects on NF-κB and type I IFN signaling and at least one strain showed functional compensation by upregulating other TRIM family proteins. This, together with the ability to generate myeloid specific *Trim21* deletion for studying functional consequences of myeloid inflammasome activation, was the rationale for generating conditional KO mice. Surprisingly, mice with homozygous TRIM21 deletion in myeloid cells are not viable, possibly because there is no comparable compensation by other TRIM family members as in the conventional *Trim21* KO mice (doi/10.4049/jimmunol.0804121), but we currently do not know the mechanism leading to embryonic lethality. Therefore, we performed experiments with heterozygous mice showing significantly reduced TRIM21 RNA (ED Fig.2b) and protein (Fig.3b) expression.

*7. I am aware that there are no selective inhibitors for TRIM21 and that makes things more complicated. However PYR41 data could be inhibiting any other step of inflammasome activation that requires ubiquitination, so need to be careful with the conclusions you make from its use.*

We did not intend to imply TRIM21 specific inhibition with PYR41 and avoided this conclusion by mentioning its E1 ubiquitin ligase inhibitor function in the text (line 386). We only included PYR41 and DSF to show that ubiquitination and GSDMD-mediated pyroptosis, respectively, are required for ASC and TRIM21 particle release.

*8. In Fig 5G, do you have any other markers to indicate severity of disease (I.e. IL-1 levels) or is this just based on described mutations?*

Classification is based on the described mutations and their well described associated disease severity. Unfortunately, we could not detect IL-1β in our patient cohort, even with a high-sensitivity ELISA.

*9. It is not clear to me from the results section if TRIM21 is actually acting on ASC-to ubiquitinate it and help assemble the complex or it ubiquitinates it in the complex. What do you propose? Do you know if TRIM21 is active when in the complex?*

TRIM21 and ASC interaction (Fig 1c) and TRIM21-mediated ASC ubiquitination (Fig 1d) in 293 cells, which do not express any other inflammasome components, indicates that interaction and ubiquitination occur independent of complex formation but we cannot rule out a role for continued TRIM21 activity within the complex. We show ASC and TRIM21 interaction/co-localization in naïve cells (Fig. 1b, Fig. 5b, c) and that ASC polymerization is prevented in the absence of NLRP3 and TRIM21 (Fig. 4b). Newly added results also show that ASC is not recruited to NLRP3 in *TRIM21*$^{KO}$ cells (Fig. 2f). We therefore propose that TRIM21 interacts and ubiquitinates ASC to enable ASC recruitment to NLRP3 (or other PRRs) for subsequent ASC polymerization, inflammasome assembly and activation. We added this to the discussion (line 747-750).

*10. As you will well know Ub-ligases work closely with deubiquitinases. USP7 has been shown to regulate NLRP3 inflammasome activation, and TRIM21-USP7 have been suggested to work together. Have you looked into this at all? Even if not, maybe this is something might be worth including in the discussion.*

The reviewer is bringing up a very intriguing suggestion and we included this possibility in the discussion (line 680-684) and therefore we performed experiments to address this question. We co-expressed His-ASC with TRIM21 and ubiquitin in HEK293 cells and affinity purified His-ASC from cell lysates using magnetic $Ni^{2+}$ NTA beads. After affinity purification of ASC, we incubated the $Ni^{2+}$ NTA beads with 50 nM recombinant USP7 in assay buffer for 1h at 37°C and analyzed ASC ubiquitination by Western blot. Incubation with USP7 did not affect ubiquitination of ASC in 2 experiments (Reviewer Fig.1). Also, newly added results indicate that TRIM21 mediates K27- and K29-linked ubiquitination of ASC. Since there are reports that K27 ubiquitination provides resistance to most deubiquitinases (10.1016/j.str.2016.01.007) we hypothesize that TRIM21 mediated K27- and K29-linked ubiquitination of ASC is resistant to deubiquitination by USP7. We did not include these very preliminary results in the manuscript but are planning to investigate known K27 and K29 deubiquitinases in our future studies.

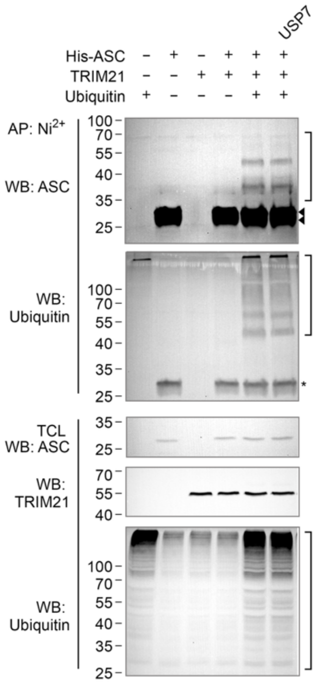

**Reviewer Fig 1.** HEK293 cells were transiently transfected with His-ASC, TRIM21 and ubiquitin. ASC was purified with $Ni^{2+}$-NTA beads and immunocomplexes incubated with or without recombinant USP7 (50 nM) in assay buffer for 1h at 37°C, as indicated and separated by SDS/PAGE immunoblot.

*11. 598-603 discussion, it feels like some references are missing here.*

We added the review article to remain within the citation limits but added now the primary references for this revision.

*12. Define EMT*

Epithelial-mesenchymal transition. We removed the abbreviation in the manuscript.

## Editor

*We consider it particularly important that you address the concerns raised by Refs #1, 2, 3 regarding colocalization experiments. In addition, verify the type of ubiquitination observed on ASC and provide additional mechanistic insights as indicated by Refs #2, 3, and 4. Meanwhile, provide additional markers of pyroptosis (Refs #2 and #3) and repeat the main experiments using primary monocyte or macrophages (Refs #2 and #3). We consider it important that you further investigate the relevance of the findings for autoimmunity and fibrosis (Refs #2 and 3).*

- We addressed these concerns and described it in the above response to the reviewers:
- colocalization experiments: R1 #1, #2, R2 #4;
- type of ubiquitination observed on ASC: R2 #2;
- provide additional markers of pyroptosis: R2 #7;
- experiments using primary monocyte or macrophages: R2 #5;

*We consider it important that you further investigate the relevance of the findings for autoimmunity and fibrosis:* *R2 #8*

The reviewer (R2 #8) acknowledged that this is possibly beyond the scope of this study and unfortunately we currently do not have access to a fibrosis patient cohort. For this manuscript our focus was specifically on autoinflammation but we hope to follow up on this question in our future studies.

Finally, to ensure the validity and reproducibility of the results and conclusions, and in observation of the complex and variable nature of most immunological studies, we ask that all experiments should be performed in at least two to three independent batches, or have at a minimum 5-10 independent biological replicates, barring clear exceptions such as case reports or non-human primate studies. Please note that technical replicates (e.g. triplicate wells in qPCR or tissue culture, or duplicate western blots run with the same lysates) count only as one data point. Lastly, the number of data points and independent experiments should be stated clearly in all figure legends. Concomitantly, please carefully revise the statistical analysis throughout the manuscript.

We revised the statistical analysis and indicated the “n” and statistical methods in the figure legends.

**Reviewer Response (NCOMMS-25-42038B)**

We thank all reviewers for their thoughtful comments and are addressing all the suggested improvements below. Track changes was used to mark any changes in the revised manuscript.

**Reviewer #1:**
*The authors have addressed most of my concerns in this revised manuscript. I still take issue with the statement that TRIM21 KO cells "TRIM21KO THP-1 cells and NLRP3KO controls largely lacked polymerized ASC specks" (line 376). As shown in fig. 4a, about 4-5% of TRIM21KO cells still formed specks compared to 28% of control cells, while NLRP3KO cells failed to form specks. It would be more accurate to say that TRIM21KO THP-1 cells had reduced ASC specks compared to control cells.*

We reworded this sentence as suggested by the reviewer.

**Reviewer #2:**
We have read the revised manuscript with great interest. The Authors have made substantial efforts to enhance the quality of the work, resulting in significant improvement. We sincerely appreciate the additional experiments conducted to address our comments, as well as those raised by the other reviewers comments, as well as those raised by the other reviewers. All of our previous concerns have been satisfactorily addressed.

*Just one final suggestion for the Authors' consideration – as both we and the other reviewers identified several points that extend beyond the scope of the current study, which the Authors have indicated they intend to pursue in future work, it may be beneficial to include a dedicated paragraph outlining prospective directions for future research.*

As suggested, we included a paragraph at the end of the discussion with the future directions suggested by the reviewers.
